# Genomes shed light on the evolution of *Begonia*, a mega‐diverse genus

**DOI:** 10.1111/nph.17949

**Published:** 2022-02-09

**Authors:** Lingfei Li, Xiaoli Chen, Dongming Fang, Shanshan Dong, Xing Guo, Na Li, Lucia Campos‐Dominguez, Wenguang Wang, Yang Liu, Xiaoan Lang, Yang Peng, Daike Tian, Daniel C. Thomas, Weixue Mu, Min Liu, Chenyu Wu, Ting Yang, Suzhou Zhang, Leilei Yang, Jianfen Yang, Zhong‐Jian Liu, Liangsheng Zhang, Xingtan Zhang, Fei Chen, Yuannian Jiao, Yalong Guo, Mark Hughes, Wei Wang, Xiaofei Liu, Chunmei Zhong, Airong Li, Sunil Kumar Sahu, Huanming Yang, Ernest Wu, Joel Sharbrough, Michael Lisby, Xin Liu, Xun Xu, Douglas E. Soltis, Yves Van de Peer, Catherine Kidner, Shouzhou Zhang, Huan Liu

**Affiliations:** ^1^ Key Laboratory of Southern Subtropical Plant Diversity Fairy Lake Botanical Garden Shenzhen & Chinese Academy of Sciences Shenzhen 518004 China; ^2^ 213636 State Key Laboratory of Agricultural Genomics BGI‐Shenzhen Shenzhen 518083 China; ^3^ Institute of Molecular Plant Sciences University of Edinburgh Daniel Rutherford Building Max Born Crescent, The King's Buildings Edinburgh EH9 3BF UK; ^4^ Royal Botanic Garden Edinburgh 20a Inverleith Row Edinburgh EH3 5LR UK; ^5^ Xishuangbanna Tropical Botanical Garden Chinese Academy of Sciences Mengla Yunnan 666303 China; ^6^ Nanning Botanical Garden Nanning 530021 China; ^7^ Shanghai Chenshan Plant Science Research Center of Chinese Academy of Sciences Shanghai Chenshan Botanical Garden Shanghai 201602 China; ^8^ Singapore Botanic Gardens Singapore 259569 Singapore; ^9^ Key Laboratory of National Forestry and Grassland Administration for Orchid Conservation and Utilization Fujian Agriculture and Forestry University Fuzhou 350002 China; ^10^ College of Horticulture Academy for Advanced Interdisciplinary Studies Nanjing Agricultural University Nanjing 210095 China; ^11^ University of the Chinese Academy of Sciences Beijing 100049 China; ^12^ State Key Laboratory of Systematic and Evolutionary Botany Institute of Botany Chinese Academy of Sciences Beijing 100093 China; ^13^ 117866 Environmental Horticulture Research Institute Guangdong Academy of Agricultural Sciences Guangzhou 510640 China; ^14^ 12526 College of Forestry and Landscape Architecture Key Laboratory of Energy Plants Resource and Utilization Ministry of Agriculture, China South China Agricultural University Guangzhou 510642 China; ^15^ CAS Key Laboratory for Plant Diversity and Biogeography of East Asia Kunming Institute of Botany Chinese Academy of Sciences Kunming 650201 China; ^16^ Department of Biology University of British Columbia Vancouver BC V6T1Z4 Canada; ^17^ 7374 Biology Department New Mexico Institute of Mining and Technology Socorro NM 87801 USA; ^18^ 4321 Department of Biology University of Copenhagen Copenhagen DK‐2100 Denmark; ^19^ 213636 BGI‐Fuyang BGI‐Shenzhen Fuyang 236009 China; ^20^ 213636 Guangdong Provincial Key Laboratory of Genome Read and Write BGI‐Shenzhen Shenzhen 518083 China; ^21^ Florida Museum of Natural History University of Florida Gainesville FL 32611 USA; ^22^ Department of Plant Biotechnology and Bioinformatics (Ghent University) and Center for Plant Systems Biology (VIB) Ghent B‐9052 Belgium; ^23^ Center for Microbial Ecology and Genomics (CMEG) Department of Biochemistry, Genetics and Microbiology University of Pretoria Pretoria Hatfield 0028 South Africa

**Keywords:** *Begonia*, evolution, genomes, introgression, shade adaptation, whole‐genome duplication

## Abstract

- Clarifying the evolutionary processes underlying species diversification and adaptation is a key focus of evolutionary biology. *Begonia* (Begoniaceae) is one of the most species‐rich angiosperm genera with *c*. 2000 species, most of which are shade‐adapted.
- Here, we present chromosome‐scale genome assemblies for four species of *Begonia* (*B. loranthoide*s, *B. masoniana*, *B. darthvaderiana* and *B. peltatifolia*), and whole genome shotgun data for an additional 74 *Begonia* representatives to investigate lineage evolution and shade adaptation of the genus.
- The four genome assemblies range in size from 331.75 Mb (*B. peltatifolia*) to 799.83 Mb (*B. masoniana*), and harbor 22 059–23 444 protein‐coding genes. Synteny analysis revealed a lineage‐specific whole‐genome duplication (WGD) that occurred just before the diversification of *Begonia*. Functional enrichment of gene families retained after WGD highlights the significance of modified carbohydrate metabolism and photosynthesis possibly linked to shade adaptation in the genus, which is further supported by expansions of gene families involved in light perception and harvesting. Phylogenomic reconstructions and genomics studies indicate that genomic introgression has also played a role in the evolution of *Begonia*.
- Overall, this study provides valuable genomic resources for *Begonia* and suggests potential drivers underlying the diversity and adaptive evolution of this mega‐diverse clade.

## Introduction

The mechanisms underlying the diversification of large clades of closely related species (often designated taxonomically as genera) remain one of the biggest mysteries in plant biology (Frodin, 2004). Although speciose genera have received widespread attention from evolutionary biologists, typically few genomic resources are available for these closely related, species‐rich clades. Representative completely assembled nuclear genomes of only three of the 10 largest angiosperm genera (Frodin, 2004) have been published, namely *Solanum* (A. Bolger *et al*., 2014; Song *et al*., 2019), *Dendrobium* (Yan *et al*., 2015) and *Begonia* (Griesmann *et al*., 2018). However, these genomic studies either focused on the specific characteristics of the reference species (A. Bolger *et al*., 2014; Yan *et al*., 2015; Song *et al*., 2019) or were part of a large comparative genomic project (Griesmann *et al*., 2018); none of them used genomic data to explore the evolutionary patterns in these mega‐diverse clades.

**Fig. 1:**
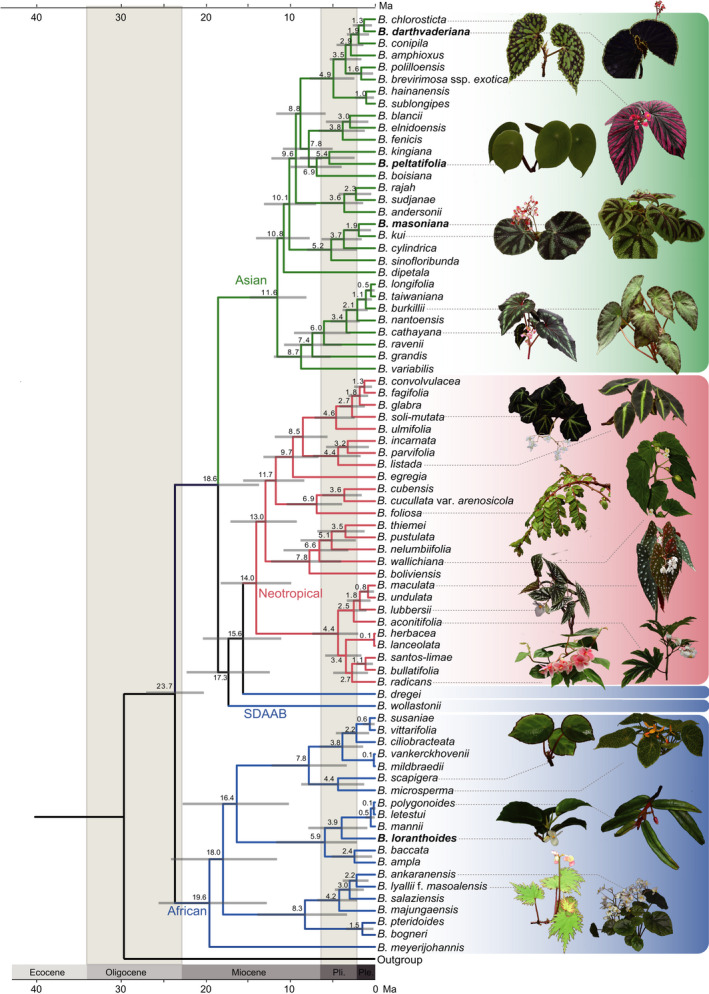
Phylogenetic tree showing the topology and divergence times for 78 newly sequenced species of *Begonia*. Maximum‐likelihood tree inferred with RAxML based on SNPs within regions of nuclear single‐copy genes. Divergence times are indicated at the internodes; the range of the blue bars indicates the 95% confidence interval of the divergence times. Representative images on the right show *Begonia* diversity. Three major geographically circumscribed clades are colored blue, red and green for the African, Neotropical and Asian clade, respectively. Taxa in bold highlight the four species with chromosome‐scale genomes generated in this study. Branches are maximally supported unless otherwise indicated.

*Begonia* L. (Begoniaceae, Cucurbitales) is well known for a huge diversity of leaf shapes, patterns and textures (Fig. 1). The genus is pantropical and comprises more than 2000 currently accepted species (Hughes *et al*., 2015) of herbs and occasionally subshrubs; it thus represents an excellent evolutionary study system for processes that generate numerous closely related species. Species of *Begonia* are mostly narrow endemics occupying specific microhabits. *Begonia* has high species diversity in the New World and Asia and relatively low species numbers in Africa, the continent of its putative origin (Neale *et al*., 2006). This high species diversity forms a stark contrast with its sister genus, the monotypic *Hillebrandia* Oliv. comprising the rare Haiwaiian endemic *H*. *sandwicensis*. Previous studies have suggested that the overall patterns of speciation in *Begonia* may be driven by local speciation in fragmented habitats (Hughes & Hollingsworth, 2008), hybridization and polyploidization (Dewitte *et al*., 2011).

Most begonias are shade‐adapted and become sun‐damaged when exposed to full sun. However, exceptions and intermediates regarding habitat preferences also exist. *Begonia* species exhibit a continuum of light adaptation ability ranging from deep shade to full sun, affording us the opportunity to unravel mechanisms of adaptation to cope with variable levels of light. Understanding how shade‐adapted species optimize photosynthesis and physical defense, while suppressing the shade‐avoidance syndrome (SAS; strong elongation growth away from shaded microconditions and accelerated flowering), will be valuable for crop improvement (Gommers *et al*., 2013). However, the genetic footprints and molecular basis of shade adaptation on the genome level remain elusive.

We *de novo* sequenced and assembled chromosome‐scale genomes of four *Begonia* species, and generated whole genome shotgun (WGS) data for an additional 74 *Begonia* species, representing 37 of the 70 recognized sections of *Begonia* (Moonlight *et al*., 2018) across all three major continental clades (Supporting Information Fig. S1). We compared the four *Begonia* genomes and reconstructed the paleogenome of *Begonia*, explored the evolutionary impact of a *Begonia*‐specific whole genome duplication (WGD) event (Brennan *et al*., 2012), analyzed the content of transposable elements (TEs), and analyzed their potential impact on the genomic landscape and potentially on species adaptation. We also examined cytonuclear incongruences detected in our study, and investigated the molecular basis of shade adaptation in *Begonia*.

The interpretation of *Begonia* genomic diversity in an evolutionary context will not only contribute to a better understanding of the origin, evolution and shade adaptation of this mega‐diverse clade, but also provide valuable reference genomes for molecular breeding of these highly valued ornamental plants.

## Materials and Methods

### Sample collections and DNA/RNA extraction

All *Begonia* samples were collected from the glasshouse in Fairy Lake Botanical Garden (Shenzhen, China) where plants were cultivated at 26°C : 18°C (day : night) with a relative humidity of 65–80%. Specimens have been deposited in the Herbarium of Fairy Lake Botanical Garden. For WGS, genomic DNA from young leaves of each individual was extracted using the cetyl trimethylammonium bromide (CTAB) method (Porebski *et al*., 1997). For single tube long fragment read (stLFR) sequencing, high‐molecular‐weight genomic DNA was isolated using the IrysPrep^®^ Plant Tissue DNA Isolation kit (RE‐014‐05; Bionano Genomics, San Diego, CA, USA) following the manufacturer’s instructions. DNA quality and quantity were evaluated using pulsed field gel electrophoresis and Qubit^®^ 3.0 Fluorometer (Thermo Fisher Scientific, Waltham, MA, USA). For transcriptome sequencing, total RNA from different tissues (root, stem/rhizome, leaf, peduncle and flower) from four Begonias were isolated using TRIzol^®^ reagent (Invitrogen, Carlsbad, CA, USA), respectively. All the RNA samples were quality controlled using a NanoDrop™ One UV‐Vis spectrophotometer (Thermo Fisher Scientific) and a Qubit^®^ 3.0 Fluorometer.

### Library preparation and sequencing

All DNA libraries for WGS were constructed using the MGIEasy FS DNA Library Prep Set (1000006988) with 300–500 bp fragment sizes, and sequenced on an Illumina Hiseq2000 platform to generate paired‐end (PE) reads of 150 bp. Transcriptome libraries were constructed with a MGIEasy RNA Library Prep Kit (1000006384) with inserts of 200–400 bp and sequenced with PE reads of 100 bp. More than 5 Gb of sequence data were generated for each library. The stLFR library was prepared with the MGIEasy stLFR Library Prep Kit (1000005622) (Wang *et al*., 2019) and sequenced with PE reads of 100 + 42 bp, generating > 150 Gb of raw sequence data for each library. 10 × Genomics Chromium™ Genome libraries with insert sizes of 350–500 bp were prepared with Chromium Genome Reagent Kit (v2 Chemistry, Pleasanton, CA, USA) following the manufacturer’s protocols with modified PCR primers to introduce sequencing primers suitable for the BGISEQ‐500 platform and then sequenced with PE reads of 150 bp. SMART library preparation and sequencing details are given in Methods S1.

### Genome assembly

For assembly of the sequences from 10 × Genomics Chromium and stLFR libraries, the clean reads were obtained using an in‐house script and *de novo* assembled using Spernova (v.2.1.1) (Weisenfeld *et al*., 2017) with default parameters. A minimum fasta record size of 100 bp was specified at the ‘mkoutput’ stage for outputting the assembly in the ‘pseudohap’ style. *De novo* assemblies of the PacBio long reads for *B. masoniana* and *B. darthvaderiana* were conducted by Canu (v.0.1) (Koren *et al*., 2017). Subsequently, two rounds of iterative corrections were performed with PacBio long reads using the software Racon (v.1.2.1) (Vaser *et al*., 2017), and two rounds of corrections with Pilon (v.1.22) (Walker *et al*., 2014) using 10 × Genomics reads (see details in Methods S1).

### Variant analysis

A total of 468 Gb 150 bp PE Illumina reads were generated, yielding an average coverage of 7× per accession. Raw reads were quality controlled using Trimmomatic (A. M. Bolger *et al*., 2014) to remove adaptors and low‐quality bases. The clean reads were aligned against the reference genome of *B. masoniana* using Bwa‐Mem (v.0.7.10) (Li, 2013) with default parameters. Variant detection was performed using the genome analysis toolkit (gatk; v.3.5‐0‐g36282e4) (Mckenna *et al*., 2010) following the best practices workflow for variant discovery. The resulting BAM files were locally realigned using the IndelRealigner to remove erroneous mismatches around small‐scale insertions and deletions. Variants were called in each accession separately using the HaplotypeCaller and individual gVCF files were merged using GenotypeGVCFs. This two‐step approach includes quality recalibration and regenotyping in the merged vcf file, ensuring variant accuracy. Single nucleotide polymorphisms (SNPs) were filtered based on the following criteria: SNPs in repeat regions; SNPs with read depth > 1000 or < 5; SNPs with missing rate > 40%; SNPs with < 5 bp distance with nearby variant sites; and nonbiallelic SNPs were removed. Phylogenetic reconstruction, admixture analysis, principal component analysis (PCA), diversity statistics and ABBA‐BABA analysis based on the SNP data are detailed in Methods S1.

### Phylogenetic analysis

For nuclear phylogenetic analysis, SNPs within 4000 *Begonia* single‐copy nuclear genes identified using the software OrthoFinder (Emms & Kelly, 2015) with four newly sequenced *Begonia* genomes with default settings were extracted from vcf files and filtered based on sequence length (> 100 bp) and taxon occurrences (> 50%), aligned with Mafft (Katoh *et al*., 2005), and trimmed with Gblocks (Talavera & Castresana, 2007). A supermatrix method was used to infer the nuclear phylogeny using RAxML (v.7.2.3) (Stamatakis, 2006). The maximum‐likelihood tree inferred from concatenated nuclear SNPs was used as a starting tree to estimate species divergence time using MCMC Tree as implemented in Paml (Yang, 2007). One calibration point of the *Begonia* crown group (24 million years ago (Ma) ± 3.57 million years with a normal distribution) was defined following Moonlight *et al*., 2018). For plastid phylogenetic analysis, we newly generated 78 *Begonia* plastid genomes with Novoplasty (Dierckxsens *et al*., 2017) using the seed sequence of *rbcL*. These plastid genomes were annotated and the conserved 83 plastid protein‐coding genes were extracted for phylogenetic inference in Geneious 10.0.2 (Biomatters, Auckland, New Zealand). The concatenated nucleotide dataset was evaluated with PartitionFinder (Lanfear *et al*., 2012) for the optimal data partition scheme and the associated nucleotide substitution models, with an initial partitioning strategy by both locus and codon positions, resulting in 13 partitions. The concatenated dataset was analyzed using RAxML (v.7.2.3) (Stamatakis, 2006) with 500 bootstrap replicates.

### Genome synteny

The syntenic blocks between two species were defined by Mcscan (Tang *et al*., 2008) based on core‐orthologous gene sets identified by Blastp (*E*‐value ≤ 1e‐5; number of gene pairs required to call synteny ≥ 5). The resulting dot plots were inspected to confirm the paleoploidy level of *Begonia* in relation to the other genomes by counting the syntenic depth in each genomic region. The synonymous *Ks* value for homologous gene pairs was calculated using the software Paml (Yang, 2007) and a custom perl script (https://ftp.cngb.org/pub/CNSA/data3/CNP0001056/CNS0227982/CNA0013976/), respectively (see details in Methods S1).

### Chlorophyll fluorescence analysis

For Chl fluorescence measurement, the plants were dark‐adapted for 30 min before the measurements with the MAXI version of the Imaging‐PAM M‐Series Chl fluorescence system (Heinz‐Walz Instruments, Effeltrich, Germany), as described by Jin *et al*. (2018). For measurements of the light‐response curves of photosystem II (PSII) quantum yield (Ф_PSII_), plant leaves were illuminated at the following light intensities: 0, 1, 21, 56, 111, 186, 281, 336, 396, 461, 531, 611, 701 and 801 μmol photons·m^−2^ s^−1^. The PSI electron transport rate (ETRI) was measured using light gradients of 0, 5, 13, 31, 89, 167, 209, 325, 496, 754 μmol photons m^−2^ s^−1^.

### Identification and phylogenetic analysis of light‐harvesting Chl a/b‐binding proteins superfamily

For the identification of LHCs, all LHCs previously described from *Arabidopsis* and *Oryza sativa* (Umate, 2010) were retrieved from the database of TAIR (www.arabidopsis.org) and NCBI (www.ncbi.nlm.nih.gov/protein/), respectively. Representative members of the subfamilies of *Arabidopsis* were used as queries to perform Blastp searches against the protein database of each species with an *E*‐value cutoff of 1e‐5. Candidate sequences identified as LHC orthologs were then aligned using Mafft (Katoh *et al*., 2005) to remove those that did not contain the intact domain (PF00504). For phylogenetic analysis, sequences of LHC orthologs of four Begonias, *Crocus sativus*, as well as *Arabidopsis thaliana* and *O. sativa* were aligned using Mafft (Katoh *et al*., 2005), followed by phylogenetic reconstruction with PhyML (v.3.1) (Guindon & Gascuel, 2003).

## Results

### Genome sequencing and genome characteristics

Seventy‐eight *Begonia* species were sequenced to acquire genome skim data for comparative genomic studies (Fig. 1; Table S1). As there is already a draft genome for the Neotropical *B. fuchsioides* (Griesmann *et al*., 2018), we selected four species, including one from Africa (*B. loranthoides*, 2*n* = 38) and three from Asia (*B. masoniana*, 2*n* = 30; *B. darthvaderiana*, 2*n* = 30; *B. peltatifolia*, 2*n* = 30), for reference genome sequencing (Fig. S2). K‐mer analyses based on 10 × Genomics Chromium reads data indicated an estimated genome size of *c*. 724, *c*. 806, *c*. 797 and *c*. 349 Mb for *B. loranthoides*, *B. masoniana*, *B. darthvaderiana* and *B. peltatifolia*, respectively (Table S2). The genomes of *B. masoniana* and *B. darthvaderiana* had the highest heterozygosity levels (Fig. S3), 0.96% and 0.98%, respectively, compared to 0.19% for *B. loranthoides* and 0.27% for *B. peltatifolia* (Table S3). We combined multiple sequencing and assembly technologies (Fig. S4; Table S4), including linked reads from stLFR and 10 × Genomics Chromium for four species, PacBio single‐molecule real‐time (SMRT) to assist in assembly of the more heterozygous *B. masoniana* and *B. darthvaderiana* genomes, and Hi‐C scaffolding strategies for chromosome assembly of all the four genomes. For *B. loranthoides* and *B. peltatifolia*, the genomes were assembled into 716.44 Mb (scaffold N50: 6.73 Mb) and 334.09 Mb (scaffold N50: 3.20 Mb) using 10 × Genomics data, with *c*. 88.55 and 87.13% of assembled sequences anchored onto 19 and 15 pseudochromosomes, respectively (Tables S4, S5). For *B. masoniana* and *B. darthvaderiana*, the genome assemblies yielded 799.40 Mb (contig N50: 0.44 Mb) and 771.67 Mb (contig N50: 0.32 Mb) using PacBio long reads data, with *c*. 98.83 and 97.55% of the assembled sequences anchored onto 15 pseudochromosomes, respectively (Table S5).

To evaluate the quality of the assemblies, RNA‐sequencing reads from root, stem/rhizome, flower, peduncle and leaf tissues were mapped to their cognate assemblies (Table S6). About 90.92–98.83% of the reads were aligned to their corresponding genomes (Table S7). The completeness of the assemblies in terms of gene content was assessed with Benchmarking Universal Single‐Copy Orthologs (Busco) analysis. Of the core 1614 conserved plant genes evaluated, 97.00, 91.00, 92.20 and 96.80% were complete in the assemblies for *B. loranthoides*, *B. masoniana*, *B. darthvaderiana* and *B. peltatifolia*, respectively; *c*. 0.80–2.50% of the genes were fragmented (Table S5). Collectively, these results demonstrated that our four genome assemblies were of high quality in terms of contiguity, base accuracy and genome completeness.

### Repeat annotation and gene prediction

**Fig. 2:**
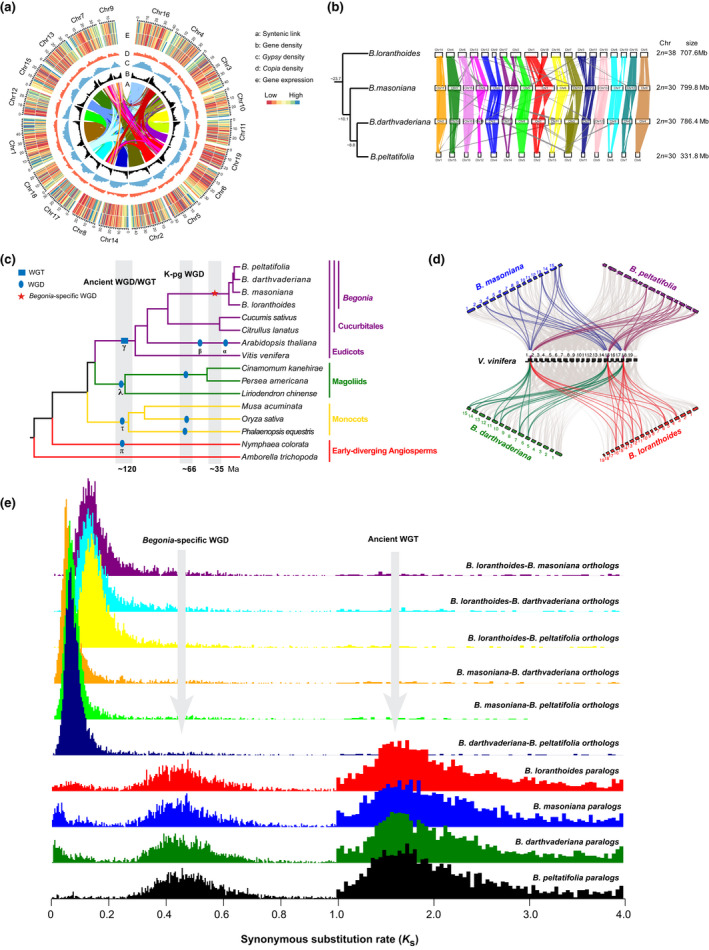
Synteny and lineage‐specific whole‐genome duplication (WGD) in *Begonia*. (a) Circular view of the *Begonia loranthoides* genome. a: Lines in the inner circle represent links between synteny‐selected paralogs. b: Gene density, c: *Gypsy* and d: *Copia* abundance, e: RNA expression of stem (outer) and leaf (inner). (b) Syntenic blocks in homologous chromosomes between *B. loranthoides*–*B. masoniana*, *B. masoniana*–*B. darthvaderiana*, and *B. darthvaderiana*–*B. peltatifolia*. (c) A simplified phylogenetic tree showing the lineage‐specific WGD in *Begonia*. The other generally accepted WGDs shown are based on Jiao *et al*. (2011) and Zhang *et al*. (2020). (d) Macrosynteny patterns show that three typical ancestral regions in the grape genome can be mapped to six regions in the *Begonia* genome. Gray wedges in the background highlight major syntenic blocks spanning > 30 genes between the genomes (highlighted by one syntenic set shown in color). (e) Synonymous substitution rate (*K_s_*) distributions of syntenic blocks for the paralogs of four Begonias and orthologs between either two Begonias are shown in different colors, as indicated. Note the *K_s_* unit in the range 1.0–4.0 is ten‐fold of that in the range 0–1.0.

Repetitive elements were estimated to represent 66.52%, 68.40%, 70.33% and 51.47% of the genome assemblies in *B. loranthoides*, *B. masoniana*, *B. darthvaderiana* and *B. peltatifolia*, respectively (Table S5). Most of these repeats were TEs that were further subclassified into nine groups (Table S8). Long‐terminal repeat retrotransposons (LTR‐RTs) represent 42.80–65.60% of the genome assembiles, with *Gypsy* elements being the most abundant transposon superfamily in all four *Begonia* species (30.39–48.60%), followed by the *Copia* superfamily (7.32–18.36%) (Table S8). The pattern of LTR distribution patterns varied across the genomes for different elements (Fig. 2a). The density of *Gypsy* scaled negatively with that of the genes whereas *Copia* was distributed more evenly across the genome and showed no obvious correlation with gene elements (Figs 2a, S5). This is expected since it is known that *Gypsy* elements accumulate predominantly in heterochromatin and centromeres, whereas *Copia* elements are normally scattered across the genome (Neumann *et al*., 2011).

*De novo* and homology‐based approaches were combined to predict protein‐coding genes. In total, 22 059, 22 861, 23 444 and 23 010 complete genes were predicted for *B. loranthoides*, *B. masoniana*, *B. darthvaderiana* and *B. peltatifolia*, respectively, with the highest gene density being 69 genes per Mb in *B. peltatifolia*, and 28–31 genes per Mb in the other three species (Table S5; Fig. S6), which correlated with the relatively small genome size of *B. peltatifolia* among the four analyzed *Begonia* species. The numbers of protein‐coding genes are relatively consistent within Cucurbitales (18 292–32 203), but except for *B. peltatifolia* the gene densities in *Begonia* (28–31 genes per Mb) are near two‐fold lower than those in Cucurbitaceae (64–117 genes per Mb), probably due to higher transposon content (Table S9).

### Whole genome duplication and gene evolution

The fraction of synonymous substitutions per synonymous site (*K_S_*) distributions of paralogs clearly showed two peaks (Fig. 2e), one around 1.5 representing the γ hexaploidization event shared by the core eudicots (Jaillon *et al*., 2007; Chanderbali *et al*., 2017), and the other around 0.5 indicating that a lineage‐specific WGD event occurred in *Begonia*. By performing a comparative genomic analysis of *Begonia* with *Vitis vinifera*, we identified a 2 : 1 syntenic depth ratio (Fig. 2b), which confirms the WGD previously reported in Brennan *et al*. (2012). We speculate that the WGD event occurred 35 ± 8 Ma (Fig. 2c; Methods S1), and hence before the split of *Begonia* (median, *c*. 25 Ma) and its monotypic sister *Hillebrandia*, the only other genus of Begoniaceae (Moonlight *et al*., 2018). This is supported by the fact that *Hillebrandia* has also been found to possess the WGD, probably indeed shared with *Begonia* (Martínez, 2017). Following the WGD event, 2850 gene families were retained in the common ancestor of the four species of *Begonia* we sequenced. The retained gene duplicates shared by the four species were considered as core retained genes. This set was enriched for terms such as ‘carbohydrate biosynthetic process’ and ‘nucleotide binding’, and many metabolism pathways such as ‘inositol phosphate metabolism’, ‘starch and sucrose metabolism’, and ‘galactose metabolism’ (Fig. S7).

Individual species retained some specific groups of duplicated genes (Figs S8–S11); for instance, genes annotated as involved in the ‘cutin, suberine and wax biosynthesis’ pathway were differentially retained in *B. loranthoides*. This gene retention might be associated with the characteristic waxy leaves of this species. Specific retention of genes involved in ‘phenylpropanoid biosynthesis’ and ‘flavonoid biosynthesis’ might be responsible for the colorful leaves of *B. masoniana* and *B. darthvaderiana* (Fig. S2). As variegated leaves are commonly found among Begonias and are largely attributed to the accumulation of anthocyanins, we looked more closely at the anthocyanin biosynthesis pathway gene families. We found that in contrast to expansion of gene families of the upstream general phenylpropanoid pathway from Curcubitaceae, *Begonia* species show significant expansion of gene families related to anthocyanin biosynthesis, especially for *Chalcone synthase* (*CHS*) in *B. masoniana* and *Flavanone 3‐hydroxylase* (*F3H*) and *Dihydroflavonol 4‐reductase* (*DFR*) in *B. darthvaderiana* (Fig. S12), and there is recent evidence for relaxed selective constraints and differential expression of paralogs in *CHS* in *Begonia* (Emelianova *et al*., 2021).

Based on a high‐confidence phylogenetic tree reconstructed by 193 single‐copy nuclear gene families of 13 angiosperm species including *V. vinifera*, *Populus trichocarpa*, *Glycine max*, *Prunus mume* and five species from Cucurbitaceae, we identified gene families that have experienced significant expansions and contractions during the evolution of *Begonia* and related species (Fig. S13). Twenty gene families, including 1071 genes, were significantly expanded (*P* < 0.05) in *Begonia* species compared to the other groups. GO and KEGG enrichment analyses found these to be enriched in terms including ‘zinc ion binding’, ‘transition metal ion binding’ and ‘metal ion binding’ (Table S10), which are primarily involved in the ‘Oxidative phosphorylation’, ‘Endocytosis’ and ‘Pyrimidine metabolism’ pathways (Table S11). Surprisingly, many resistance‐ and defense‐related gene families such as ‘NB‐ARC’ were significantly contracted in the *Begonia* lineage. The TIR‐NBS‐LRR (TNL) disease resistance gene family (Kim *et al*., 2009) was completely lost (Fig. S14). We looked for expansion of other disease‐related genes and found that only the Autophagy 17 (APG17) family showed significant expansion in *Begonia* (Table S12). Other GO terms which were underrepresented in the set of contraction gene families included the ‘protein kinase domain’, ‘Cytochrome p450’ and ‘Terpene synthase’ gene families (Table S13).

### Chromosome evolution

To reconstruct the evolutionary events leading to current genome structures in *Begonia*, homologous chromosome segments between different species were identified. There were 122 shared syntenic blocks in the four species of *Begonia* sequenced here, which accounted for 74.6%, 78.6%, 67.0% and 74.6% of the *B. loranthoides*, *B. masoniana*, *B. darthvaderiana* and *B. peltatifolia* genomes, respectively. The lowest percentage of syntenic blocks in *B. darthvaderiana* among those of the four species was consistent with the low TE proportion in these regions compared to *B. masoniana* and *B. loranthoides* (Fig. S15). Synteny analyses between them showed that each chromosome had a nearly one‐to‐one syntenic relationship with chromosomes from other species (Fig. 2b); the relationship was especially strong for those three species with the same chromosome number. Some large inversions could be inferred for each species. One translocation was detected in chromosomes 2 and 17 in *B. loranthoides*. Chromosome fissions and fusions were identified in the genomes of *B. loranthoides* and *B. masoniana*. We suggest that chr9 and chr12, chr1 and chr18, chr3 and chr11, and chr8 and chr19 in *B. loranthoides* experienced breakage with fusion to chr4, chr1, chr11 and chr15 in *B. masoniana*, respectively (Fig. 2b).

Conserved gene adjacencies suggest that the ancestral *Begonia* karyotype reconstructed based on the four species noted above consisted of 22 conserved ancestral regions (CARs), following an ancestral WGD that occurred early in the history of Begoniaceae characterizing all extant members (Fig. S16). From the 22 CARs of the ancestral karyotype, the 15 chromosomes of *B. masoniana* might be derived by three fusions, and four deletions, while the 15 chromosomes of *B. darthvaderiana* were shaped through one fission, four fusions and three deletions, the 15 chromosomes of *B. peltatifolia* through four fissions and 11 fusions, and the 19 *B. loranthoides* chromosomes through seven fissions and 10 fusions (Fig. S16). Although *B. peltatifolia* has the same chromosome number as *B. masoniana* and *B. darthvaderiana*, it appears to have undergone a large number of chromosome fissions and fusions after the split from their common ancestor. This suggests that genomic rearrangements may be even more frequent in *Begonia* than apparent from the highly variable chromosome numbers (2*n* = 16–156) (Dewitte *et al*., 2009, 2011).

### Transposable elements evolution and distribution

Transposable elements generally comprise the bulk of plant genomic DNA and their numbers show a positive correlation with genome size (Wendel *et al*., 2016). In our *Begonia* samples, this also appears to be the case: *B. peltatifolia* has both the smallest genome and the smallest number and proportion of TEs (Fig. S17). Amongst the most abundant superfamilies of TEs, the number of *Gypsy* and *Copia* LTR elements were most strongly and positively correlated with genome size (Fig. S17). As the four *Begonia* species have similar numbers of protein‐coding genes (Table S5), genome size variations between them are essentially attributed to the variation of TE abundance between the different *Begonia* species.

**Fig. 3:**
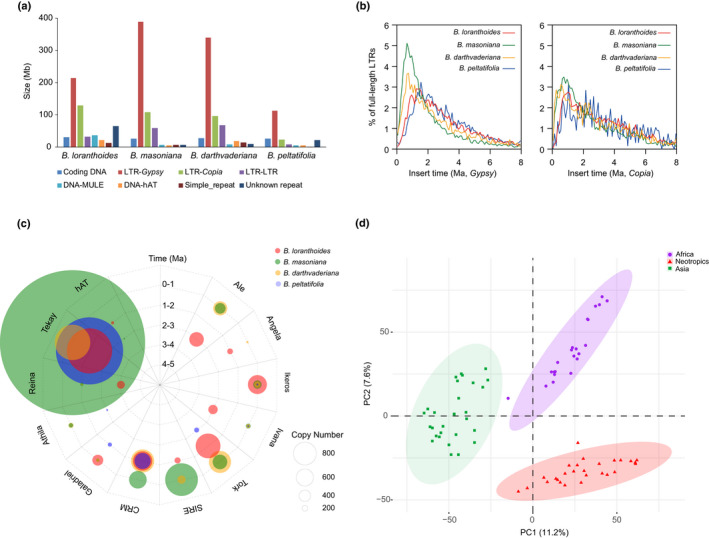
Transposable element (TE) evolution in the *Begonia* genomes. (a) TE composition of the four *Begonia* genomes. Stacked histograms represent the contribution of each TE superfamily to the four genomes. The eight most abundant TE superfamilies are shown. (b) Estimation of insertion time of *Gypsy* and *Copia* based on analyses of full‐length LTRs in four *Begonia* species. (c) Activity of 12 prominent full‐length retrotransposon families in four *Begonia* species. Concentric circles indicate timescale per million years from 6 Ma (center) to the present (outer circle). For each family, the circle size shows the retrotransposon number in four species. Each data point represents the peak activity of that element. (d) Principal component analysis (PCA) based on TE abundances of 74 *Begonia* species originated from Africa (green), America (orange) and Asia (red).

The investigation of TE representation in our four *Begonia* genome assemblies showed that they had different compositions of TE superfamilies (Fig. 3a; Table S8), and are quite variable for full ‐length *Gypsy* and *Copia* families (Fig. S18). The analysis of full‐length LTR‐RTs indicated several transposon bursts occurred during the last 8 million years, including recent expansions in all species, especially in *Gypsy* elements compared to *Copia* (Fig. 3b). When the full‐length *Gypsy* and *Copia* families were analyzed in the four *Begonia* species, they showed an expansion event at 0–2 Ma, with the *Reina* subgroup of elements expanding 3–4 Ma in *B. loranthoides* (Fig. 3c).

To determine the historical dynamics of the different lineages of *Gypsy* and *Copia* elements in the *Begonia* genomes, the divergence of their reverse transcriptase (RT) sequences was analyzed. Evolutionary analysis revealed different patterns among different LTR lineages in the four species (Fig. S19). For example, *SIRE* elements of *Copia* show a recent activity burst from a few ancestor sequences in *B. masoniana*, *B. loranthoides* and *B. darthvaderiana*, but no burst is observed in *B. peltatifolia*. A few species‐specific bursts were also observed for the *Gypsy Tekay* element in all four genomes investigated (Fig. S19). Furthermore, several *Copia Ivana* and *Gypsy CRM* copies from the common ancestor of the four *Begonia* species we investigated have been maintained and are still active (Fig. S19). These findings show that *Begonia* has a long and ongoing history of active TE elements.

Based on the presence and abundance of TE elements in each species, PCA recovered three well‐circumscribed *Begonia* lineages, corresponding to the three major geographical groups of the genus, indicating similar TE compositions in geographically restricted clades (Fig. 3d). Closely related species showed similar TE abundance, even in some species that have diverged more than 10 Ma (Fig. S20). Our investigation reveals a congruence of TE abundance with the phylogenetic tree, indicating that TEs are specifically accumulating across clades of species.

To look for effects of TE activity on gene function, we analyzed TE distribution upstream and downstream of genes. The numbers of genes with adjacent *Copia* and *Gypsy* elements insertions was very similar for the four *Begonia* species (Fig. S21a). However, comparison of other enriched TE families surrounding genes indicated different composition patterns in these four *Begonia* species (Fig. S21b). About 743–2751 (3.23–12.03%) and 1705–2378 (7.41–10.78%) genes have TE insertions in their intron and promoter regions, respectively (Fig. S22). Functional enrichment analysis of those genes with TE insertions identified stress‐related and metabolic process pathways as over‐represented in the set (Tables S14, S15), with distinct differences between the basal African lineage represented by *B. loranthoides* and the three Asian species (Fig. S23).

### Evolution of shade adaptation

**Fig. 4:**
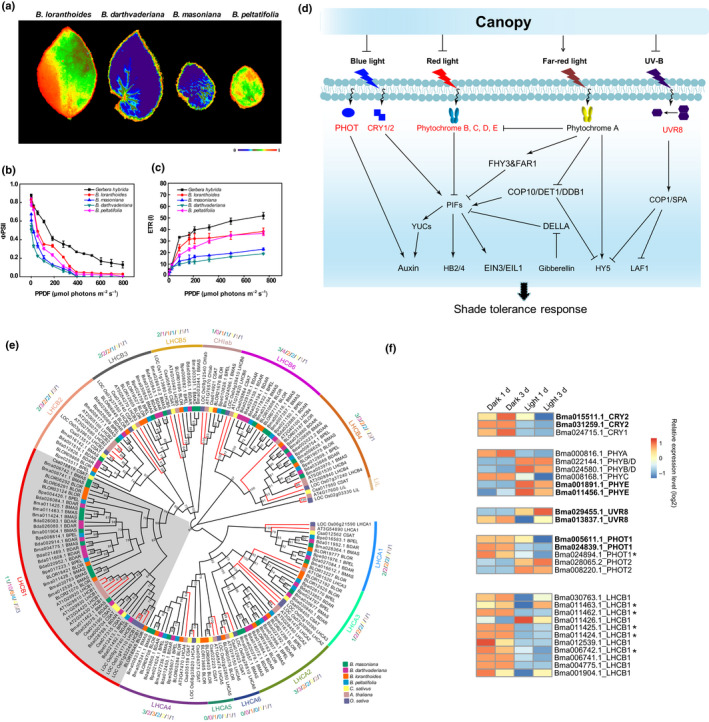
Shade adaptation in *Begonia*. (a) False‐color images representing maximum photochemical efficiency of PSII (*F*_v_/*F*_m_) under growth light conditions in four different *Begonia* species. The false‐color scale ranges from black (0) to red (1) as indicated below the false‐color images. Light‐response curves of PSII quantum yield (ΦPSII) (b) and electron transport rate of PSI (ETRI) (c) of four Begonias and *Gerbera hybrida*. Four biological replicates were performed in all experiments, and values are given as mean ± SD. (d) Simplified overview of the signal transduction pathway of shade adaptation response modified from Gommers *et al*. (2013) and Podolec & Ulm (2018). Arrows and blunt arrows indicate positive and negative regulations, respectively. The expansion genes in *Begonia* are labeled in red. Full names of gene abbreviations are given in Table S17. (e) Phylogenetic tree of light‐harvesting Chl*a*/*b*‐binding protein (LHC) superfamily show expansion of LHCB in two shade Begonias. Branch of LHCB1 in gray shows prominent expansion. Numbers corresponding to different species are shown beside each subfamily. (f) Expression patterns of photoreceptors and LHCB1 family genes under light and dark treatment. The lineage‐specific whole‐genome duplication retained genes are labeled in bold, and genes marked by an asterisk are derived from tandem duplication.

As classical shade‐dwelling plants, all Begonias have lower total Chl and lower ratios of Chl*a*/*b* compared with those of a typical sun‐exposed plant such as *Gerbera hybrida* (Table S16). Through comparative genomic analysis, we found several gene families belonging to the core components of light perception; that is, Cryptochromes (CRYs), Phototropins (PHOTs), Phytochromes and UV Resistance Locus 8 (UVR8) were obviously expanded in *Begonia* following the lineage‐specific WGD event compared to other plant species (Figs 4d, S24–S27; Table S17). Furthermore, we found that all these lineage‐specific WGD‐retained photoreceptor genes of *B. masoniana* displayed differential expression responding to light and dark treatment. Notably, the two retained copies of *UVR8* showed divergent expression between light and dark (Fig. 4f). Thus, a higher copy number of these genes might contribute to shade adaptation by increasing the complexity of the light response regulation network (Wu *et al*., 2019).

Although most species of *Begonia* are shade‐tolerant plants, the extent of shade adaptation varies: *B*. *masoniana* and *B. darthvaderiana* are deep shade plants from karst limestone cave habitats and the interior of tropical rainforests, respectively, whereas *B. loranthoides* and *B. peltatifolia* are acclimated to semishaded and more open environments. Based on phylogenetic relationships, the deep shade adaptations of *B. masoniana* and *B. darthvaderiana* are independent events (Fig. 1). As expected, the two deep shade species (*B. masoniana* and *B. darthvaderiana*) had significantly lower levels of Chl and lower Chl*a*/*b* ratios (Table S16), along with lower maximum photochemical efficiency of PSII (*F*_v_/*F*_m_) and quantum yield than the two semishade species (Fig. 4a–c). Comparative gene family analyses revealed significant expansions of the LHCs family in the two ‘deep shade’ Begonias (Fig. 4e; Table S18). Notably LHCB, and especially the LHCB1 subgroup, show prominent expansions in these two shade‐dwelling species due to parallel tandem duplications (Figs 4e, S28). All the duplicated LHCB1 gene pairs showed upregulation in the dark, and downregulation in the light (Fig. 4f), which may indicate their strengthened ability of light harvesting under low light. Together, these results suggest that both WGD‐ and tandem‐driven photoreceptors and light‐harvesting genes contribute to shade adaptation of *Begonia*.

### Genetic variation and admixture patterns

**Fig. 5:**
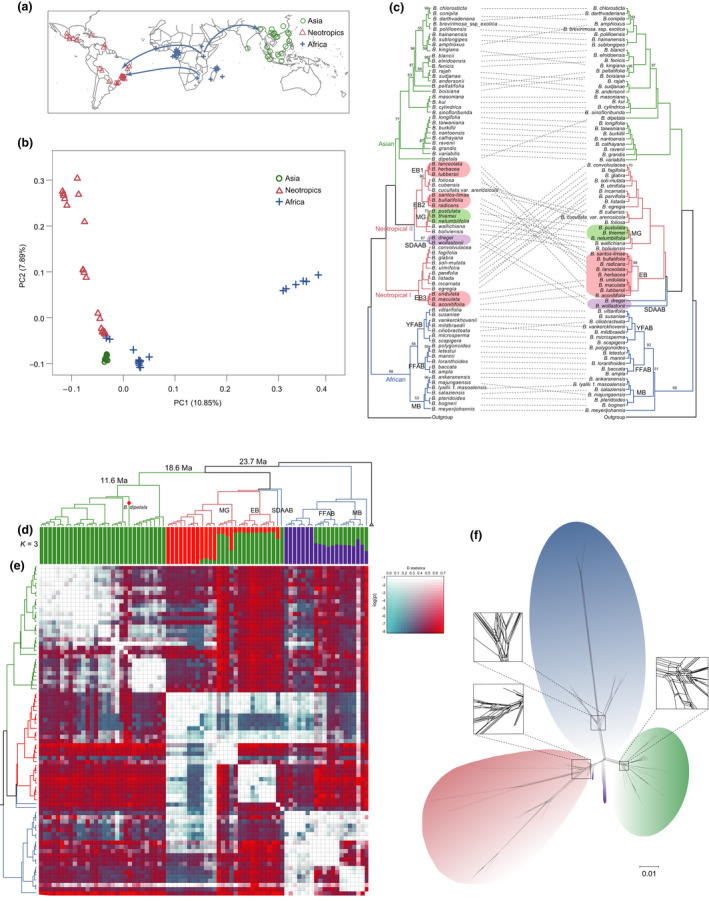
Phylogenomic incongruences and hybridization. (a) Geographic localities of sequenced *Begonia* individuals and a proposed migration route. (b) Principal component analysis (PCA) of the sequenced 78 *Begonia* accessions. (c) Cytonuclear conflicts between chloroplast (left) and nuclear (right) phylogenetic trees among 78 sequenced *Begonia* species with three Cucurbitales species as the outgroup. Branches are maximally supported unless otherwise indicated. (d) Ancestry results from Admixture under the *K* = 3 model supported by an examination of cross‐validation. (e) Detection of introgression events using Patterson’s *D* statistics among different *Begonia* species. (f) SplitsTree network for 78 *Begonia* individuals. MB, Malagasy *Begonia*; EB, East Brazil; YFAB, Yellow‐flowered African *Begonia*; FFAB, Fleshy‐fruited African *Begonia*; SDAAB, Seasonally dry adapted African *Begonia*; MG, Mexico section *Gireoudia*.

*Begonia* originated in Africa and spread across all the tropical regions except Australia (Neale *et al*., 2006). We selected 78 accessions (Fig. 5a) that cover the full distribution of *Begonia*, representing 37 out of 70 sections, to investigate patterns of genetic variation across the genus. We detected 1 137 696 SNPs and 66 862 small indel variants (< 10 bp). Phylogenetic analysis using a subset of 926 407 SNPs within regions of putatively single‐copy genes (SCGs) clearly differentiate *Begonia* accessions into three distinct clades (Fig. 5c). A weakly supported African clade is sister to a clade consisting of two monophyletic lineages including one consisting of largely Neotropical accessions and one consisting of exclusively Asian accessions.

Genetic clustering analysis with Admixture showed an optimal value of *K* = 3 subpopulations (Fig. 5d), which is consistent with the PCA (Fig. 5b). We observed evidence of interspecific admixture within the Neotropical and African *Begonia* accessions, respectively, and the highest nucleotide diversity (*π*) in the Neotropical accessions (0.0005755) compared with that of the African (0.0002595) and Asian (0.0002434) accessions (Fig. S29).

### Phylogenomic incongruences and hybridizations

Species of *Begonia* are known to hybridize in nature (Peng & Ku, 2009; Hughes *et al*., 2018), and previous work (Goodall‐Copestake *et al*., 2010) identified possible hybridization events early in *Begonia* evolution. To investigate this further we compared phylogenetic inferences between plastid and nuclear phylogenies. The plastid tree supports the African origin of *Begonia* and shows successive divergences of four major clades, corresponding to the African, Neotropical I, Neotropical II and Asian clades (Figs 5c, S30, S31). Our plastid phylogeny differs from previous phylogenetic studies based on three plastid markers (Moonlight *et al*., 2018) in the position of the yellow‐flowered African *Begonia* (YFAB) clade. The YFAB clade forms a sister group with the Fleshy‐fruited African *Begonia* (FFAB) clade in our study (Fig. 5c) whereas in previous multilocus studies it diverged at the base of *Begonia* (Moonlight *et al*., 2018). The nuclear trees (Figs S32–S34) and TE topology (Fig. S20) in our study consistently recovered a topology with three major geographically restricted clades: the African, Neotropical and Asian clades. Some conspicuous incongruences between nuclear and plastid trees can be identified within the Neotropical clade: the well‐resolved EB (East Brazil) clade containing sections *Trachelocarpus*, *Pereira*, *Astronthrix*, *Solananthera*, *Gaerdtia* and *Latistigma* in the nuclear tree is split into three independent lineages (EB1, EB2, EB3) diffusely distributed between the two Neotropical clades in the plastid tree (Fig. 5c). The position of the two SDAAB (Seasonally dry adapted African *Begonia*) accessions also show strong cytonuclear incongruence, suggesting hybridization, introgression or incomplete lineage sorting (ILS).

We observed strong discordance for the Neotropical species in the species tree constructed with Astral‐III (Fig. S32). Hybridization or ILS are possible explanations for this and are also suggested by the Splitstree network analysis which revealed a reticulate evolution for these Neotropical accessions (Fig. 5f). To identify possible causes of genetic introgression among *Begonia* species, we calculated Patterson’s D‐statistics for every triplet (a combination of P1, P2 and P3) in the *Begonia* phylogeny. The ABBA‐BABA analyses revealed significant introgression in the Neotropical clade between the lineage containing MG (Mexico sect. *Gireoudia*), and the aforementioned EB clade, the EB clade and the African SDAAB *B*. *wollastonii*. We also observed strong genetic introgression between the SDAAB accession *B. dregei* and the MG clade (Fig. 5e), suggesting that hybridization and introgression might play some roles in the evolution of the Neotropical *Begonia*. Phylonet network results suggested a hybrid origin of a clade consisting of *B. bullatifolia* and *B*. *santos‐limae* (from the EB clade) from *B. radicans* and the early Neotropical *Begonia* colonizers, as well as a hybrid origin of the lineage consisting of Sect. *Wageneria* and *B*. *soli‐mulata* (Fig. S35), lending support for the ABBA‐BABA introgression results.

In contrast to extensive cytonuclear incongruence and putative hybridization and introgression in the Neotropical clade, only a few topological incongruences were detected within Asian and African clades (Fig. 5c). Nonetheless, significant introgression might have occurred between the MB clade and the FFAB clade (Fig. 5e). One independent introgression event was also inferred for the Asian clade, namely the introgression between *B. dipetala* and the ancestor of the other Asian Begonias (Fig. 5e). These introgressions were also supported by the corresponding Phylonet network results (Fig. S35). Together, these putative introgression and hybridization events are generally in good aggreement with the instances of cytonuclear incongruence and may have played a role in the evolution of *Begonia*.

## Discussion

Putative WGDs have been identified across the eukaryote tree of life, especially in the green plant clade. Many of these WGDs are considered as driving forces contributing to species diversification and evolutionary innovations (Van de Peer *et al*., 2017; Ren *et al*., 2018; Leebens‐Mack *et al*., 2019; Wu *et al*., 2019). WGD may be followed by lineage‐specific loss of duplicated genes, contributing to adaptation to new niches, survival in response to environmental stress and subsequent rapid accumulations of species diversity (Landis *et al*., 2018; Ren *et al*., 2018; Van de Peer *et al*., 2021). In this study, we confirmed the occurrence of a lineage‐specific WGD event in the common ancestor of Begoniaceae (*c*. 35 Ma), before the split of cosmopolitan *Begonia* (median, *c*. 25 Ma) from the Hawaiian endemic *Hillebrandia* (Moonlight *et al*., 2018) (Fig. 2c). As a shade plant, shade adaptation is the key driving force underlying the diversification of *Begonia*. We provide evidence that the expansion of light signaling pathway genes retained following WGD may have contributed to shade adaptation of *Begonia* (Fig. 4).

However, WGD is not always associated with species diversification (Landis *et al*., 2018), as shown in the stark contrast of species diversity between the two genera. The present lack of species diversity in *Hillebrandia* on the Hawaiian Archipelago is potentially linked to its relict status on the older islands (Clement *et al*., 2004) and highly homozygous genome (Martínez, 2017). It is tempting to speculate that *H. sandwicensis* is a dying ember of a once much more species‐rich clade, with diversity having been extinguished in the scramble to colonize the archipelago as islands sank and emerged during its geological evolution.

In addition to WGD, hybridization and introgression have also contributed to the species diversity of *Begonia*. Through population genomic analysis, we detected several putative hybridization events, especially in the Neotropical clade (Fig. 5). These events may have partially contributed to the exceptional species diversity and genetic diversity of Neotropical *Begonia* though novel combinations of genotypes, introgression and hybridization‐based genome rearrangements or TE activation. Further genomic studies on Neotropical *Begonia* might help elucidate which factors have contributed to this high species diversity.

Plant genomes tend to accumulate large amounts of LTRs, and these have been shown to create different landscapes across closely related taxa. The presence and activity of TEs in plant genomes has been widely observed in many other plant groups, from largely studied taxa such Brassicaceae (Joly‐Lopez & Bureau, 2014; Rogivue *et al*., 2019), Solanaceae (Parisod *et al*., 2012; de Assis *et al*., 2020) and Poaceae (Ma *et al*., 2004; Altinkut *et al*., 2006; Wyler *et al*., 2020), to nonmodel plant groups such as *Quercus* (Mascagni *et al*., 2019), *Passiflora* (Sader *et al*., 2021), *Anacyclus* (Vitales *et al*., 2019) or *Melampodium* (McCann *et al*., 2020), among many others. We show that transposons are also an important source of genetic variation in *Begonia*. Two thousand genes in *Begonia* genomes have TE insertions in their promoter regions (Fig. S22). KEGG functional annotations of these genes with TE insertions in the promoter regions revealed a similar pattern for the three Asian Begonias with enrichment in the pathways of carbohydrate and energy metabolism (Fig. S23). This consistency suggested that TE insertions in the promoter regions might be under some selection constraints rather than neutral and random processes (Baduel *et al*., 2019). Moreover, the GO enrichment analyses found these genes with TE insertions to be specifically enriched in the function of photosynthesis, negative regulation processes, response to biotic stimulus and stress, and defense response (Tables S14, S15). This result suggests that TE insertions into the regulatory regions in *Begonia* genomes might play some adaptive role, as has been demonstrated in *Arabidopsis* (Li *et al*., 2018; Baduel *et al*., 2019) and maize (Freeling *et al*., 2015).

In summary, we have assembled for the first time four chromosome‐level genome assemblies of *Begonia*, and also provide WGS data for 74 representative species within the genus. Through comparative genomics, we confirmed that a lineage‐specific WGD event pre‐dates the radiation of *Begonia* and may have provided substantial genetic materials for the phenotypic evolution and shade adaptation. Moreover, we found considerable variation in the compositions and abundance of TEs, and strong phylogenetic signal in TE feature clustering. Species‐specific patterns of TE insertions in promoters and introns might have played a role in the adaptative evolution of *Begonia*. Furthermore, we provide evidence for introgression during the evolution of *Begonia*, especially for the Neotropical clade. This study not only provides high‐quality genomic resources for *Begonia*, but also reveals new insights into the evolution mechanisms of a mega‐diverse clade.

## Author contributions

SZ and HL conceived and initiated the study. LL, XC, SD and XG designed the major scientific objectives and managed the project with SZ, HL and DT. HY, XX and Xin Liu coordinated the project. XC, CW, Wenguang Wang and LL conducted the sequencing experiments. DF performed the genome assembly and annotation; XC, LL and SD carried out the repeat analysis. LL, NL, DF, XC, MLiu, WM, ZJL, LZ, TY and JS carried out the comparative genomic analysis and analyzed the gene families. DF, LL, NL, XC, SD, YJ and FC were involved in the WGD analysis. DF, LL, XC, SD, LCD, XG and YG performed the gene annotation and transposable element analysis. SD, XG, NL, DF, XZ, AL, EW and Wei Wang coordinated the phylogenetic analysis. SD, YL, DF, SKS and XC annotated the chloroplast genomes. LL, Xiaofei Liu, CZ and XLang performed Chl fluorescence measurements. LL, YP and LY contribute to cytology analysis. SZ, XLang, Wenguang Wang, Suzhou Zhang, JY and LL coordinated and collected the samples. LL, DF, XG, SD and XC drew and modified the figures. LL, XC, SD, XG, DF, NL, CK, LCD, DCT, DES, YVdP, MH and MLisby wrote and edited most of the manuscript. All authors read and approved the final manuscript. LL, XL, DF, SD, XG and NL contributed equally to this work.

## Supporting information


**Fig. S1** Current whole genome shotgun (WGS) sampling of *Begonia* accessions (78 individuals in 37 sections, as marked in red) on the sectional‐level *Begonia* phylogeny by Moonlight *et al*. (2018).
**Fig. S2** Somatic chromosome counts at metaphase in the four sequenced *Begonia* species.
**Fig. S3** K‐mer analyses of the four *Begonia* species.
**Fig. S4** Flowchart of sequencing and assembly for the four *Begonia* species.
**Fig. S5** Scaffold collinear comparisons between two different species (upper: *B. masoniana*, bottom: *B. peltatifolia*) show distinct distributions of different transposon elements.
**Fig. S6** Distribution of gene density of four *Begonia* genomes.
**Fig. S7** Analyses of post‐whole‐genome duplication (WGD) retained gene families in *Begonia*.
**Fig. S8** Analysis of post‐WGD retained gene families specific to *B. loranthoides*.
**Fig. S9** Analysis of post‐WGD retained gene families specific to *B. masoniana*.
**Fig. S10** Analysis of post‐WGD retained gene families specific to *B. darthvaderiana*.
**Fig. S11** Analysis of post‐WGD retained gene families specific to *B. peltatifolia*.
**Fig. S12** Expansion of gene families in anthocyanin pathway in *Begonia*.
**Fig. S13** Gene family expansions and contractions along a dated angiosperm phylogeny of 13 selected species.
**Fig. S14** Contraction and complete loss of the TNL subgroup of the NBS family in *Begonia*.
**Fig. S15** Comparison of TE proportions in 122 shared syntenic blocks across four *Begonia* species.
**Fig. S16** Reconstruction of the paleogenome of four sequenced *Begonia* species.
**Fig. S17** Number of LTR insertions and genome sizes for 13 angiosperm species.
**Fig. S18** Number of shared full‐length LTR families across four *Begonia* species.
**Fig. S19** Neighbour‐joining trees built from RT domain sequence similarities among different lineage‐specific copies identified in *Begonia* genomes.
**Fig. S20** Comparison of nuclear ML tree and abundance clustering of TEs.
**Fig. S21** The TE landscape surrounding genes in four *Begonia* species.
**Fig. S22** Impacts of TE insertions on the structure of introns and promoters.
**Fig. S23** KEGG enrichment of genes with TE insertion either in introns or in promoters.
**Fig. S24** Expansion of Cryptochrome (CRYs) genes in *Begonia* due to WGD.
**Fig. S25** Expansion of Phototropin (PHOT) genes in *Begonia* due to WGD.
**Fig. S26** Expansion of Phytochrome (PHY) genes in *Begonia* due to WGD.
**Fig. S27** Expansion of UV Resistance Locus 8 (UVR8) genes in *Begonia* due to WGD.
**Fig. S28** Schematic diagrams show tandem duplication of LHCB1 genes in *B. masoniana* and *B. darthvaderiana*.
**Fig. S29** Nucleotide diversity (π) and population divergence (*F*_ST_) across the three major groups of *Begonia*.
**Fig. S30** Maximum‐likelihood tree inferred from concatenated nucleotide sequences of *Begonia* plastid protein coding genes using RAxML.
**Fig. S31** Maximum‐likelihood tree inferred from *Begonia* plastome nucleotide alignment of 156 131 bp using RAxML.
**Fig. S32** Maximum‐likelihood tree inferred from a concatenated dataset of 1604 nuclear genes using IQtree with individual gene trees mapped.
**Fig. S33** Coalescent super tree inferred with Astral‐III using 1604 nuclear single gene trees.
**Fig. S34** Coalescent super tree inferred with Astral‐III using SNPs in 1343 nuclear single gene trees.
**Fig**. **S35** Phylonet network results for three geographically delimited *Begonia* clades.
**Methods S1** Supplemental methods.

**Table S1** Summary of 78 *Begonia* species for whole genome shotgun sequencing.
**Table S2** Genome size estimation based on K‐mer analysis.
**Table S3** Summary of within‐genome heterozygosity of the four *Begonia* species.
**Table S4** Statistics of genome assemblies.
**Table S5** Global statistics of genome assembly and annotation of four *Begonia* species.
**Table S6** Statistics of raw data for whole genome sequencing and RNA‐seq.
**Table S7** Statistics of reads mapping to genome sequences for RNA‐seq data from different tissues for four *Begonia* species.
**Table S8** Repetitive elements in four *Begonia* genomes.
**Table S9** Summary of genome information across 13 representative angiosperms.
**Table S10** Gene ontology (GO) term enrichment analysis of the expanded gene families of *Begonia*.
**Table S11** Kyoto encyclopedia of genes and genomes (KEGG) enrichment analysis of the expanded gene families of *Begonia*.
**Table S12** Number of genes in families related to defense in *Begonia* and other selected genomes.
**Table S13** Statistics and annotations of the contracted gene families in *Begonia*.
**Table S14** The significantly enriched GO terms of biological processes for genes with TEs inserting in introns across four *Begonia* species.
**Table S15** The significantly enriched GO terms of biological processes for genes with TEs inserting in promoter across four *Begonia* species.
**Table S16** Chlorophyll data of the sun‐loving plant *Gerbera hybrida* and four *Begonia* species.
**Table S17** Comparisons of the gene numbers for the light signaling genes in 10 angiosperm genomes.
**Table S18** Comparisons of the gene numbers of the light‐harvesting Chl*a*/*b*‐binding protein (LHCs) family genes in the seven genomes of *Begonia* and other angiosperms.
Please note: Wiley Blackwell are not responsible for the content or functionality of any Supporting Information supplied by the authors. Any queries (other than missing material) should be directed to the *New Phytologist* Central Office.

## Data Availability

All of the raw sequence data including whole‐genome sequencing and transcriptome have been deposited in China National GeneBank Sequence Archive (CNSA) database (https://db.cngb.org/cnsa) under accession number CNP0001056 and National Center for Biotechnology Information (NCBI) under accession number PRJNA791490. The assemblies, gene sequences and annotation data are available at the CNSA database with accession nos. CNA0013973, CNA0013974, CNA0013975 and CNA0013976 for *B. darthvaderiana*, *B. loranthoides*, *B. masoniana* and *B. peltatifolia*, respectively.
