# Supplementary material for: Genomes shed light on the evolution of Begonia, a mega‐diverse genus
Source: New Phytol. 2022 Feb 9;234(1):295–310. doi: 10.1111/nph.17949 (PMC7612470; doi:10.1111/nph.17949)
Supplement: Supplementary file 1 — Fig. S1 Current whole genome shotgun (WGS) sampling of Begonia accessions (78 individuals in 37 sections, as marked in red) on the sectional‐level Begonia phylogeny by Moonlight et al. (2018). Fig. S2 Somatic chromosome counts at metaphase in the four sequenced Begonia species. Fig. S3 K‐mer analyses of the four Begonia species. Fig. S4 Flowchart of sequencing and assembly for the four Begonia species. Fig. S5 Scaffold collinear comparisons between two different species (upper: B. masoniana, bottom: B. peltatifolia) show distinct distributions of different transposon elements. Fig. S6 Distribution of gene density of four Begonia genomes. Fig. S7 Analyses of post‐whole‐genome duplication (WGD) retained gene families in Begonia. Fig. S8 Analysis of post‐WGD retained gene families specific to B. loranthoides. Fig. S9 Analysis of post‐WGD retained gene families specific to B. masoniana. Fig. S10 Analysis of post‐WGD retained gene families specific to B. darthvaderiana. Fig. S11 Analysis of post‐WGD retained gene families specific to B. peltatifolia. Fig. S12 Expansion of gene families in anthocyanin pathway in Begonia. Fig. S13 Gene family expansions and contractions along a dated angiosperm phylogeny of 13 selected species. Fig. S14 Contraction and complete loss of the TNL subgroup of the NBS family in Begonia. Fig. S15 Comparison of TE proportions in 122 shared syntenic blocks across four Begonia species. Fig. S16 Reconstruction of the paleogenome of four sequenced Begonia species. Fig. S17 Number of LTR insertions and genome sizes for 13 angiosperm species. Fig. S18 Number of shared full‐length LTR families across four Begonia species. Fig. S19 Neighbour‐joining trees built from RT domain sequence similarities among different lineage‐specific copies identified in Begonia genomes. Fig. S20 Comparison of nuclear ML tree and abundance clustering of TEs. Fig. S21 The TE landscape surrounding genes in four Begonia species. Fig. S22 Impacts of TE insertions on the structur [file NPH-234-295-s002.pdf]

New Phytologist

***New Phytologist* Supporting Information**

Article title: **Genomes shed light on the evolution of *Begonia*, a mega-diverse genus**

Authors: Lingfei Li, Xiaoli Chen, Dongming Fang, Shanshan Dong, Xing Guo, Na Li, Lucia Campos-Dominguez, Wenguang Wang, Yang Liu, Xiaoan Lang, Yang Peng, Daike Tian, Daniel C. Thomas, Weixue Mu, Min Liu, Chenyu Wu, Ting Yang, Suzhou Zhang, Leilei Yang, Jianfen Yang, Zhong-Jian Liu, Liangsheng Zhang, Xingtan Zhang, Fei Chen, Yuannian Jiao, Yalong Guo, Mark Hughes, Wei Wang, Xiaofei Liu, Chunmei Zhong, Airong Li, Sunil Kumar Sahu, Huanming Yang, Ernest Wu, Joel Sharbrough, Michael Lisby, Xin Liu, Xun Xu, Douglas E. Soltis, Yves Van de Peer, Catherine Kidner, Shouzhou Zhang, Huan Liu

Article acceptance date: 20 December 2021

The following Supporting Information is available for this article:

Methods S1: Supplemental methods

- Cytology
- 10× Genomics ChromiumTM Genome library preparation
- Hi-C library preparation
- SMART library preparation
- Raw data processing and estimation of genome size
- Genome assembly
- Assessment of assembly completeness
- Genome annotation
- Genome evolution
- Principal component analysis (PCA) analysis of TE diversity
- Plastome assembly
- Genetic variation and admixture pattern
- Identification of orthologs of the light regulatory network
- Transcriptome analysis of light/dark-responsive genes

Fig. S1 Current WGS samplings of *Begonia* accessions (78 individuals in 37 sections, as marked in red) on the sectional level *Begonia* phylogeny by Moonlight, *et al* (2018).

Fig. S2 Somatic chromosome counts at metaphase in the four sequenced *Begonia* species.

Fig. S3 K-mer analyses of the four *Begonia* species.

Fig. S4 Flowchart of sequencing and assembly for the four *Begonia* species.

Fig. S5 Scaffold collinear comparisons between two different species (upper: *B. masoniana*, bottom: *B. peltatifolia*) show distinct distribution of different transposon elements.

Fig. S6 Distribution of gene density of four *Begonia* genomes.

Fig. S7 Analyses of post-WGD retained genes families in *Begonia*.

Fig. S8 Analysis of post-WGD retained genes families specific to *B. loranthoides*.

Fig. S9 Analysis of post-WGD retained genes families specific to *B. masoniana*.

Fig. S10 Analysis of post-WGD retained genes families specific to *B. darthvaderiana*.

Fig. S11 Analysis of post-WGD retained gene families specific to *B. peltatifolia*.

Fig. S12 Expansion of gene families in anthocyanin pathway in *Begonia*.

Fig. S13 Gene family expansions and contractions along a dated angiosperm phylogeny of 13 selected species.

Fig. S14 Contraction and complete loss of the TNL subgroup of NBS family in *Begonia*.

Fig. S15 Comparison of TE proportions in 122 shared syntenic blocks across four *Begonia* species.

Fig. S16 Reconstruction of paleo-genome of four sequenced *Begonia* species.

Fig. S17 Number of LTR insertions and genome sizes for 13 angiosperm species.

Fig. S18 Number of shared full length LTR families across four *Begonia* species.

Fig. S19 Neighbor-joining trees built from RT domain sequence similarities among different lineage-specific copies identified in *Begonia* genomes.

Fig. S20 Comparison of nuclear ML tree and abundance clustering of TEs.

Fig. S21 The TE landscape surrounding genes in four *Begonia* species.

Fig. S22 Impacts of TE insertions on the structure of introns and promoters.

Fig. S23 KEGG enrichment of genes with TE insertion either in introns or promoters.

Fig. S24 Expansion of crychromes (CRYs) genes in *Begonia* due to WGD.

Fig. S25 Expansion of Phototropin (PHOT) genes in *Begonia* due to WGD.

Fig. S26 Expansion of Phytochrome (PHY) genes in *Begonia* due to WGD.

Fig. S27 Expansion of UV Resistance Locus 8 (UVR8) genes in *Begonia* due to WGD.

Fig. S28 Schematic diagrams show tandem duplication of LHCB1 genes in *B. masoniana* and *B. darthvaderiana*.

Fig. S29 Nucleotide diversity (π) and population divergence (F_{ST}) across the three major groups of *Begonia*.

Fig. S30 Maximum likelihood tree inferred from concatenated nucleotide sequences of *Begonia* plastid protein coding genes using RAxML.

Fig. S31 Maximum likelihood tree inferred from *Begonia* plastome nucleotide alignment of 156,131 bp using RAxML.

Fig. S32 Maximum likelihood tree inferred from a concatenated dataset of 1,604 nuclear genes using IQtree with individual gene trees mapped.

Fig. S33 Coalescent super tree inferred with ASTRAL-III using 1,604 nuclear single gene trees.

Fig. S34 Coalescent super tree inferred with ASTRAL-III using SNPs in 1,343 nuclear single gene trees.

Fig. S35 Phylonet network results for three geographically delimited *Begonia* clades.

Table S1 Summary of 78 *Begonia* species for whole genome shot gun sequencing. (See separate Excel file)

Table S2 Genome size estimation based on K-mer analysis.

Table S3 Summary of within-genome heterozygosity of the four *Begonia* species.

Table S4 Statistics of genome assemblies.

Table S5 Global statistics of genome assembly and annotation of four *Begonia* species.

Table S6 Statistics of raw data for whole genome sequencing and RNA-seq.

Table S7 Statistics of reads mapping to genome sequences for RNA-seq data from different tissues for four *Begonia* species.

Table S8 Repetitive elements in four *Begonia* genomes. (See separate Excel file)

Table S9 Summary of genomes information across 13 representative angiosperms. (See separate Excel file)

Table S10 Gene Ontology (GO) terms enrichment analysis of the expanded gene

families of *Begonia*.

Table S11 Kyoto Encyclopedia of Genes and Genomes (KEGG) enrichment analysis of the expanded gene families of *Begonia*.

Table S12 Number of genes in families related to defense in *Begonia* and other selected genomes.

Table S13 Statistics and annotations of the contracted gene families in *Begonia*. (See separate Excel file)

Table S14 The significantly enriched GO terms of biological processes for genes with TE inserting in introns across four *Begonia* species.

Table S15 The significantly enriched GO terms of biological processes for genes with TEs inserting in promoter across four *Begonia* species.

Table S16 Chlorophyll data of the sun loving plant *Gerbera hybrida* and four *Begonia* species.

Table S17 Comparisons of the gene numbers for the light signaling genes in 10 angiosperm genomes.

Table S18 Comparisons of the gene numbers of the light-harvesting chlorophyll a/b-binding proteins (LHCs) family genes in the seven genomes of *Begonia* and other angiosperms.

Methods S1 Supplemental methods.

Cytology

Young root tips of the four *Begonia* species were collected at 8:00~10am, immediately pretreated for 4 hr with 0.1% colchicine solution at 4°C, and fixed overnight in ethanol: acetic acid (3:1) at 4°C. The root tips were then dissected on a microscope slide in a drop of 10% hydrochloric acid for 2 min at 60°C. Chromosomes were stained with carbolfuchsin (Solarbio, Beijing, China), and inspected under a microscope (Nikon, Japan). The chromosome numbers were counted and confirmed by at least three cells.

10×Genomics Chromium™ Genome library preparation

High molecular weight (HMW) DNA was isolated using IrysPrep® Plant Tissue DNA Isolation kit following the manufacturer's instructions and assayed by pulsed field gel electrophoresis. For preparation of the Chromium library, the HMW DNA was quantitated and ~1 ng DNA was denatured according to the manufacturer's recommendations (Chromium Genome Chip Kit v1, PN-120229, 10×Genomics, Pleasanton, USA). The denatured DNA was spiked into the reaction master mix, and mixed with gel beads and emulsification oil to generate droplets within a Chromium Genome Chip. Then we finished the rest steps of Chromium library preparation following the manufacturer's protocols, with one modified PCR primer to introduce a 5' phosphorylation site on one amplifying strand. After PCR, the standard circularization step for BGISEQ-500 was carried out and DNA nanoballs (DNB) were prepared as previously described (Drmanac *et al.*, 2010).

Hi-C library preparation

Leaf sample was ground to fine powder in liquid nitrogen and fixed in 1% formaldehyde. After termination of the reaction with glycine, the formaldehyde fixed powder was re-suspended in nuclei isolation buffer (10 mM Tris-HCl pH 8.0 (Sigma, St. Louis, America), 10 mM NaCl (Beyotime, Shanghai, China), 1×PMSF (Sigma, St. Louis, America)). The restriction enzyme (Mbo I) (NEB, Ipswich, America) was added to digest the DNA, followed by the 5' overhang repair (10 mM dCTP, 10 mM dGTP, 10 mM dTTP, (Invitrogen, Waltham, America) 5 U/μl DNA Polymerase I, Large (Klenow) Fragment (NEB, Ipswich, America)) with a biotinylated residue (0.4

mM biotin-14-dATP (Invitrogen, Waltham, America)). The resultant blunt-end fragments were ligated *in situ* (10× NEB T4 DNA ligase buffer (NEB, Ipswich, America), 10% Triton X-100 (Sigma, St. Louis, America), 10 mg/ml BSA (NEB, Ipswich, America), T4 DNA ligase (NEB, Ipswich, America)). Then the isolated DNA was reverse-crosslinked (add 10 mg/ml proteinase K (NEB, Ipswich, America) and 1% SDS (Ambion, Waltham, America) to the tube and incubate at 56°C for overnight) and purified (put the reverse-crosslinked DNA liquid into three tube equally, add 1.5× volumes of AMpure XP (Agencourt, Brea, America) mixture to each tube, vortex and spin down briefly, incubate for 10 min at room temperature, place on the MPS (Invitrogen, Waltham, America) for 5 min, discard supernatant, wash the beads twice with 1 ml of freshly made 70% ethanol (Sinopharm, Shanghai, China), air-dry the beads completely and re-suspend the beads in 30 µl of ddH_2O). The Hi-C library was generated by shearing 20 µg of DNA and capturing the biotin-containing fragments with streptavidin-coated beads using Dynabeads MyOne Streptavidin T1 (Invitrogen, Waltham, America). DNA fragment end repair (10× NEB T4 DNA ligase buffer with 10 mM ATP (NEB, Ipswich, America), 25 mM dNTP mix (Enzymatics, Beverly, America), 10 U/µl NEB T4 PNK (NEB, Ipswich, America), 3 U/µl NEB T4 DNA polymerase I (NEB, Ipswich, America), 5 U/µl NEB DNA polymerase I, Large (Klenow) Fragment (NEB, Ipswich, America)), adenylation (10× NEBuffer 2 (NEB, Ipswich, America), 10 mM dATP (Invitrogen, Waltham, America), 5 U/µl NEB Klenow exo minus (NEB, Ipswich, America)), and adaptor ligation were performed using 10× T4 PNK Reaction Buffer (NEB, Ipswich, America), 100 mM ATP (Fermentas, America), 600 U/ul T4 DNA Ligase (NEB, Ipswich, America), 50% PEG8000 (Rigaku, Tokyo, Japan), 50 µM Ad153 barcode oligo_2B mix (BGI, Shenzhen, China), and followed by PCR (95°C 3 min; [98°C 20 sec., 60°C 15 sec., 72°C 15 sec.] × 8 cycles; 72°C 10 min). After PCR, the standard circularization step required for BGISEQ-500 was carried out and DNB were prepared as previously described (Drmanac *et al.*, 2010).

SMART library preparation

Ten to 15 µg gDNA was sheared using Covaris G-Tubes for 10 min at 1,350 *g* using centrifuge (Beckman, USA). The sheared DNA was concentrated and cleaned using 0.45× Ampure XP beads (Beckman Coulter, Brea, USA). Pacific Biosciences Single

Molecule Real Time (SMRT) bell library was prepared following the protocol (P/N 100-286-000-5) provided by Pacific Biosciences (www.pacb.com) using the SMRTbell Template Prep kit 1.0 (P/N 100-259-100). The resultant SMRTbell libraries were size selected using BluePippin (Sage Science) according to the manufacturer's instructions. The cut-off limit was set to 15-50 kb to select SMRTbell library molecules with an average size of 20 kb or larger. The Pacific Biosciences Binding and Annealing calculator was used to determine the appropriate concentrations for the annealing and binding of the SMRTbell libraries. The libraries were annealed and bound to the P6 DNA polymerase for sequencing using the DNA/Polymerase Binding Kit P6 v2.0 (P/N100-372-700). The only deviation from standard protocol was the extension of binding time from 30 min to 1-3 hr. The bound SMRTbell libraries were loaded onto the SMRT cells using the standard MagBead protocol, and the MagBead Buffer Kit v2.0 (P/N 100—642-800). The standard MagBead sequencing protocol used the DNA Sequencing Kit 4.0 v2 (P/N 100-612-400) with P6/C4 regents. Sequencing data were collected for 6 hr movie times and Stage Start was enabled to capture the longest single reads possible on the PacBio Sequel instrument. Finally, 45.38 and 48.23 Gb data of 3.57 and 4.35 Mb long PacBio reads from *B. masoniana* and *B. darthvaderiana* sequencing libraries were obtained (Table S6), respectively, with more than 84.45% and 80.32% sequencing data longer than 10 kb for the two. With an estimated genome size of ~800 Mb, the data used for contig construction covered 56.73 × and 60.29 × of each genome of *B. masoniana* and *B. darthvaderiana*.

Raw data processing and estimation of genome size

For 10× Genomics and stLFR libraries, raw reads with ambiguous Ns ratio over 5% and low-quality base (quality score less than 10) ratio exceeding 20% were removed using SOAPnuke (v1.5.6) (Chen *et al.*, 2018) with parameters 'filter -l 10 - q 0.2 -n 0.05 -Q 2 --misMatch 1 --matchRatio 0.4'. Duplicated reads that are identical in both ends were also removed by using SOAPnuke (v1.5.6) (Chen *et al.*, 2018) with parameter '-d' to get the final clean data. For the Hi-C library, the raw reads were processed using the HiC-Pro pipeline (Servant *et al.*, 2015).

The k-mer spectrum was built with 10× Genomics clean reads without barcode sequences using the jellyfish (v.1.2.1) (Marcais & Kingsford, 2011). The estimated genome sizes of *Begonia* with this method were 724.13, 805.91, 797.04 and 349.34

Mb for *B. loranthoides*, *B. masoniana*, *B. darthvaderiana*, and *B. peltatifolia*, respectively (Table S2). There were obvious peaks corresponding to k-mer caused by heterozygous sites. The main peak was observed on the half of the x-axis for both genomes of *B. masoniana* and *B. darthvaderiana* (Fig. S3). We also estimated genome heterozygosity ratio via SNP calling, resulting in estimated heterozygosity rates of 0.9%, 0.92%, 0.17%, and 0.24% for *B. masoniana*, *B. darthvaderiana*, *B. loranthoides*, and *B. peltatifolia*, respectively (Table S3), which is consistent with the k-mer estimation.

Considering that the relatively bigger genome size of *B. loranthoides*, *B. masoniana*, *B. darthvaderiana* compared with that of the *B. peltatifolia* may be due to higher repeat content, and k-mers with high frequency were associated with repetitive sequence. K-mer statistics of *B. loranthoides*, *B. masoniana*, *B. darthvaderiana* genomes were compared with that of *B. peltatifolia* (Fig. S3). *B. loranthoides* (63.12%), *B. masoniana* (53.02%), *B. darthvaderiana* (57.13%) genomes represented had about 9%, 19% and 15% k-mer with high-frequency more (two fold peak) than that of the *B. peltatifolia* (71.92%), indicating more repeat content in *B. loranthoides*, *B. masoniana*, *B. darthvaderiana* genome than *B. peltatifolia* genome. Comparison of high-frequent (depth more than 150) k-mer with length of 21, 41, 61 and 81 from reads of *B. loranthoides*, *B. masoniana*, *B. darthvaderiana*, *B. peltatifolia* genome indicated that *B. masoniana* and *B. darthvaderiana* genome had more high-frequent k-mers with length ranged from 21 to 81 than *B. loranthoides* and *B. peltatifolia* genome, suggesting that the lengths of most repeat content in *B. loranthoides* and *B. peltatifolia* genome were shorter than that of the *B. masoniana* and *B. darthvaderiana* genome.

We also compared the sequence or species of same high-frequent k-mer to estimate the specific element content in four *Begonia* genomes. Specific k-mer in *B. masoniana* and *B. darthvaderiana* genome were higher than that in *B. peltatifolia* genome, reflecting the great diversity of high-frequent k-mer sequences or repeats, which might explain the relatively larger genome size of *B. masoniana* and *B. darthvaderiana* than that of *B. peltatifolia*.

Genome assembly

[1] Supernova assembly. For assembly of the 10× Genomics Chromium and stLFR library data, the clean fastq files were converted so as to be read by 10× Genomics Supernova (v.2.1.1) (Weisenfeld *et al.*, 2017) using an in-house script. Reads were then *de novo* assembled using Supernova (v.2.1.1) (Weisenfeld *et al.*, 2017) with default parameters. A minimum fasta record size of 100 bp was specified at the 'mkoutput' stage for outputting the assembly in the 'pseudohap' style. The best resultant assembly accumulated to 716.44, 779.20, 770.49, and 334.09 Mb with a contig N50 of 85.57, 15.26, 13.87, and 99.96 kb and a scaffold N50 of 6.73 Mb, 98.10 kb, 35.29 kb, and 3.20 Mb for *B. loranthoides* (10 ×), *B. masoniana* (stLFR), *B. darthvaderiana* (stLFR), and *B. peltatifolia* (10 ×) (Table S4), respectively. The *B. masoniana* and *B. darthvaderiana* assembly were too fragmented and insufficient compared with the estimated genome size due to high heterozygosity and repeat content.

[2] Canu assembly. *De novo* assemblies of the Pacbio long reads for *B. masoniana* and *B. darthvaderiana* were conducted by Canu (v.0.1) (Koren *et al.*, 2017), which consisted of a four-step process involving: Detect overlaps in high-noise sequences using MHAP; Generate corrected sequence consensus; Trim corrected sequences to exclude some suspicious regions, such as remaining SMRTbell adapter; Assemble trimmed corrected sequences. For *Begonia* genome assembly in Canu (Koren *et al.*, 2017), the following parameters were specified: corOutCoverage=100, genomeSize = 800m. Default parameters were otherwise employed for Canu assembly. After this, two rounds of iterative corrections were performed with PacBio long reads using software Racon (v.1.2.1) (Vaser *et al.*, 2017), and two rounds of corrections with Pilon (v.1.22) (Walker *et al.*, 2014) using 10 × Genomics reads, yielding genome assemblies of 799.39 (contig N50, 436.44 kb) and 771.66 Mb (contig N50, 315.74 kb) for *B. masoniana* and *B. darthvaderiana*, respectively (Table S4).

[3] Comparisons and integration of the assemblies from PacBio and BGISEQ. Upon comparison of BGISEQ-derived contigs (Supernova or stLFR) and PacBio-derived contigs for *B. masoniana* and *B. darthvaderiana*, over 96.7% and 98.4% of the BGISEQ-derived contigs, with over 99% base identity, could be perfectly aligned to the PacBio-derived contigs, suggesting that high-depth BGISEQ data alone could also

produce a high-quality genome. However, the BGISEQ-derived assemblies had fewer complex repeat regions, lacking skewed GC with a lower average depth and long repetitive segmental sequences than the corresponding PacBio assembly. The BGISEQ-derived assembly was merged with the relative Pacbio assembly to produce a hybrid scaffold. BGISEQ sequences were also used to assess the error rate of the integrated assembly and within-genome heterozygosity: high-quality data were aligned to the assembly using BWA-MEM (v. 0.7.10) (Li, 2013), followed by application of GATK (v.3.2-2) (Mckenna *et al.*, 2010) to call variants with a minimum coverage threshold of ten reads, including SNPs and indels. We corrected homozygous mismatches that might affect the base accuracy of the PacBio-derived assembly because of the high error rate (15~20 %) of raw PacBio reads.

Assessment of assembly completeness

[1] DNA and RNA mapping. We assessed the completeness of the genome assembly of four *Begonia* species from two aspects. First, by analysis of the proportions of DNA-seq reads represented in assembly, all the genomic paired-end reads was mapped against the final assembly using BWA-MEM (v. 0.7.10) (Li, 2013), include those from 10× Genomics, stLFR and Hi-C. The high ratios of total mapped reads indicated that most of the sequences were presented in final assembly. Second, by analysis of the proportions of RNA-seq reads represented in assembly, all the short reads from RNA-seq of five tissues (root, stem, flower, scape and leaf) were mapped back to the corresponding assembly using HISAT (Kim *et al.*, 2015). The total mapped reads reflected the representativeness of the expressed genes in the assembly. As a result, similarly high percentages of expressed sequences in five tissues were captured in the assembly.

[2] BUSCO assessment. The genome completeness in terms of expected gene content was quantified using the Benchmarking Universal Single-Copy Ortholog (BUSCO) assessment tool (Simao *et al.*, 2015) for four *Begonia*. Assembly completeness assessments employed BUSCO (v.4.1.2) (Simao *et al.*, 2015) with Augustus (v.3.3) (Stanke *et al.*, 2006), HMMER (v.3.1b2) (Finn *et al.*, 2011), and BLAST+ (v.2.7.1) (Altschul *et al.*, 1990), using both the embryophyta_odb10 and the embryophyta_odb10 BUSCO lineage datasets.

Genome annotation

[1] Gene annotation. We predicted gene models based on homology and *de novo* methods. Results were integrated with GLEAN (Elsik *et al.*, 2014). Homology based gene prediction used the gene models of four species (*Cucumis sativus, Cucurbita moschata*, *Momordica charantia*, and *Prunus mume*). We used TBLASTN to gather a non-redundant set of protein sequences, and then selected the most similar proteins for each candidate protein-coding region based on sequence similarity. Short fragments were connected with a custom script (SOLAR), and Genewise (v.2.0) (Birney *et al.*, 2004) was used to generate the gene structures based on the homology alignments. This generated four gene sets based on homology with four different species. We used Augustus (Stanke *et al.*, 2006), GlimmerHMM (Majoros *et al.*, 2004) and SNAP (Korf, 2004) for *de novo* gene prediction, with parameters trained on 800 intact genes from the homology-based predictions. We chose genes that were predicted by all programs for the final *de novo* gene set.

The four homology-based gene sets and one *de novo* gene set were integrated to generate a consensus gene set with GLEAN (Elsik *et al.*, 2014). We then filtered genes affiliated with repetitive DNA and genes whose coding sequence (CDS) regions contained more than 30% Ns. We used RNA-seq to polish the gene set. After filtering, we mapped reads to the genome with TopHat2 (Kim *et al.*, 2013), and used Stringtie (Pertea *et al.*, 2015) to assemble transcripts. Assembled transcripts were then used to predict open reading frames (ORFs). Transcript-based gene models with intact ORFs that had no overlap with the GLEAN gene set were added. GLEAN gene models were replaced by transcript-based gene models with intact ORFs when there was a discrepancy in length or merging of gene models. Transcripts without intact ORFs were used to extend the incomplete GLEAN gene models to find start and stop codons.

[2] Assessment of gene completeness. The features of predicted genes for four *Begonia* species, including number of genes, exon per gene, average length of mRNA, CDS, exon, intron, were compared with those of other genomes whose gene sets were used for homology-based method (Table S5). Most features of genes predicted in genome of four *Begonia* species were similar to those of other genomes, which

provided the confidence for gene prediction.

Percentages of RNA-seq reads that could be mapped to CDS of predicted genes can be used to assess the comprehension and completeness of the annotation of protein-coding genes. Data mapping revealed ~30% of unmapped RNA-seq reads, which could be attributed to two causations. First, they come from un-translated part of expressed genes; Second, they came from gene missed in the gene annotation. Considering good presentation of BUSCOs in genome assembly and gene set (Table S4), we inferred that they came from genes associated with *Begonia* specific features not shared by genes used in homology-based annotation.

Annotated gene set completeness was quantified using the BUSCO (Simao *et al.*, 2015) for four *Begonia* species (Table S4). Gene sets were first filtered to select the single longest protein sequence for any genes with annotated alternative transcripts. Gene set completeness assessments employed BUSCO (v.4.1.2) (Simao *et al.*, 2015) with HMMER (v.3.1b2) (Finn *et al.*, 2011), and BLAST+ (v.2.7.1) (Altschul *et al.*, 1990), using both the embryophyta_odb9 and the embryophyta_odb10 BUSCO lineage datasets.

[3] Function of proteins coded by predicted genes. Annotation of the predicted genes of four *Begonia* species were performed by aligning their sequences against a number of protein sequence databases, including InterPro (55.0) (Hunter *et al.*, 2009), Gene Ontology (Ashburner *et al.*, 2000), KEGG (v.89.1) (Ogata *et al.*, 1999), Swiss-Prot (release- 2017_09) (Boeckmann *et al.*, 2003), TrEMBL (release- 2017_09) (Boeckmann *et al.*, 2003) and NR (20170924). First, for the predicted protein-coding gene set for four *Begonia* species, each translated amino acid sequence was assessed for conserved protein domains in the Gene3D (Yeats *et al.*, 2007), HAMAP (Lima *et al.*, 2009), Pfam (Finn *et al.*, 2007), PIRSF (Wu *et al.*, 2004), PRINTS (Attwood *et al.*, 2003), ProDom (Corpet *et al.*, 2000), SMART (Letunic *et al.*, 2009), SUPERFAMILY (Wilson *et al.*, 2007), and TIGRFAM (Selengut *et al.*, 2007) databases using InterProScan (Quevillon *et al.*, 2005). The Gene Ontology IDs for each gene were obtained from the corresponding InterPro entries. Second, amino acid sequences were subjected to BLASTP v2.2.26 (e-value < 1e-5) using the following protein databases: Swiss-Prot (release- 2017_09) (Boeckmann *et al.*, 2003), TrEMBL (release- 2017_09) (Boeckmann *et al.*, 2003), Kyoto Encyclopaedia of Genes and Genomes (KEGG, v.

89.1) (Ogata *et al.*, 1999) and NCBI protein NR.

[4] Repeats in genome of *Begonia*. Repeat content is a general contributor for variation in genome size. Repeat sequences in the four *Begonia* genomes were identified as following: Tandem repeats were searched across the genome using the software Tandem Repeats Finder (v.4.07) (Benson, 1999); transposable elements (TEs) were predicted using a combination of homology-based comparisons with RepeatMasker (v.4.0.5) (Tarailo-Graovac & Chen, 2009) and RepeatProteinMask (Tarailo-Graovac & Chen, 2009), and *de novo* approaches with GT ltrharvest (Ellinghaus *et al.*, 2008), LTR_FINDER (v.1.0.6) (Xu & Wang, 2007), and RepeatScout (v.1.0.5) (Price *et al.*, 2005). RepeatMasker (Tarailo-Graovac & Chen, 2009) was employed for DNA-level identification using a general library (RepBase20.04). At the protein level, RepeatProteinMask (Tarailo-Graovac & Chen, 2009), as implemented in RepeatMaske (Tarailo-Graovac & Chen, 2009) package, was employed for a WuBlastX search against the TE protein database. GT ltrharvest (Ellinghaus *et al.*, 2008) and LTR_FINDER (Xu & Wang, 2007) searched the whole genome for a characteristic structure of the full-length long terminal repeat retrotransposons (LTRs). RepeatScout (Price *et al.*, 2005) built consensus sequences using fit-preferred alignment score. Contamination and multi-copy genes in the library were filtered first. RepeatMasker (Tarailo-Graovac & Chen, 2009) was used to predict the TEs with the library generated by RepeatScout. Finally, RepeatScout (Price *et al.*, 2005) was performed again to find homologs in the genome and categorize the identified repeats.

We also estimated TE insertion times with full-length LTR retrotransposons. The 5’- and 3’- LTR sequences of the retrotransposons were aligned and used to calculate the *K*-value (the average number of substitutions per aligned site) using the EMBOSS (v.6.5.7.0) (Rice *et al.*, 2000) package Distmat. The insertion times (T) were calculated using the formula: $T = K / (2 * r)$, where r represents the average substitution rate, which is $1.3 * 10^{-8}$ substitutions per synonymous site per year. Among these elements, long terminal repeats (LTRs) were the most dominant type, accounting for approximately 67.44% and 63.45% of *B. masoniana* and *B. darthvaderiana* genome. TE insertion time calculations revealed a burst of LTR activity during the last 10 MYA (Fig. 3b), which is younger than the divergence of

four *Begonia* species (10-20 MYA), indicating that these LTRs were inserted into the genome after the divergences of the four *Begonia* species.

The annotation of transposon protein domains was further refined using DANTE-Protein Domain Finder, a new tool available at the RepeatExplorer server (Novak *et al.*, 2013), which employs BLAST searches against custom database of transposon protein domains. The hits were filtered to cover at least 80% of the reference sequence, with minimum identity of 35% and minimum similarity of 45%, allowing for max three interruptions (frame shifts or stop codons). To estimate the relative divergence times of the transposons, the ultrametric trees were calculated using PATHd8 (Britton *et al.*, 2007) program and relative branching times were extracted from the trees using R package Ape (Paradis *et al.*, 2004).

Genome evolution

[1] Orthology delineation. Comparative genomic analysis was used to examine the rate of protein evolution and the conservation of gene repertoires among orthologs in the thirteen genomes. First, we aligned all-to-all proteins using BLASTP with an e-value cut-off of 1e-5; Genes were then clustered using OrthoMCL (v.1.4) (Li *et al.*, 2003) with a Markov inflation index of 1.5 and a maximum e-value of 1e-5. On this basis, all ortholog groups (OGs) were ascertained for the thirteen reference genomes, and that, genes belonging to all or individual *Begonia* specific gene families and/or un-clustered genes were identified. Furthermore, based on pair-wise BLASTP alignment of *B. loranthoides* and three other *Begonia* species (*B. masoniana*, *B. darthvaderiana*, and *B. peltatifolia*) with an e-value of 1e-5, we used the reciprocal best method to identify orthologous genes among four *Begonia* species, called reciprocal best ortholog gene pairs, which were high similar on amino acid level.

[2] Phylogenomic analysis. We performed phylogenomic analysis of the thirteen selected taxa with sequenced genomes by using one-to-one single-copy orthologous genes. OrthoMCL (Li *et al.*, 2003) clustered a total of 193 single-copy gene families, which were individually aligned with MAFFT (Katoh *et al.*, 2005) and then subjected to phylogenetic analyses using Mrbayes (Ronquist & Huelsenbeck, 2003) with *Vitis vinifera* as outgroup. Phylogenomic reconstruction recovered a robust phylogenetic tree with all the relationships among the four *Begonia* species receiving full (1.00)

posterior probability support. We dated the phylogenetic tree with McMcTree as implemented in PAML (Yang, 2007), using two node calibrations: the split of Vitis-Cucurbitales (105-115 MYA), and the divergence of *Momordica charantia* from the rest of Cucurbitales (46-60 MYA). The dating result suggested the divergences of the four *Begonia* species occurred between 9.8-21.8 MYA.

Based on the dated phylogenetic tree of the 13 species, the expansion and contraction of the gene clusters were determined by CAFÉ (v.2.1) (De Bie *et al.*, 2006) on the basis of changes in gene family size in the generated phylogenetic history. This method models gene family evolution as a stochastic birth and death process, where genes were gained and lost independently along each branch of the phylogenetic tree. The result describes the rate of change as the probability that a gene family either expands (via gene gain) or contracts (via gene loss) per gene per million years, and can be estimated independently for all branches (Fig. S13).

[3] Evolutionary rate analysis. Single copy orthologs were extracted from OGs identified above. Peptide alignments were obtained by running GUIDANCE2 (Sela *et al.*, 2015) with the PRANK (Löytynoja, 2014) aligner and species tree were generated for each orthogroup. Low scoring residues were masked to N using GUIDANCE2 to mask poor quality regions of each alignment. PAL2NAL (Suyama *et al.*, 2006) was used to back-translate aligned peptide sequences to CDS and format alignments for PAML. PAML (Yang, 2007) was run to evaluate the likelihood of multiple hypothesized branch models of dN/dS relative to two null models with trees and parameters as follows: (((((((((2,6),4),3),7),1 #1),8),5),9),10).

[4] Functional enrichment tests. For a given gene list, such as the *B. loranthoides* specific genes or conserved genes between *B. loranthoides* and other three *Begonia* species (*B. masoniana*, *B. darthvaderiana* and *B. peltatifolia*) that were used for GO enrichment analysis: the given gene list was carried out based on the algorithm implemented in GOstat, with the whole annotated gene set as the background. GOstat tests for GO terms represented by significantly more genes in a given gene set using chi-square test. Fisher's exact test is used when expected counts are below 5, which makes the chi-square test inaccurate. The computed p-value was then adjusted for multiple tests by specifying a false discovery rate (q-value < 0.05) using the

Benjamini-Hochberg method (Benjamini & Hochberg, 2000). Similar methods were also used for KEGG enrichment analysis.

[5] Gene collinearity. The syntenic blocks between two species were defined by MCscan (v. 0.8) (Tang *et al.*, 2008) based on core-orthologous gene sets identified using BLASTP (e-value <= 1e - 5 ; number of genes required to call synteny >= 5). Genes were then classified as collinear or non-collinear according to whether they have a homologous gene in the orthologous regions. If a homologous gene was not detected in the syntenic region of the target genome, we would search for homologous DNA sequences of the candidate gene in this region and syntenic status would be assigned 'without synteny status' for this gene when sequence remnants was detected, which means the orthologous gene was probably mis-annotated and the synteny status of this gene is not sure. To minimize the influence of sequence gaps on synteny analysis, we manually inspected the gap-containing genes and gap-flanking genes to confirm their syntney status and incorporate the result into synteny analysis. We also used *C. sativus* as outgroup to filter these candidate non-collinear genes that were collinear with outgroup.

Whole-genome duplication (WGD) is an important source for functional differentiation of genes. The sequence divergence for all possible pairs of paralogs within each collinear block was estimated based on *Ks*. Protein sequences were aligned using MAFFT (Katoh *et al.*, 2005) and converted into codon-aligned nucleotides using the Bioruby-alignment package. *Ks* values were calculated through maximum likelihood estimation (MLE) using the 'codeml' 101 and 'yn00' 102 programs in the PAML (Yang, 2007) package and using the following parameters: runmode = -2, set-type = 1 (codon sequences), alpha fixed to 0, codonFreq = 2 (F2X4). For all *Ks* distribution histograms, the x-axes were drawn with non-transformed *Ks* values. The range of values, 0-3 was binned into 75 interval-bins by step 0.04. We used the nucleotide substitution rate of 7×10^{-9} per site per year (*r*) from synonymous sites, time of divergence was calculated with the formula T = *Ks* / *2r*, where *Ks* is the number of substitutions per base between subgenomes and r is the rate of substitution. The bimodal nature of the *Begonia Ks* histogram was: the WGD event shared by all four *Begonia* species estimated to have occurred ~35 MYA (mean: 35 MYA, std dev: 8 MYA, *Ks* = 0.50 ± 0.11) and, the whole-genome triplication event

(γ) common to all core Eudicots occurred ~118 MYA (mean: 118 MYA, std dev: 2 MYA, *Ks* = 1.65 ± 0.29) (Fig. 2c).

[6] Reconstruction of the *Begonia* paleo-genome. An evolutionary scenario was obtained following the method described by Pont *et al* (Pont *et al.*, 2019), based on synteny relationships identified between between *B. peltatifolia* and *B. masoniana*, *B. peltatifolia* and *B. darthvaderiana*, and *B. peltatifolia* and *B. loranthoides*. Briefly, the first step consisted of aligning the investigated genomes to define conserved/duplicated gene pairs on the basis of alignment parameters (CIP for Cumulative Identity Percentage and CALP Cumulative Alignment Length Percentage). The second step consisted of clustering or chaining groups of conserved genes into synteny blocks (excluding blocks with less than 5 genes) corresponding to independent sets of blocks sharing orthologous relationships in modern species. In the third step, conserved gene pairs or conserved groups of gene-to- gene adjacencies defining identical chromosome-to-chromosome relationships between all the extant genomes were merged into conserved ancestral regions (CARs). CARs were then merged into proto- chromosomes based on partial synteny observed between a subset (not all) of the investigated species. The ancestral karyotype can be considered as a 'median' or 'intermediate' genome consisting of proto- chromosomes defining a clean reference gene order common to the extant species investigated. From the reconstructed ancestral karyotype an evolutionary scenario was then inferred taking into account the fewest number of genomic rearrangements (including inversions, deletions, fusions, fissions, translocations) that may have operated between the inferred ancestors and the modern genomes (Fig. S16).

Principal component analysis (PCA) analysis of TE diversity

Low-quality sequences FASTQ with bases having quality below 20 on Phred 33 scale, adapter trimming, and duplicates removal was performed using SOAPnuke (v.1.5.6) program. The reads with length less than 120 bp were discarded and only the paired reads were used for subsequent analyses. The whole filtered reads were mapping to custom transposon protein domains database (Viridiplantae_v3.0_pdb) with blastx. The best hits were filtered to cover at least 90% of the reference sequence, with minimum identity of 30%. After this, the counts were normalized by dividing the

number of counts on a specific domain by the total number of counts on all TE domains and by the total number of occurrences of each domain per million, and then counts were transformed to log10 scale. PCA was conducted across the TE domain with at least 10% of the individuals happened with FactoMineR package, and results were visualized with factoextra package.

Plastome assembly

The raw WGS data were trimmed and filtered for adaptors, low quality reads, undersized inserts, and duplicate reads using Trimmomatic (Bolger *et al.*, 2014). The resultant clean reads were used for *de novo* plastome assembly with Novoplasty (Dierckxsens *et al.*, 2017) using the seed sequence of *rbcL*, and the reference genome sequence of the *B. masoniana* plastome extracted from the genome assembly of *B. masoniana* based on PacBio long reads data. All the newly assembled 78 *Begonia* plastid genomes were annotated by transferring the annotations from the published plastome of *Cucumis sativus* from the closely related family Cucurbitaceae to *Begonia* sequences following MAFFT (Katoh *et al.*, 2005) alignment in Geneious (v.10.0.2) (Biomatters, New Zealand).

Genetic variation and admixture pattern

[1] Phylogenetic reconstruction

Phylogenetic trees of nuclear and chloroplast DNA sequences were constructed based on SNPs within regions of single-copy genes. The filtered SNPs were converted to phylip format. The maximum likelihood (ML) trees were constructed using IQ-Tree (Nguyen *et al.*, 2015) with self-estimated best substitution model and ultra-fast bootstrap replicates of 5,000. The best maximum likelihood tree was used as a starting tree to estimate species divergence time using MCMC Tree as implemented in PAML (Yang, 2007). One calibration point of the *Begonia* crown group (24 +3.57 MYA with a normal distribution) was defined following Moonlight *et al* (Moonlight *et al.*, 2018).

To validate the phylogenetic reconstructions of plastid and nuclear SNP dataset, we also produced a large dataset of 1,604 nuclear single copy genes using the software Hybpiper (Johnson *et al.*, 2016) using the reference protein sequences of 4,000 single copy genes extracted from four newly generated *Begonia* genomes. Individual genes were manually checked for orthology, and filtered based on taxa

occurrences (> 50%), aligned with MAFFT (Katoh *et al.*, 2005), and trimmed with GBLOCKS (Talavera & Castresana, 2007). Supermatrix and supertree method were used to infer the nuclear phylogeny using RAxML (v.7.2.3) (Stamatakis, 2006) and ASTRAL III (Mirarab *et al.*, 2014), respectively.

[2] Admixture analysis

Ancestral population stratification among all the *Begonia* accessions was inferred using Admixture (Alexander *et al.*, 2009) software. We evaluated the ancestral population sizes of *K*=1-20 and selected the population with the smallest cross-validation error (*K*=3 in this case). The parameter standard errors were estimated using bootstrapping (bootstrap=200) when doing the admixture analyses.

[3] Principal components analysis and diversity statistics

We used PLINK (v.2) (Chang *et al.*, 2015), GCTA (v.1.93) (Yang *et al.*, 2011), and VCFtools (v.0.1.16) (Danecek *et al.*, 2011) for the calculation of principal components and other population divergence statistics.

[4] ABBA-BABA analysis

To detect the introgression among *Begonia* species, we calculated the Patterson's D statistic using the program Dsuite (Malinsky *et al.*, 2020). D-statistic is widely used to examine site patterns (also known as ABBA/ABAB patterns) in genome alignments for a specified four-taxon tree. Given four taxa with the relationship of "[(P1,P2),P3],O", a D-statistic significantly differed from zero indicate introgression between population P1 and P3 (negative D value) or between P2 and P3 (positive D value).

Identification of orthologs of the light regulatory network

The representative *Arabidopsis* proteins (CRYs, AT4G08920, AT1G04400; CUL4, AT5G46210; COP1, AT2G32950; UVR8, AT5G63860; SPA, AT1G53090, AT3G15354, AT2G46340, AT4G11110; HY5, AT5G11260, AT3G17609; PhyA-E, AT1G09570, AT2G18790, AT5G35840, AT4G16250, AT4G18130; EIN3 and EIL1, AT2G25490 and AT5G25350; FHY3 and FAR1, AT3G22170 and AT4G15090; YUC, AT4G32540, AT1G04180, AT2G33230, AT1G04610, AT5G25620, AT4G28720,

AT5G43890, AT1G48910; HB2 and HB4, AT4G16780, AT2G44910; EBF, AT2G25490 and AT5G25350; PHOT1-2, AT3G45780 and AT5G58140) in the light regulatory network were used as templates to perform BLASTP searches against the protein sequences of *Begonia* and selected angiosperm genomes. Sequences with an e-value below 1e-10 and reference sequence coverage over 50% were extracted and annotated with InterProScan (Quevillon *et al.*, 2005). Sequences that were assigned to the corresponding gene family were identified as candidate orthologs. All the candidate orthologs were subjected to phylogenetic reconstruction using MAFFT (Katoh *et al.*, 2005) and PhyML (v3.1) (Guindon & Gascuel, 2003) to further remove the false positives.

As all the PIF genes identified are members of the bHLH subfamily 15, hence we take the following steps to characterize PIF genes as described by Chang *et al* (Han *et al.*, 2019). First, HLH domain (PF00010) was used as templates to perform HMMER (Finn *et al.*, 2011) searches with an e-value cutoff of 1e-2. Then, all the hits were extracted to reconstruct the phylogenetic tree using PhyML (v3.1) (Guindon & Gascuel, 2003). After that, those PIFs that occurred in a monophyletic assemblage with *Arabidopsis* homologs and contained APB motif (ELxxxxGQ) were selected as candidate PIF orthologs (Han *et al.*, 2019).

Sequences that assigned the “crytochrome/DNA photolyase class 1” annotation with conserved PF03441, PF00875, PF12546 domains were identified as CRY orthologs. The sequences annotated with cullin-4B and aligned with PF00888 were extracted as candidate CUL orthologs. Sequences with the InterProScan annotation of “Ultraviolet-B receptor UVR8” and alignment domain corresponding to HMM profile (PF00415) were identified as candidate UVR8 orthologs. The sequences annotated with cullin-4B and aligned with PF00888 were extracted as candidate CUL orthologs.

Transcriptome analysis of light/dark-responsive genes

For light/dark treatment experiments, seedling of B. masoniana were obtained from tissue culture, after growth in artificial climate chamber for one month under condition of 24°C, 16 h of light/8 h of dark, light intensity 45 μmol·m-2·sec-1, the plants were transferred to continuous white light or darkness for 3 days before transfer to the opposite light condition for 1 and 3 days. Leaf samples were frozen in liquid nitrogen and stored at -80°C until RNA isolation for RNA-seq.

Total RNAs were extracted using TIANGEN RNA extraction kit (Tiangen Biotech Co., Ltd., Beijing, China). Total RNA (1.5 μg) from each sample was prepared, and libraries were generated using NEBNext® Ultra™ RNA Library Prep Kit for Illumina® (NEB, USA). Subsequently, the libraries were sequenced on an Illumina HiSeq platform (Novogene Co., Ltd., Beijing, China). Three biological replicates were used for each sample. Gene expression levels were calculated as fragments-per-kilobase-of-transcript-per-million-fragments-mapped (FPKM).

References

Alexander DH, Novembre J, Lange K. 2009. Fast model-based estimation of ancestry in unrelated individuals. *Genome Research* **19**: 1655-1664.

Altschul SF, Gish W, Miller W, Myers EW, Lipman DJ. 1990. Basic local alignment search tool. *Journal of Molecular Biology* **215**: 403-410.

Ashburner M, Ball CA, Blake JA, Botstein D, Butler H, Cherry JM, Davis AP, Dolinski K, Dwight SS, Eppig JT, et al. 2000. Gene ontology: tool for the unification of biology. *Nature Genetics* **25**: 25-29.

Attwood TK, Bradley P, Flower DR, Gaulton A, Maudling N, Mitchell AL, Uddin A. 2003. PRINTS and its automatic supplement, prePRINTS. *Nucleic Acids Research* **31**: 400-402.

Benjamini Y, Hochberg Y. 2000. On the adaptive control of the false discovery rate in multiple testing with independent statistics. *Journal of Educational and Behavioral Statistics* **25**: 60-83.

Benson G. 1999. Tandem repeats finder: a program to analyze DNA sequences. *Nucleic Acids Research* **27**: 573-580.

Birney E, Clamp M, Durbin R. 2004. GeneWise and Genomewise. *Genome Research* **14**: 988-995.

Boeckmann B, Bairoch A, Apweiler R, Blatter MC, Estreicher A, Gasteiger E, Martin MJ, Michoud K, O'Donovan C, Phan I, et al. 2003. The SWISS-PROT protein knowledgebase and its supplement TrEMBL in 2003. *Nucleic Acids Research* **31**: 365-370.

Bolger AM, Lohse M, Usadel B. 2014. Trimmomatic: a flexible trimmer for Illumina sequence data. *Bioinformatics* **30**: 2114-2120.

Britton T, Anderson CL, Jacquet D, Lundqvist S, Bremer K. 2007. Estimating divergence times in large phylogenetic trees. *Systematic Biology* **56**: 741-752.

Chang CC, Chow CC, Tellier LC, Vattikuti S, Purcell SM, Lee JJ. 2015. Second-generation PLINK: rising to the challenge of larger and richer datasets. *Gigascience* **4**: 7.

Chen Y, Chen Y, Shi C, Huang Z, Zhang Y, Li S, Li Y, Ye J, Yu C, Li Z, et al. 2018. SOAPnuke: a MapReduce acceleration-supported software for integrated quality control and preprocessing of high-throughput sequencing data. *Gigascience* **7**: 1-6.

Corpet F, Servant F, Gouzy J, Kahn D. 2000. ProDom and ProDom-CG: tools for protein domain analysis and whole genome comparisons. *Nucleic Acids Research* **28**: 267-269.

Danecek P, Auton A, Abecasis G, Albers CA, Banks E, DePristo MA, Handsaker RE, Lunter G, Marth GT, Sherry ST, et al. 2011. The variant call format and VCFtools. *Bioinformatics* **27**: 2156-2158.

De Bie T, Cristianini N, Demuth JP, Hahn MW. 2006. CAFE: a computational tool for the study of gene family evolution. *Bioinformatics* **22**: 1269-1271.

Dierckxsens N, Mardulyn P, Smits G. 2017. NOVOPlasty: *de novo* assembly of organelle genomes from whole genome data. *Nucleic Acids Research* **45**: e18.

Drmanac R, Sparks AB, Callow MJ, Halpern AL, Burns NL, Kermani BG, Carnevali P, Nazarenko I, Nilsen GB, Yeung G, et al. 2010. Human genome sequencing using unchained base reads on self-assembling DNA nanoarrays. *Science* **327**: 78-81.

Ellinghaus D, Kurtz S, Willhoeft U. 2008. LTRharvest, an efficient and flexible software for de novo detection of LTR retrotransposons. *BMC Bioinformatics* **9**: 18.

Elsik CG, Worley KC, Bennett AK, Beye M, Camara F, Childers CP, de Graaf DC, Debyser G, Deng J, Devreese B, et al. 2014. Finding the missing honey bee genes: lessons learned from a genome upgrade. *BMC Genomics* **15**: 86.

Finn RD, Clements J, Eddy SR. 2011. HMMER web server: interactive sequence similarity searching. *Nucleic Acids Research* **39**: W29-37.

Finn RD, Tate J, Mistry J, Coggill PC, Sammut SJ, Hotz HR, Bateman A. 2007. The Pfam protein families database. *Nucleic Acids Research* **36**: D281-D288.

Guindon S, Gascuel O. 2003. A simple, fast, and accurate algorithm to estimate large phylogenies by maximum likelihood. *Systematic Biology* **52**: 696-704.

Han X, Chang X, Zhang Z, Chen H, He H, Zhong B, Deng XW. 2019. Origin and evolution of core components responsible for monitoring light environment changes during plant terrestrialization. *Molecular Plant* **12**: 847-862.

Hunter S, Apweiler R, Attwood TK, Bairoch A, Bateman A, Binns D, Bork P, Das U, Daugherty L, Duquenne L, et al. 2009. InterPro: the integrative protein signature database. *Nucleic Acids Research* **37**: D211-215.

Johnson MG, Gardner EM, Liu Y, Medina R, Goffinet B, Shaw AJ, Zerega NJ, Wickett NJ. 2016. HybPiper: Extracting coding sequence and introns for phylogenetics from high-throughput sequencing reads using target enrichment. *Applications in Plant*

Sciences **4**: apps. 1600016.

Katoh K, Kuma K, Toh H, Miyata T. 2005. MAFFT version 5: improvement in accuracy of multiple sequence alignment. *Nucleic Acids Research* **33**: 511-518.

Kim D, Langmead B, Salzberg SL. 2015. HISAT: a fast spliced aligner with low memory requirements. *Nature Methods* **12**: 357-360.

Kim D, Pertea G, Trapnell C, Pimentel H, Kelley R, Salzberg SL. 2013. TopHat2: accurate alignment of transcriptomes in the presence of insertions, deletions and gene fusions. *Genome Biology* **14**: R36.

Koren S, Walenz BP, Berlin K, Miller JR, Bergman NH, Phillippy AM. 2017. Canu: scalable and accurate long-read assembly via adaptive k-mer weighting and repeat separation. *Genome Research* **27**: 722-736.

Korf I. 2004. Gene finding in novel genomes. *BMC Bioinformatics* **5**: 59.

Löytynoja A 2014. Phylogeny-aware alignment with PRANK.In Russell DJ. *Multiple Sequence Alignment Methods*. Totowa (NJ): Humana Press. 155-170.

Letunic I, Doerks T, Bork P. 2009. SMART 6: recent updates and new developments. *Nucleic Acids Research* **37**: D229-D232.

Li H. 2013. Aligning sequence reads, clone sequences and assembly contigs with BWA-MEM. *arXiv*: 1303.3997.

Li L, Stoeckert CJ, Jr., Roos DS. 2003. OrthoMCL: identification of ortholog groups for eukaryotic genomes. *Genome Research* **13**: 2178-2189.

Lima T, Auchincloss AH, Coudert E, Keller G, Michoud K, Rivoire C, al. e, Phan I. 2009. HAMAP: a database of completely sequenced microbial proteome sets and manually curated microbial protein families in UniProtKB/Swiss-Prot. *Nucleic Acids Research* **37**: D471-D478.

Majoros WH, Pertea M, Salzberg SL. 2004. TigrScan and GlimmerHMM: two open source ab initio eukaryotic gene-finders. *Bioinformatics* **20**: 2878-2879.

Malinsky M, Matschiner M, Svardal H. 2020. Dsuite - fast D-statistics and related admixture evidence from VCF files. *bioRxiv*: 634477.

Marcais G, Kingsford C. 2011. A fast, lock-free approach for efficient parallel counting of occurrences of k-mers. *Bioinformatics* **27**: 764-770.

Mckenna A, Hanna M, Banks E, Sivachenko A, Cibulskis K, Kernytsky A. 2010. The Genome Analysis Toolkit: A MapReduce framework for analyzing next-generation DNA sequencing data. *Genome Research* **20**: 1297-1303.

Mirarab S, Reaz R, Bayzid MS, Zimmermann T, Swenson MS, Warnow T. 2014. ASTRAL: genome-scale coalescent-based species tree estimation. *Bioinformatics* **30**: i541-548.

Moonlight PW, Ardi WH, Padilla LA, Chung K-F, Fuller D, Girmansyah D, Hollands R, Jara-Muñoz A, Kiew R, Leong W-C, et al. 2018. Dividing and conquering the fastest–growing genus: Towards a natural sectional classification of the mega–diverse genus *Begonia* (Begoniaceae). *Taxon* **67**: 267-323.

Nguyen LT, Schmidt HA, von Haeseler A, Minh BQ. 2015. IQ-TREE: a fast and effective stochastic algorithm for estimating maximum-likelihood phylogenies. *Molecular Biology and Evolution* **32**: 268-274.

Novak P, Neumann P, Pech J, Steinhaisl J, Macas J. 2013. RepeatExplorer: a Galaxy-based web server for genome-wide characterization of eukaryotic repetitive elements from next-generation sequence reads. *Bioinformatics* **29**: 792-793.

Ogata H, Goto S, Sato K, Fujibuchi W, Bono H, Kanehisa M. 1999. KEGG: Kyoto Encyclopedia of Genes and Genomes. *Nucleic Acids Research* **27**: 29-34.

Paradis E, Claude J, Strimmer K. 2004. APE: Analyses of phylogenetics and evolution in R language. *Bioinformatics* **20**: 289-290.

Pertea M, Pertea GM, Antonescu CM, Chang TC, Mendell JT, Salzberg SL. 2015. StringTie enables improved reconstruction of a transcriptome from RNA-seq reads. *Nature Biotechnology* **33**: 290.

Pont C, Wagner S, Kremer A, Orlando L, Plomion C, Salse J. 2019. Paleogenomics: reconstruction of plant evolutionary trajectories from modern and ancient DNA. *Genome Biology* **20**: 29.

Price AL, Jones NC, Pevzner PA. 2005. *De novo* identification of repeat families in large genomes. *Bioinformatics* **21** i351-358.

Quevillon E, Silventoinen V, Pillai S, Harte N, Mulder N, Apweiler R, Lopez R. 2005. InterProScan: protein domains identifier. *Nucleic Acids Research* **33**: W116-W120.

Rice P, Longden I, Bleasby A. 2000. EMBOSS: the European molecular biology open software suite. *Trends in Genetics* **16**: 276-277.

Ronquist F, Huelsenbeck JP. 2003. MrBayes 3: Bayesian phylogenetic inference under mixed models. *Bioinformatics* **19**: 1572-1574.

Sela I, Ashkenazy H, Katoh K, Pupko T. 2015. GUIDANCE2: accurate detection of unreliable alignment regions accounting for the uncertainty of multiple parameters.

Nucleic Acids Research **43**: W7-14.

Selengut JD, Haft DH, Davidsen T, Ganapathy A, Gwinn-Giglio M, al. e. 2007. TIGRFAMs and Genome Properties: tools for the assignment of molecular function and biological process in prokaryotic genomes. *Nucleic Acids Research* **35**: D260-D264.

Servant N, Varoquaux N, Lajoie BR, Viara E, Chen CJ, Vert JP, Heard E, Dekker J, Barillot E. 2015. HiC-Pro: an optimized and flexible pipeline for Hi-C data processing. *Genome Biology* **16**: 259.

Simao FA, Waterhouse RM, Ioannidis P, Kriventseva EV, Zdobnov EM. 2015. BUSCO: assessing genome assembly and annotation completeness with single-copy orthologs. *Bioinformatics* **31**: 3210-3212.

Stamatakis A. 2006. RAxML-VI-HPC: maximum likelihood-based phylogenetic analyses with thousands of taxa and mixed models. *Bioinformatics* **22**: 2688-2690.

Stanke M, Keller O, Gunduz I, Hayes A, Waack S, Morgenstern B. 2006. AUGUSTUS: *ab initio* prediction of alternative transcripts. *Nucleic Acids Research* **34**: W435-W439.

Suyama M, Torrents D, Bork P. 2006. PAL2NAL: robust conversion of protein sequence alignments into the corresponding codon alignments. *Nucleic Acids Research* **34**: W609-612.

Talavera G, Castresana J. 2007. Improvement of phylogenies after removing divergent and ambiguously aligned blocks from protein sequence alignments. *Systematic Biology* **56**: 564-577.

Tang H, Wang X, Bowers JE, Ming R, Alam M, Paterson AH. 2008. Unraveling ancient hexaploidy through multiply-aligned angiosperm gene maps. *Genome Research* **18**: 1944-1954.

Tarailo-Graovac M, Chen N. 2009. Using RepeatMasker to identify repetitive elements in genomic sequences. *Current Protocols in Bioinformatics* **Chapter 4**: 4.10.11-14.10.14.

Vaser R, Sovic I, Nagarajan N, Sikic M. 2017. Fast and accurate *de novo* genome assembly from long uncorrected reads. *Genome Research* **27**: 737-746.

Walker BJ, Abeel T, Shea T, Priest M, Abouelliel A, S. S, Cuomo CA, Zeng Q, Wortman J, Young SK, et al. 2014. Pilon: an integrated tool for comprehensive microbial variant detection and genome assembly improvement. *PLoS ONE* **9**: e112963.

Weisenfeld NI, Kumar V, Shah P, Church DM, Jaffe DB. 2017. Direct determination of diploid genome sequences. *Genome Research* **27**: 757-767.

Wilson D, Madera M, Vogel C, Chothia C, Gough J. 2007. The SUPERFAMILY database in 2007: families and functions. *Nucleic Acids Research* **35**: D308-D313.

Wu CH, Nikolskaya A, Huang H, Yeh LSL, Natale DA, Vinayaka CR, Ledley RS. 2004. PIRSF: family classification system at the Protein Information Resource. *Nucleic Acids Research* **32**: D112-D114.

Xu Z, Wang H. 2007. LTR_FINDER: an efficient tool for the prediction of full-length LTR retrotransposons. *Nucleic Acids Research* **35**: W265-268.

Yang J, Lee SH, Goddard ME, Visscher PM. 2011. GCTA: a tool for genome-wide complex trait analysis. *American Journal of Human Genetics* **88**: 76-82.

Yang Z. 2007. PAML4: phylogenetic analysis by maximum likelihood. *Molecular Biology and Evolution* **24**: 1586-1591.

Yeats C, Lees J, Reid A, Kellam P, Martin N, Liu X, Orengo C. 2007. Gene3D: comprehensive structural and functional annotation of genomes. *Nucleic Acids Research* **36**: D414-D418.

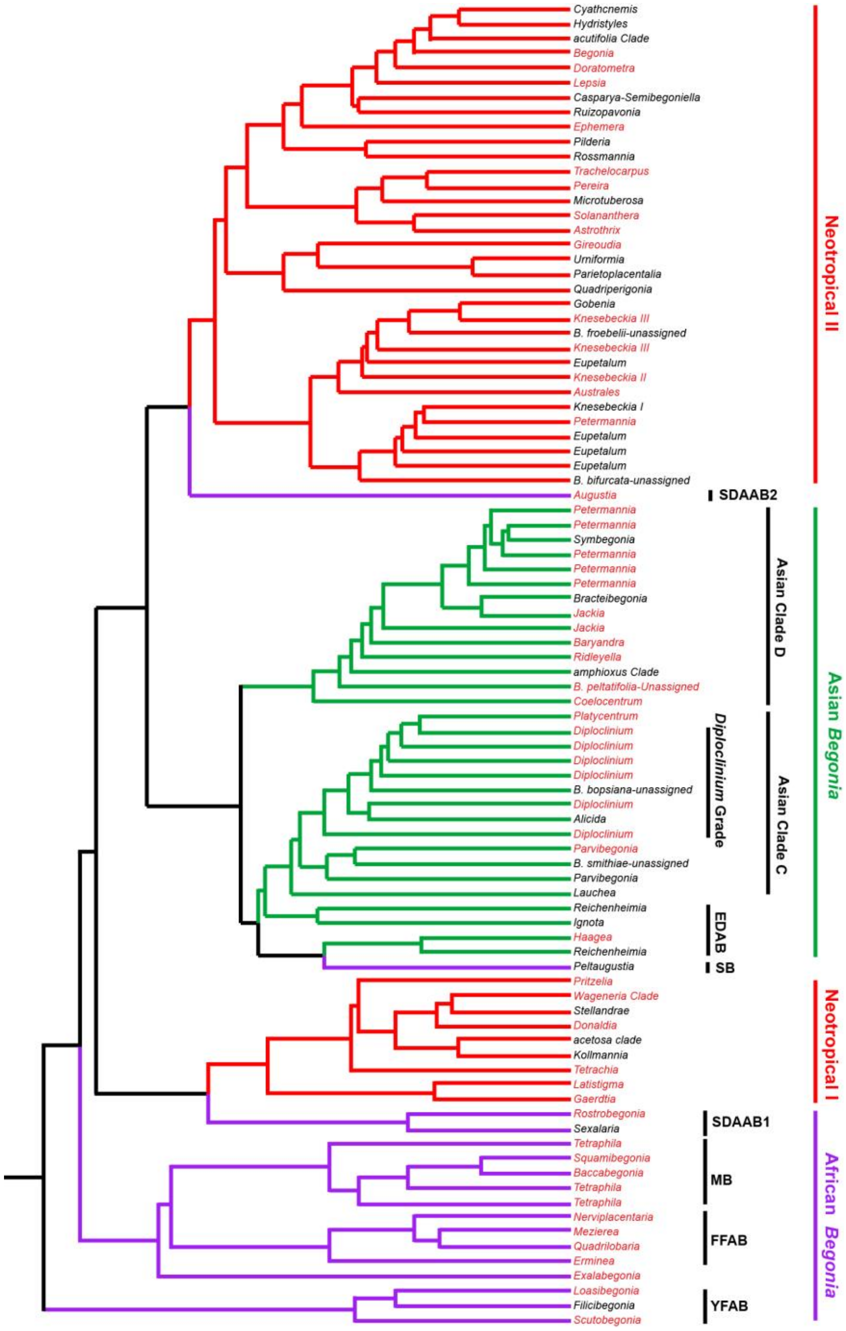

Fig. S1 Current WGS samplings of *Begonia* accessions (78 individuals in 37 sections, as marked in red) on the sectional level *Begonia* phylogeny by Moonlight *et al* (Moonlight *et al.*, 2018). Branches leading to African, Neotropical and Asian accessions were colored in purple, red, and green respectively. Abbreviations: YFAB, Yellow-flowered African *Begonia*; FFAB, Fleshy-fruited African *Begonia*; MB, Malagasy *Begonia*; SDAAB, Seasonally dry adapted African *Begonia*, SB, Socotran *Begonia*; EDAB, Early Diverging Asian *Begonia*.

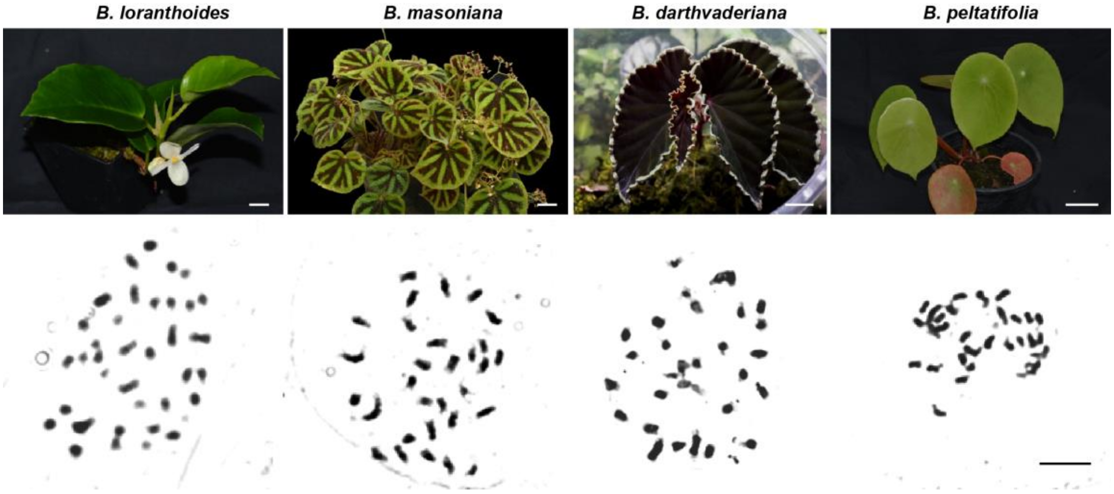

Fig. S2 Somatic chromosome counts at metaphase in the four sequenced *Begonia* species. From right to left: *B. loranthoides* (2n = 38); *B. masoniana* (2n = 30), *B. darthvaderiana* (2n = 30); *B. peltatifolia* (2n = 30). Up: bar = 3 cm, lower: bar = 0.5 µm.

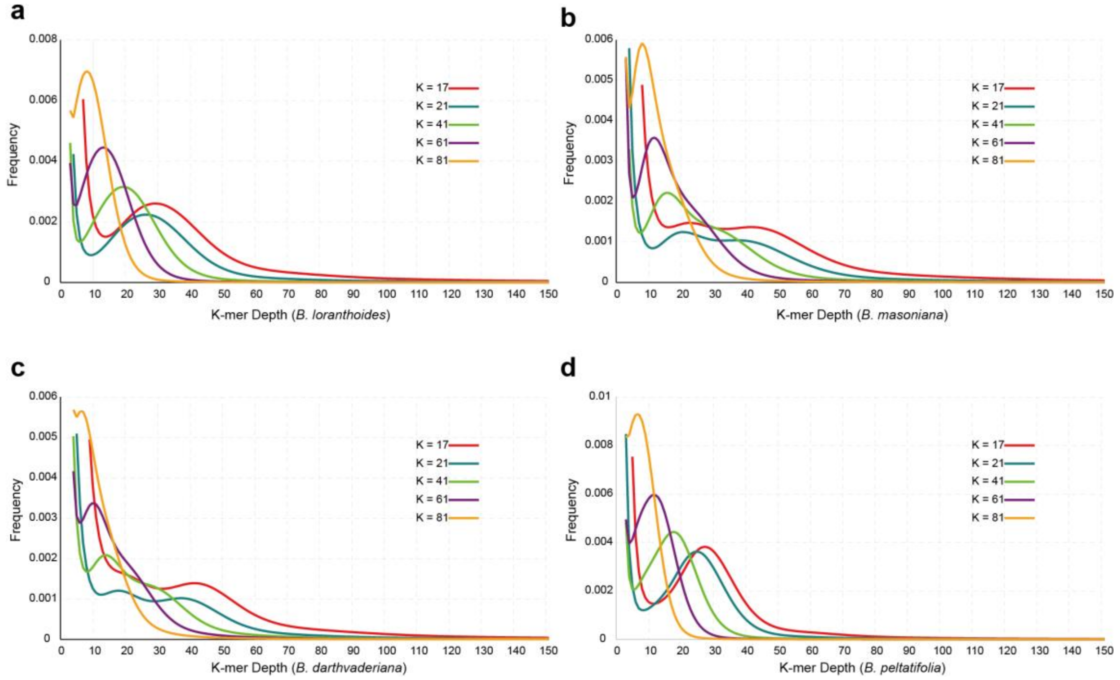

Fig. S3 K-mer analyses of the four *Begonia* species. (a) *B. loranthoides* (genome size~724 Mb); **(b)** *B. masoniana* (genome size ~806 Mb); **(c)** *B. darthvaderiana* (genome size~797 Mb); **(d)** *B. peltatifolia* (genome size ~349 Mb).

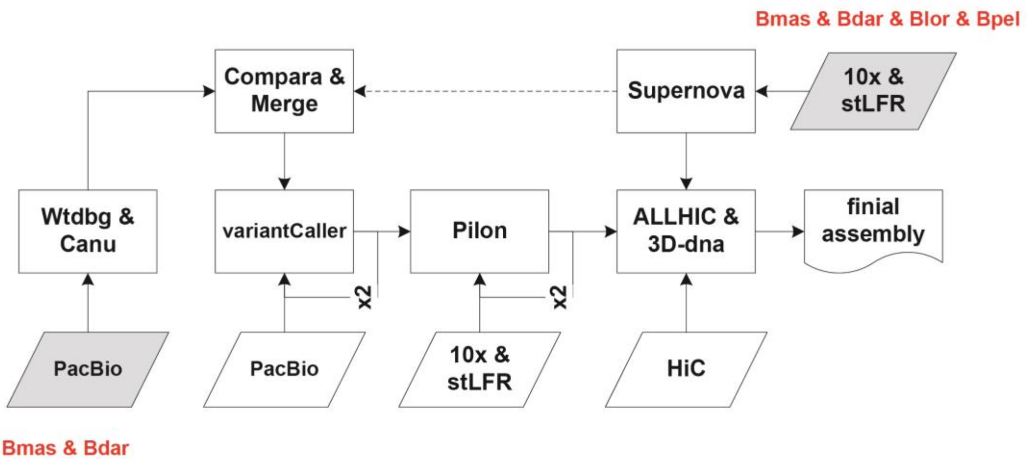

Fig. S4 Flowchart of sequencing and assembly for the four *Begonia* species. Bmas: *B. masoniana*, Bdar: *B. darthvaderiana*, Blor: *B. loranthoids*, Bpel: *B. peltatifolia*.

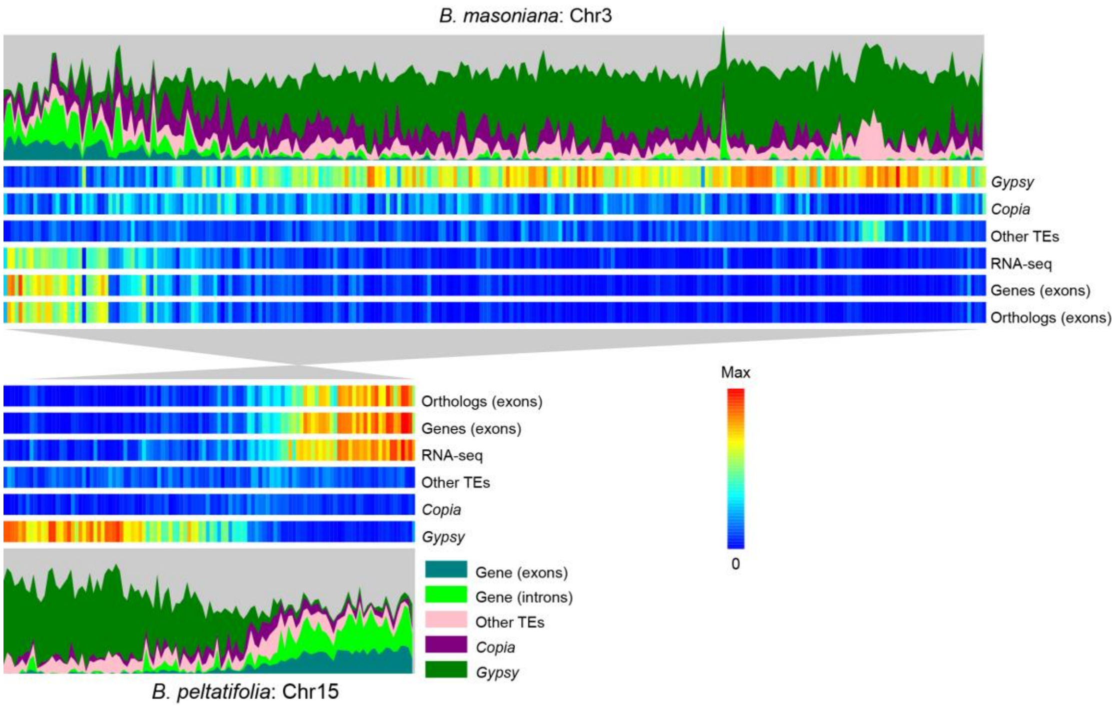

Fig. S5 Scaffold collinear comparisons between two different species (upper: *B. masoniana*, bottom: *B. peltatifolia*) show distinct distribution of different transposon elements.

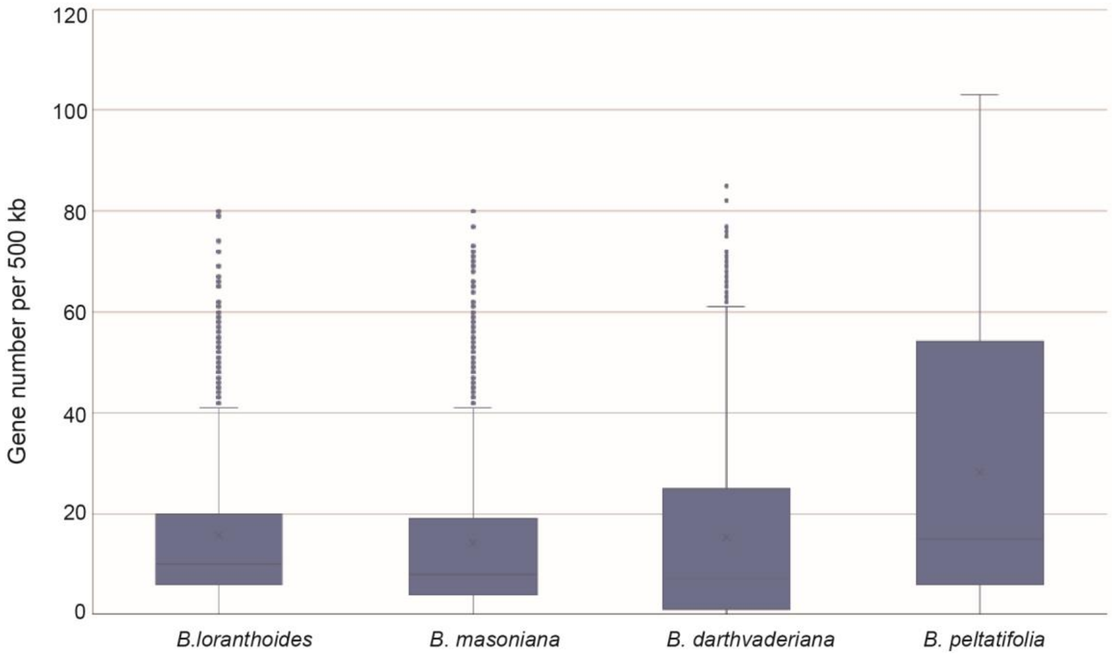

Fig. S6 Distribution of gene density of four *Begonia* genomes. In each box plot, the central rectangle spans the first quartile to the third quartile, the line inside the rectangle shows the median, and the whiskers denote 1.5 interquartile ranges from the box and outlying values plotted beyond the whiskers.

a

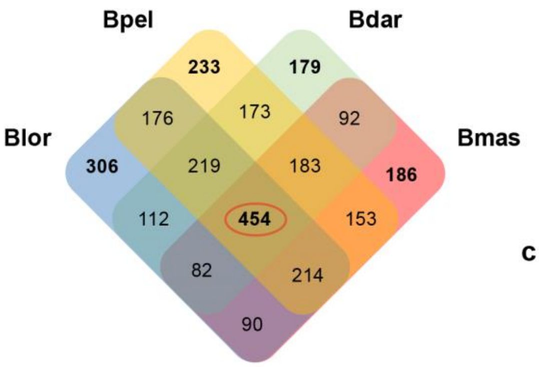

b **GO term enrichment**

GO_ID	GO_Term	Adjusted P value
GO:0034637	cellular carbohydrate biosynthetic process	8.31E-24
GO:0016051	carbohydrate biosynthetic process	2.41E-21
GO:0016020	membrane	3.79E-17
GO:0043168	anion binding	4.28E-15
GO:0044262	cellular carbohydrate metabolic process	4.93E-14
GO:0036094	small molecule binding	9.51E-14
GO:0000166	nucleotide binding	1.51E-13
GO:1901265	nucleoside phosphate binding	1.51E-13
GO:0050660	flavin adenine dinucleotide binding	2.14E-13
GO:0015291	secondary active transmembrane transporter activity	7.05E-13

c **KEGG pathway enrichment**

MapID	MapTitle	Adjusted P value
map00562	Inositol phosphate metabolism	1.02E-13
map04070	Phosphatidylinositol signaling system	1.26E-07
map00030	Pentose phosphate pathway	6.75E-06
map00500	Starch and sucrose metabolism	9.69E-06
map00480	Glutathione metabolism	0.000821103
map00062	Fatty acid elongation	0.000821103
map00052	Galactose metabolism	0.000821103
map00908	Zeatin biosynthesis	0.004357455
map04075	Plant hormone signal transduction	0.004417006
map00450	Selenocompound metabolism	0.012029562

Fig. S7 Analyses of post-WGD retained genes families in *Begonia*. (a) Venn diagram showing the number of gene families shared among four *Begonia* species. Blor, *B. loranthoides*; Bmas, *B. masoniana*; Bdar, *B. darthvaderiana*; Bpel, *B. peltatifolia*. **(b)** GO term enrichment of the shared 454 gene families. Only top ten terms were shown. **(c)** KEGG pathway enrichment of the shared 454 gene families.

a

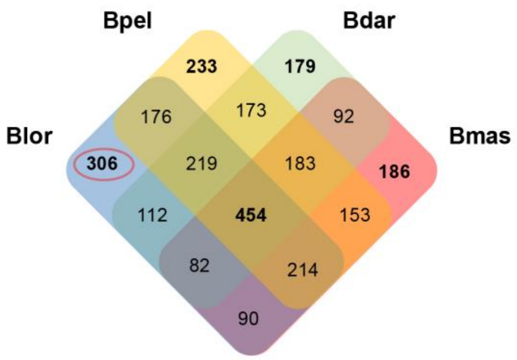

b **GO term enrichment**

GO_ID	GO_Term	Adjusted P value
GO:0008270	zinc ion binding	5.32E-25
GO:0046914	transition metal ion binding	4.08E-19
GO:0046872	metal ion binding	6.31E-10
GO:0043169	cation binding	8.83E-09
GO:0043167	ion binding	3.35E-06
GO:0097159	organic cyclic compound binding	0.000510095
GO:1901363	heterocyclic compound binding	0.000510095
GO:0005488	binding	0.009373642
GO:0004144	diacylglycerol O-acyltransferase activity	0.013381934
GO:0016411	acylglycerol O-acyltransferase activity	0.013381934

c **KEGG pathway enrichment**

MapID	MapTitle	Adjusted P value
map00073	Cutin, suberine and wax biosynthesis	0.001986804
map00600	Sphingolipid metabolism	0.022666042
map00020	Citrate cycle (TCA cycle)	0.040693729

Fig. S8 Analysis of post-WGD retained genes families specific to *B. loranthoides*. **(a)** Venn diagram showing the number of gene families shared among four *Begonia* species. Blor, *B. loranthoides*; Bmas, *B. masoniana*; Bdar, *B. darthvaderiana*; Bpel, *B. peltatifolia*. **(b)** GO term enrichment of the 306 gene families specifically retained in *B. loranthoides*. Only top ten terms were shown. **(c)** KEGG pathway enrichment of the specific 306 gene families.

a

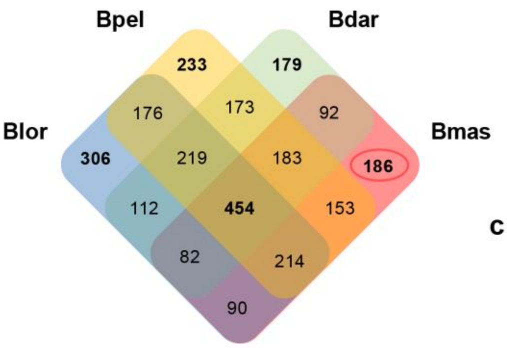

b GO term enrichment

GO_ID	GO_Term	Adjusted P value
GO:0016755	transferase activity, transferring amino-acyl groups	0.00051178
GO:0016603	glutaminyl-peptide cyclotransferase activity	0.00051178
GO:0017186	peptidyl-pyroglutamic acid biosynthetic process, using glutaminyl-peptide cyclotransferase	0.00051178
GO:0046961	proton-transporting ATPase activity, rotational mechanism	0.00051178
GO:0018199	peptidyl-glutamine modification	0.00051178
GO:0036442	hydrogen-exporting ATPase activity	0.00051178
GO:0006334	nucleosome assembly	0.000639478
GO:0031497	chromatin assembly	0.000639478
GO:0034728	nucleosome organization	0.000639478
GO:0006323	DNA packaging	0.000639478

c KEGG pathway enrichment

MapID	MapTitle	Adjusted P value
lemap00940	Phenylpropanoid biosynthesis	4.65E-16
map03010	Ribosome	1.24E-08
map03008	Ribosome biogenesis in eukaryotes	4.74E-08
map04712	Circadian rhythm - plant	4.74E-08
map00460	Cyanoamino acid metabolism	4.93E-07
map00360	Phenylalanine metabolism	1.20E-06
map00130	Ubiquinone and other terpenoid-quinone biosynthesis	7.85E-05
map02010	ABC transporters	0.000506192
map04120	Ubiquitin mediated proteolysis	0.004737909
map00945	Stilbenoid, diarylheptanoid and gingerol biosynthesis	0.016339929
map04145	Phagosome	0.017306632
map00402	Benzoxazinoid biosynthesis	0.024135485
map04130	SNARE interactions in vesicular transport	0.024135485
map00941	Flavonoid biosynthesis	0.025663683
map00760	Nicotinate and nicotinamide metabolism	0.02676336

Fig. S9 Analysis of post-WGD retained genes families specific to *B. masoniana*. (a) Venn diagram showing the number of gene families shared among four *Begonia* species. Blor, *B. loranthoides*; Bmas, *B. masoniana*; Bdar, *B. darthvaderiana*; Bpel, *B. peltatifolia*. **(b)** GO term enrichment of the 186 gene families specifically retained in *B. masoniana*. Only top ten terms were shown. **(c)** KEGG pathway enrichment of the specific 186 gene families.

a

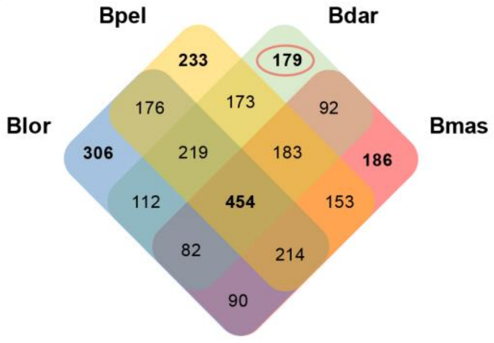

b GO term enrichment

GO_ID	GO_Term	Adjusted P value
GO:0006839	mitochondrial transport	0.002820676
GO:0008097	5S rRNA binding	0.002820676
GO:0009405	pathogenesis	0.011026311
GO:0004719	protein-L-isoaspartate (D-aspartate) O-methyltransferase activity	0.011026311
GO:0005742	mitochondrial outer membrane translocase complex	0.011026311
GO:0098799	outer mitochondrial membrane protein complex	0.011026311
GO:0008199	ferric iron binding	0.011026311
GO:0019773	proteasome core complex, alpha-subunit complex	0.011026311
GO:0006850	mitochondrial pyruvate transport	0.011026311
GO:0004418	hydroxymethylbilane synthase activity	0.011026311

c KEGG pathway enrichment

MapID	MapTitle	Adjusted P value
map00330	Arginine and proline metabolism	0.000838368
map00944	Flavone and flavonol biosynthesis	0.001209885
map00965	Betalain biosynthesis	0.003709008
map00943	Isoflavonoid biosynthesis	0.020127517
map03040	Spliceosome	0.020127517
map00196	Photosynthesis - antenna proteins	0.028235292
map00785	Lipoic acid metabolism	0.028235292

Fig. S10 Analysis of post-WGD retained genes families specific to *B. darthvaderiana*. (a) Venn diagram showing the number of gene families shared among four *Begonia* species. Blor, *B. loranthoides*; Bmas, *B. masoniana*; Bdar, *B. darthvaderiana*; Bpel, *B. peltatifolia*. **(b)** GO term enrichment of the 179 gene families specifically retained in *B. darthvaderiana*. Only top ten term were shown. **(c)** KEGG pathway enrichment of the specific 179 gene families.

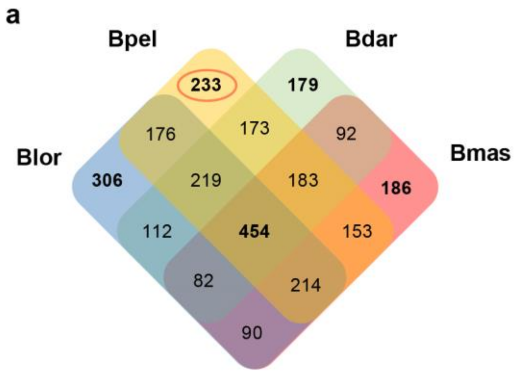

b GO term enrichment

GO_ID	GO_Term	Adjusted P value
GO:0065007	biological regulation	0.000148545
GO:0050794	regulation of cellular process	0.000148545
GO:0050789	regulation of biological process	0.000227383
GO:0004553	hydrolase activity, hydrolyzing O-glycosyl compounds	0.002228777
GO:0016798	hydrolase activity, acting on glycosyl bonds	0.004342495
GO:0006355	regulation of transcription, DNA-templated	0.004342495
GO:0051252	regulation of RNA metabolic process	0.004342495
GO:1903506	regulation of nucleic acid-templated transcription	0.004342495
GO:2001141	regulation of RNA biosynthetic process	0.004342495
GO:0019219	regulation of nucleobase-containing compound metabolic process	0.004342495

c KEGG pathway enrichment

MapID	MapTitle	Adjusted P value
map04712	Circadian rhythm - plant	0.00896238

Fig. S11 Analysis of post-WGD retained gene families specific to *B. peltatifolia*. (a) Venn diagram showing the number of gene families shared among four *Begonia* species. Blor, *B. loranthoides*; Bmas, *B. masoniana*; Bdar, *B. darthvaderiana*; Bpel, *B. peltatifolia*. **(b)** GO term enrichment of the 233 gene families specifically retained in *B. peltatifolia*. Only top ten terms were shown. **(c)** KEGG pathway enrichment of the specific 233 gene families.

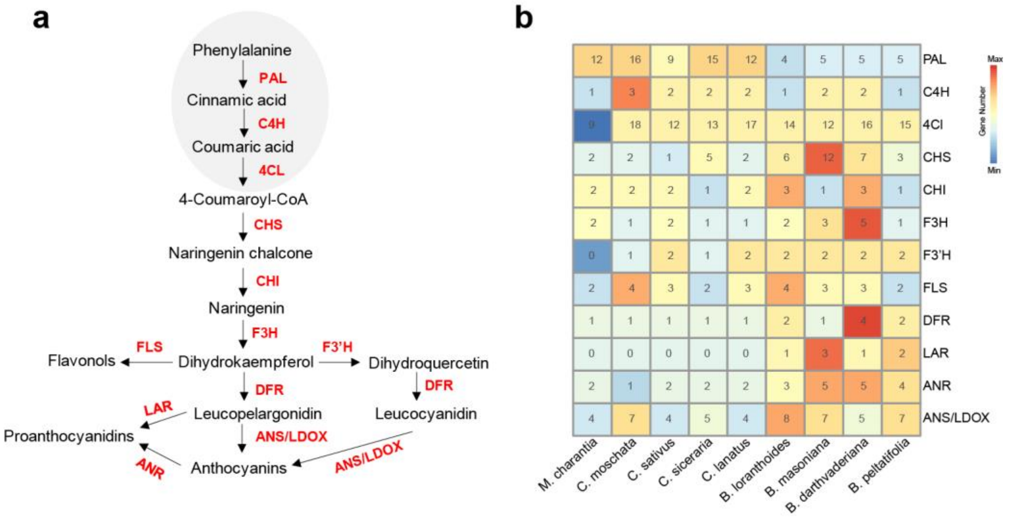

Fig. S12 Expansion of gene families in anthocyanin pathway in *Begonia*. (a) Overview of the anthocyanin biosynthetic pathway with the general phenylpropanoid pathway indicated in grey shade. **(b)** Gene families in the anthocyanin biosynthesis that are expanded in *Begonia*. Enzyme abbreviations: PAL, phenylalanine ammonia lyase; C4H, cinnamate 4-hydroxylase4; 4CL, 4-coumaroyl:CoA ligase; CHS, chalcone synthase; CHI, chalcone isomerase; F3H, flavanone 3-hydroxylase; F3'H, flavonoid 3' hydroxylase; DFR, dihydroflavonol 4-reductase; LAR, anthocyanidin reductase; ANR, anthocyanidin reductase; ANS/LDOX, anthocyanidin synthase/leucoanthocyanidin dioxygenase.

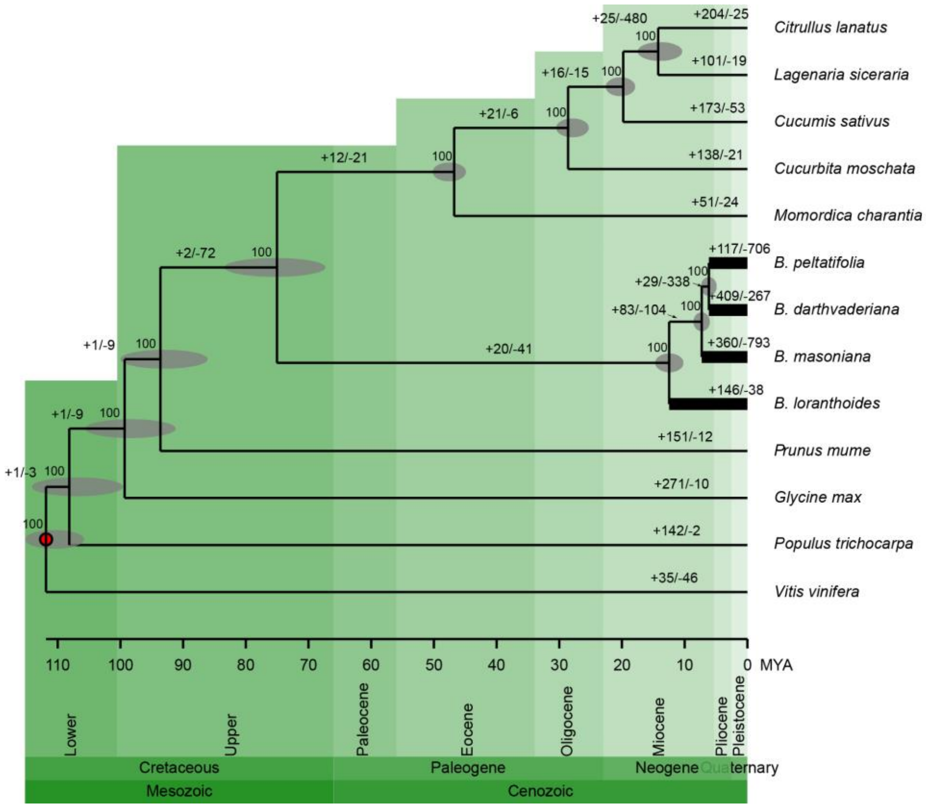

Fig. S13 Gene family expansions and contractions along a dated angiosperm phylogeny of 13 selected species. The numbers of significantly (*p*-value < 0.01) expanded and contracted gene families are shown above the branches. Fossil calibration point and divergence times are indicated by red dot and grey ovals at the internodes, respectively. The range of the ovals indicates the 95% confidence interval of the divergence time.

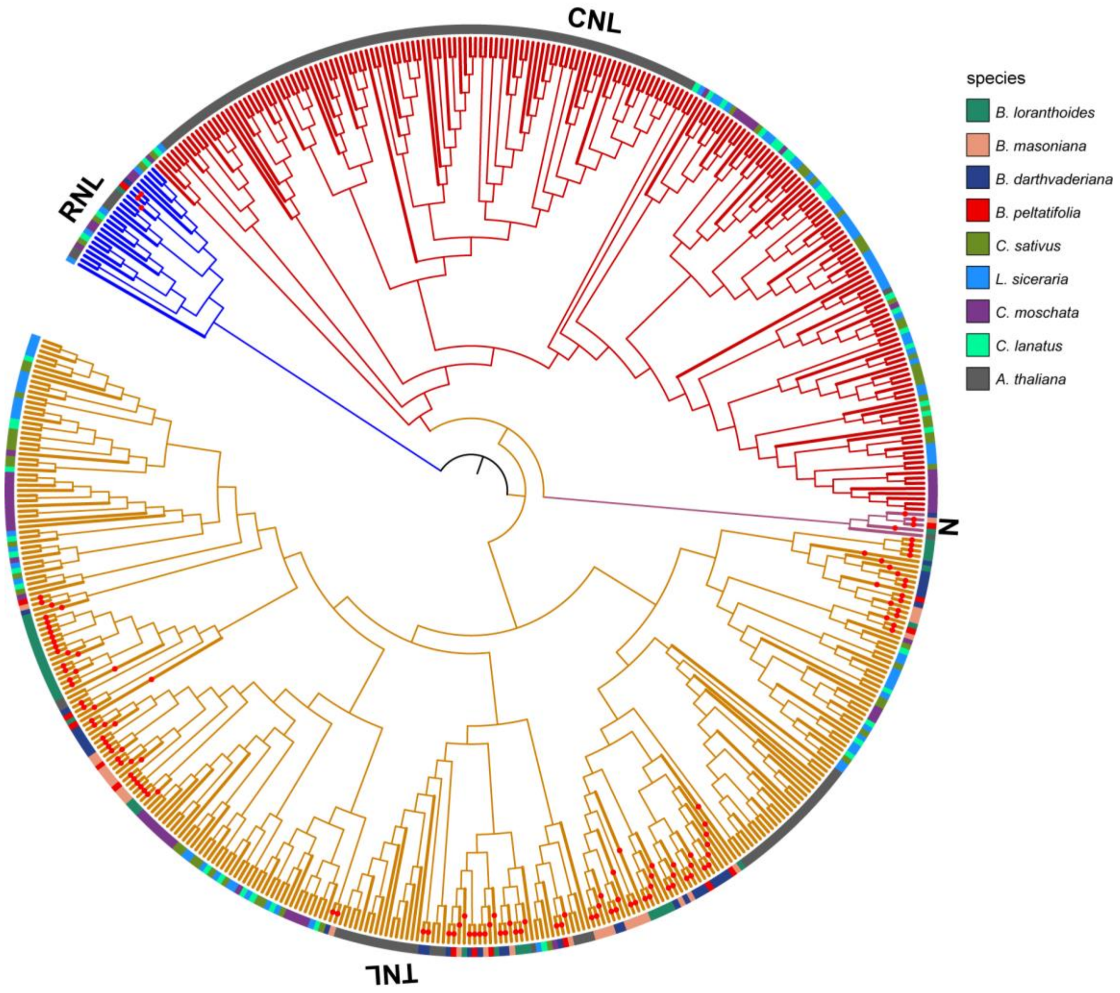

Fig. S14 Contraction and complete loss of the TNL subgroup of NBS family in *Begonia*. Homologues of NBS genes of the four *Begonia* species are highlighted by red circle upon the branch.

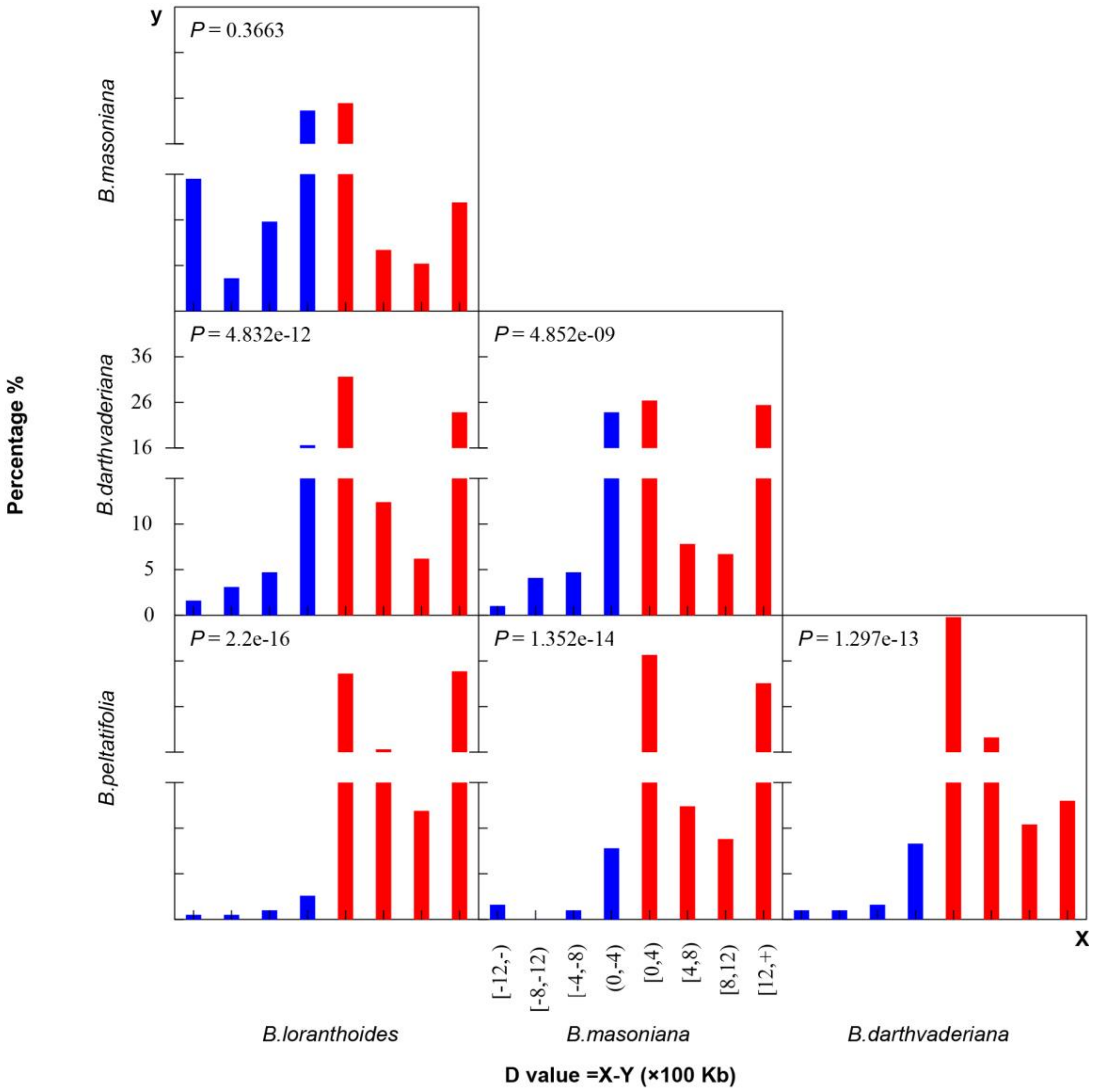

Fig. S15 Comparison of TE proportions in 122 shared syntenic blocks across four *Begonia* species. D value indicates difference value range of TE size from right species minus that of left. Red and blue bars indicate positive and negative values, respectively. *P* values indicates levels of difference significance between positive and negative values.

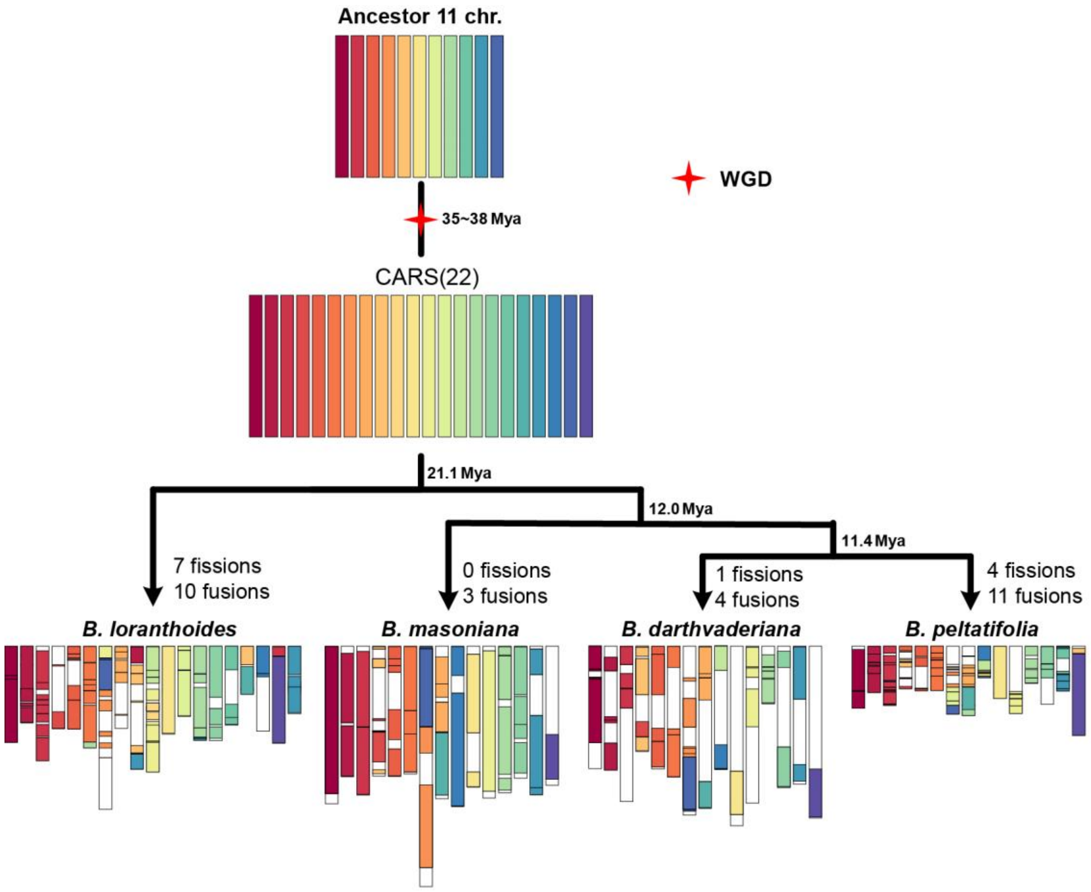

Fig. S16 Reconstruction of paleo-genome of four sequenced *Begonia* species. CARS, conserved ancestral regions.

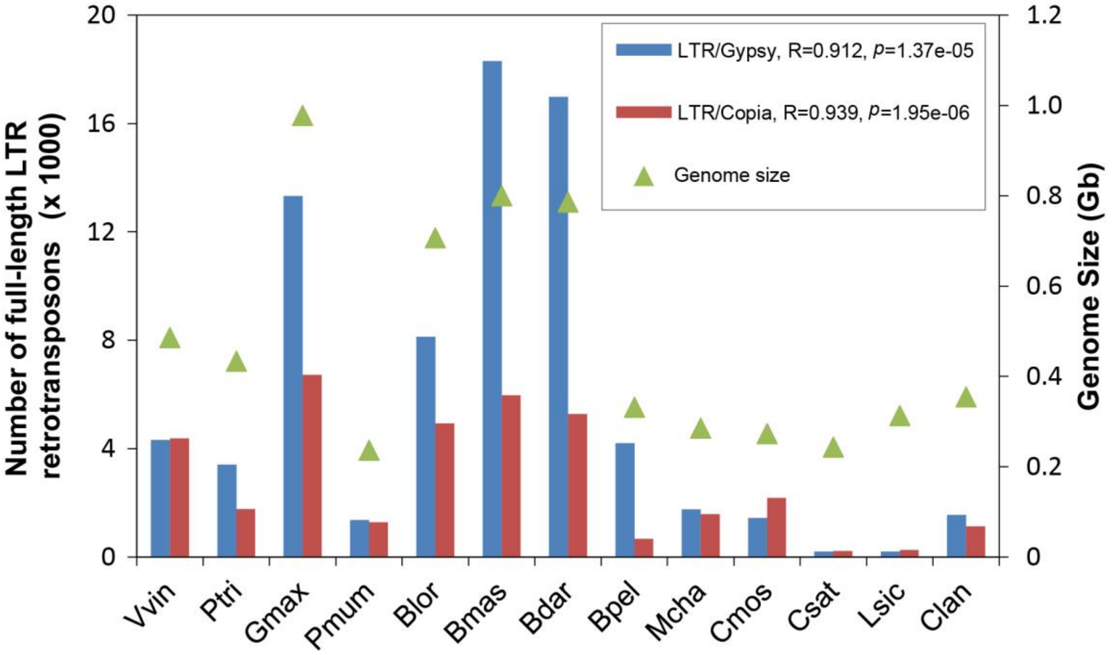

Fig. S17 Number of LTR insertions and genome sizes for 13 angiosperm species. Vvin, *Vitis vinifera*; Ptri, *Populus trichocarpa*; Gmax, *Glycine max*; Pmum, *Prunus mume*; Mcha, *Momordica charantia*; Cmos, *Cucurbita moschata*; Cast, *Cucumis sativus*; Lsic, *Lagenaria siceraria*; Clan, *Citrullus lanatus*.

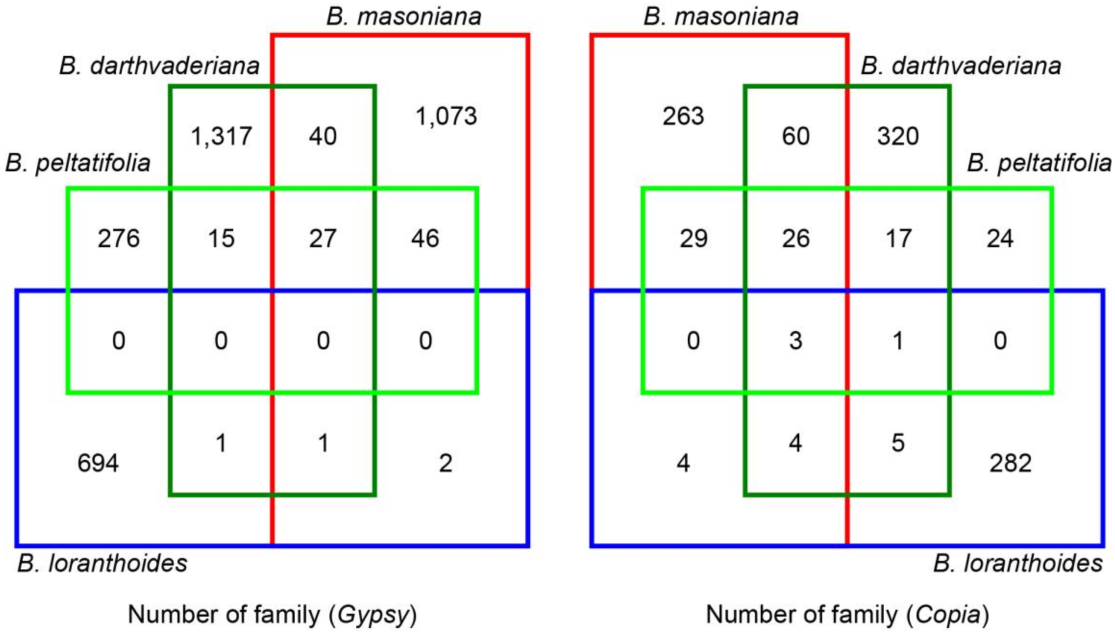

Fig. S18 Number of shared full length LTR families across four *Begonia* species.

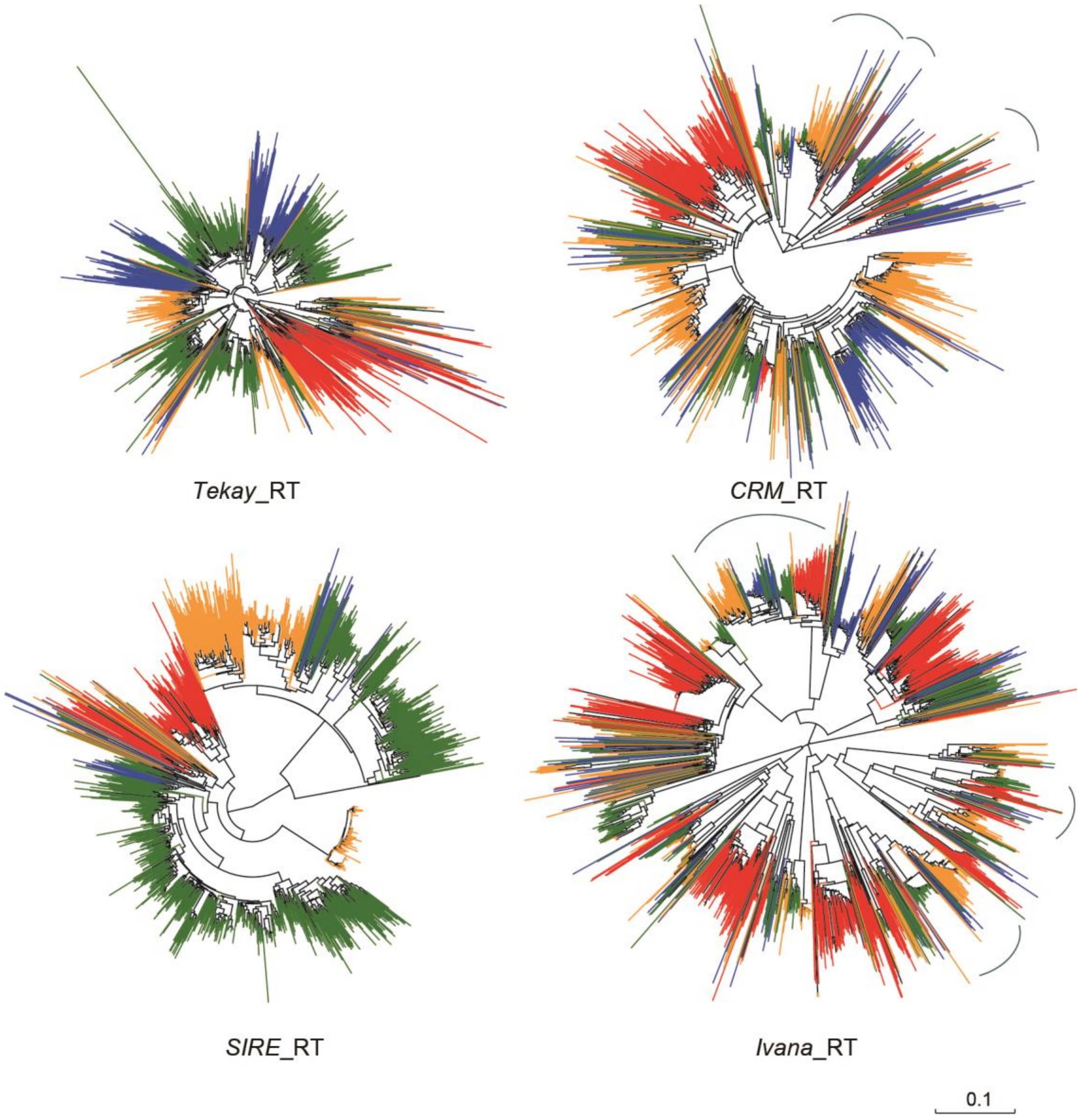

Fig. S19 Neighbor-joining trees built from RT domain sequence similarities among different lineage-specific copies identified in *Begonia* genomes. *B. loranthoides*: red, *B. masoniana*: green, *B. darthvaderiana*: orange, *B. peltatifolia*: blue.

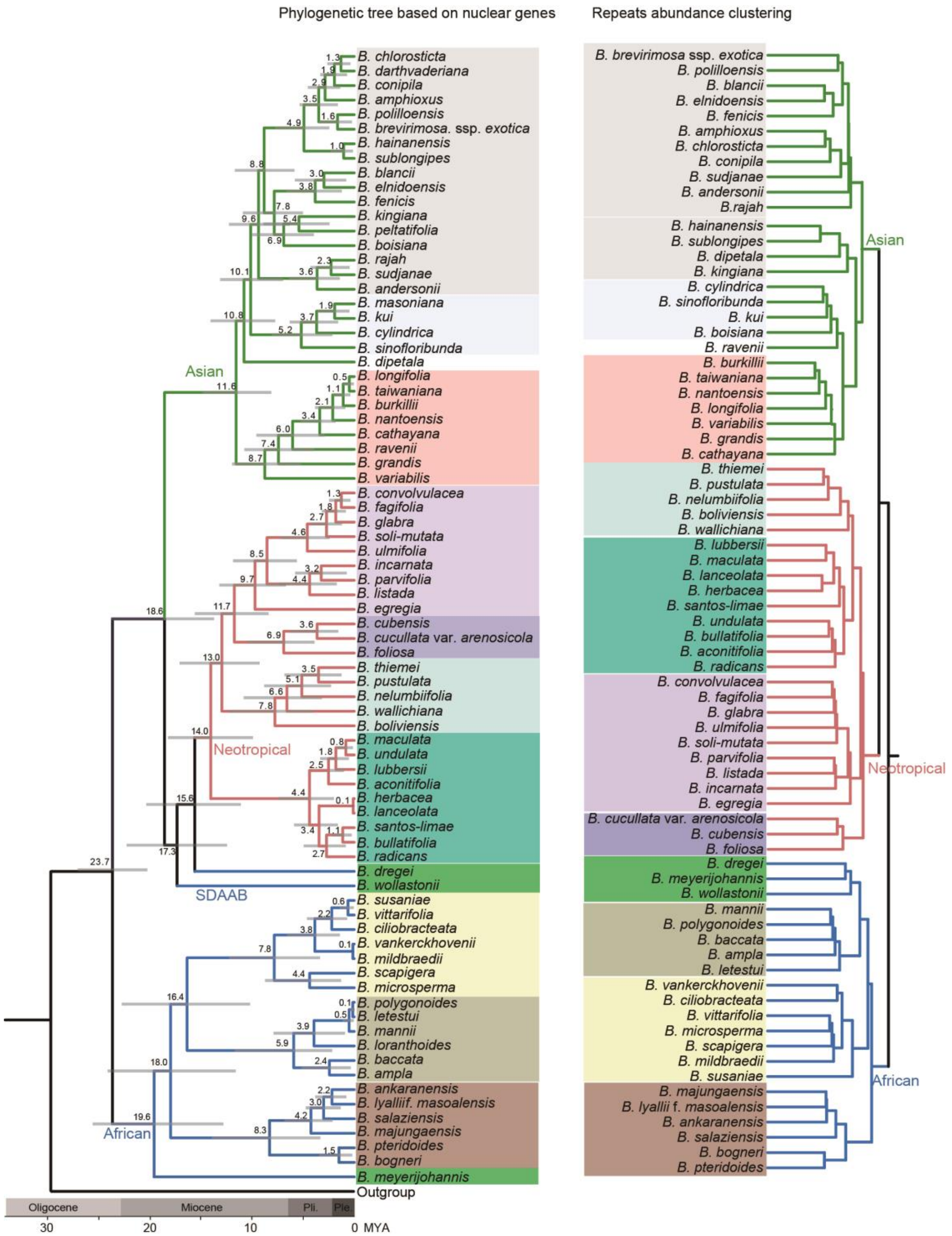

Fig. S20 Comparison of nuclear ML tree and abundance clustering of TEs.
Clades of species found in both trees are colored the same.

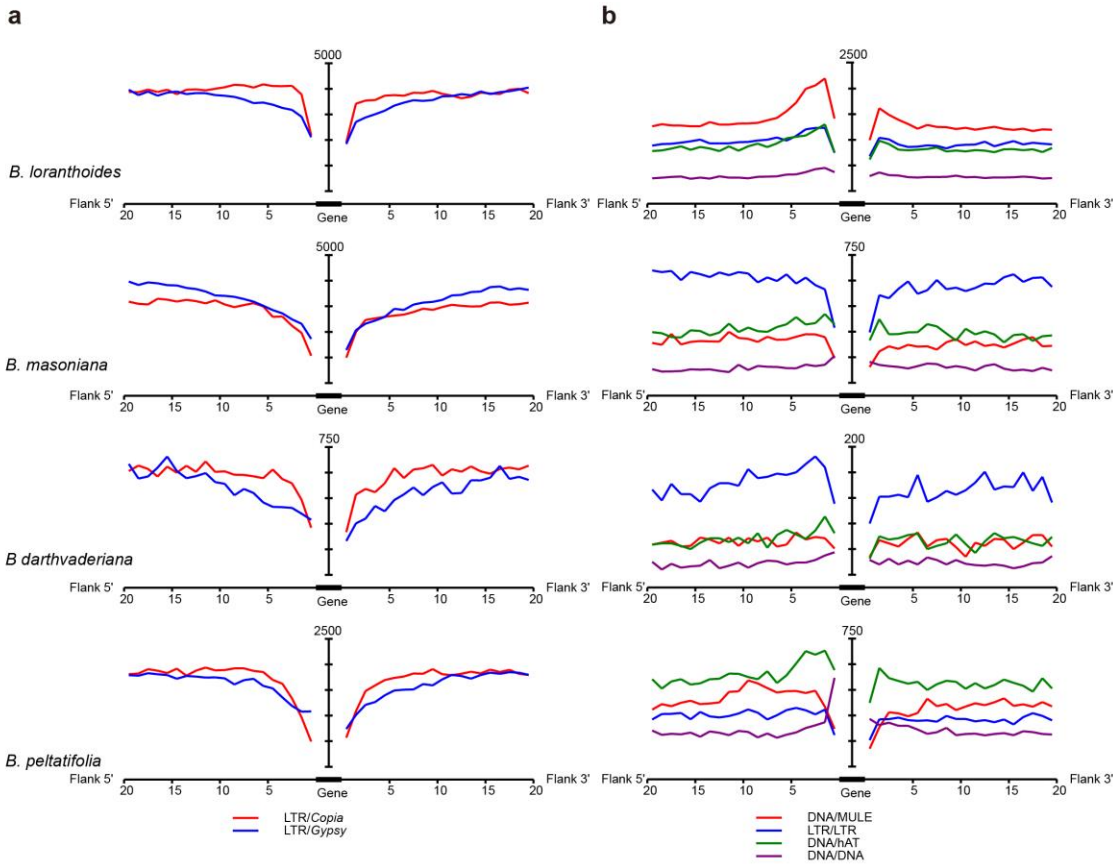

Fig. S21 The TE landscape surrounding genes in four *Begonia* species. For all genes, the 10 kb upstream of the TSS and 10 kb downstream of the TEs were analyzed. Abundance of the different TE families was compiled for all genes of each genome. **(a)** The distributions of superfamily *Copia* and *Gypsy* surrounding genes. **(b)** The distribution of TE family DNA/MULE, LTR/LTR, DNA/hAT and DNA/DNA surrounding genes.

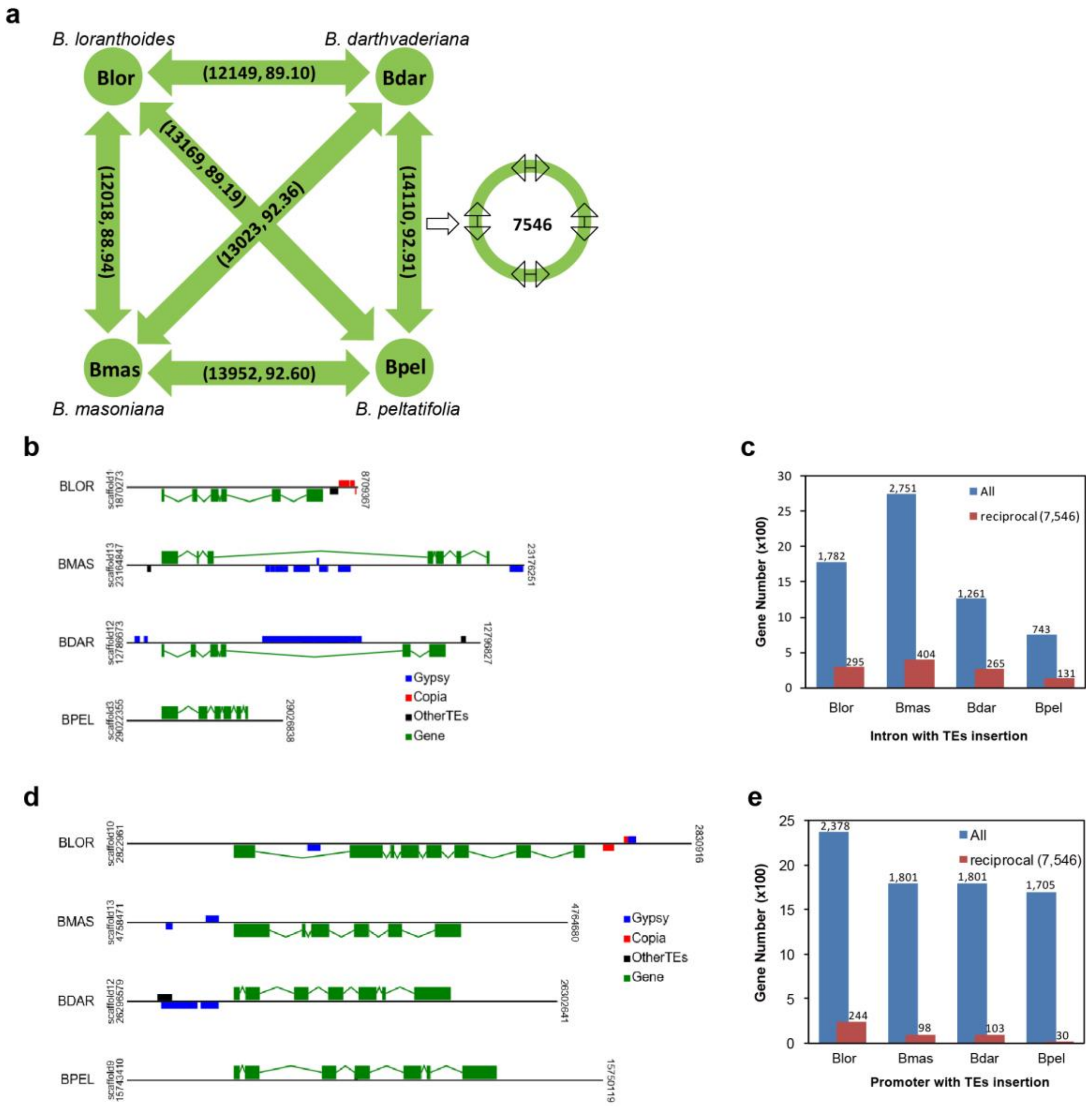

Fig. S22 Impacts of TE insertions on the structure of introns and promoters. (a) Schematic diagram showing the identification of 7,546 likely orthologs across four *Begonia* species by inter-comparisons. Numbers within the bracket: gene number, average homologous similarity. **(b)** A representative example showing differential insertions of TEs in the introns of orthologs. **(c)** Number of genes with TE insertions in introns. **(d)** An representative example showing different TE insertions on promoters of the ortholog among four *Begonia* species. **(e)** Number of genes with TE insertion in promoters.

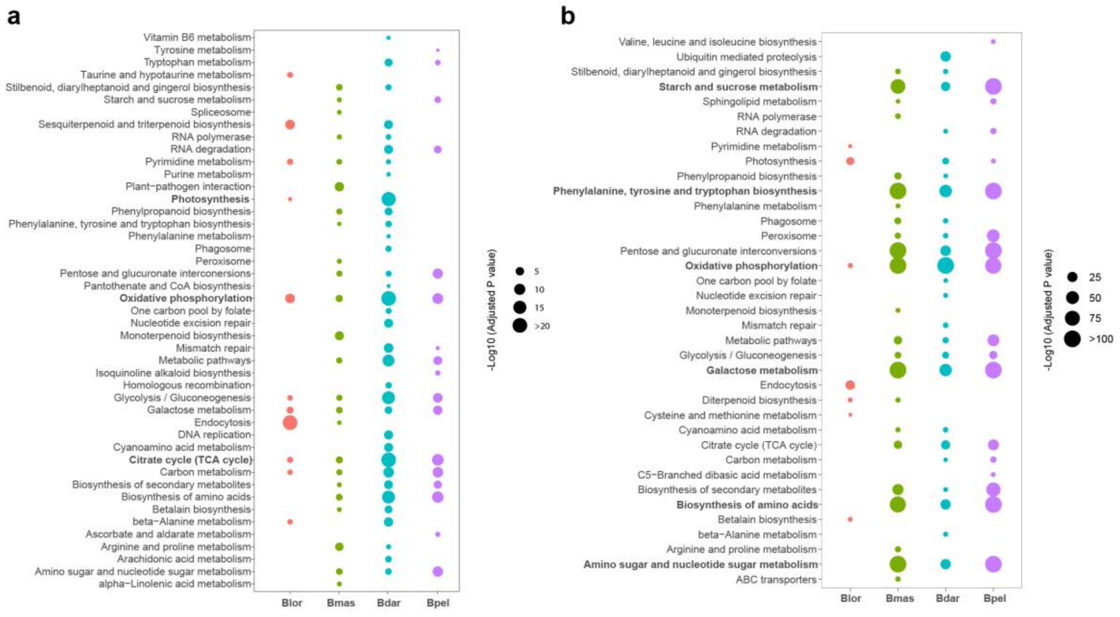

Fig. S23 KEGG enrichment of genes with TE insertion either in introns or promoters. (a) Function enrichment of genes with TE insertion in introns. **(b)** Function enrichment of genes with TE insertion in promoters.

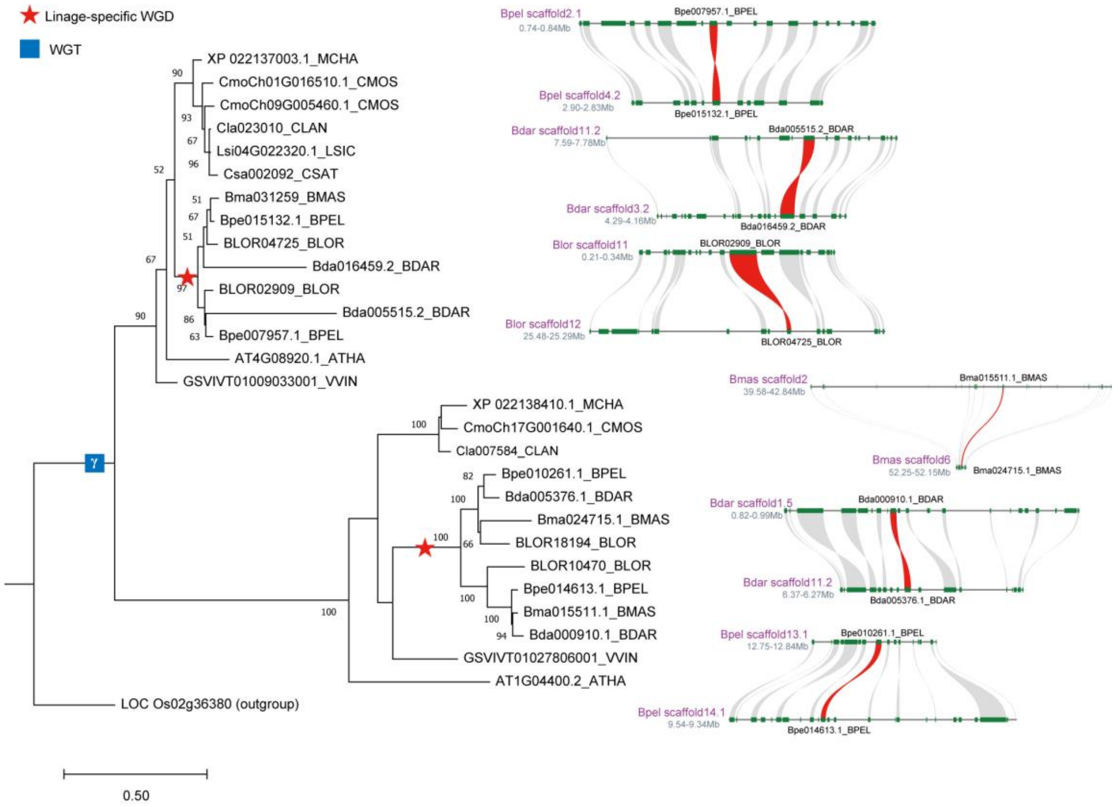

Fig. S24 Expansion of crychromes (CRYs) genes in *Begonia* due to WGD. The stars indicate the Begoniaceae specific WGD event. Syntenic blocks were placed on the right of the tree. Numbers on branches show the bootstrap supporting values. Species name abbreviations were indicated after the gene ID. VVIN, *Vitis vinifera*; MCHA, *Momordica charantia*; CMOS, *Cucurbita moschata*; CAST, *Cucumis sativus*; LSIC, *Lagenaria siceraria*; CLAN, *Citrullus lanatus*; ATHA, *Arabidopsis thaliana*; BLOR, *B. loranthoides*; BMAS, *B. masoniana*; BDAR, *B. darthvaderiana*; BPEL, *B. peltatifolia*. Same below.

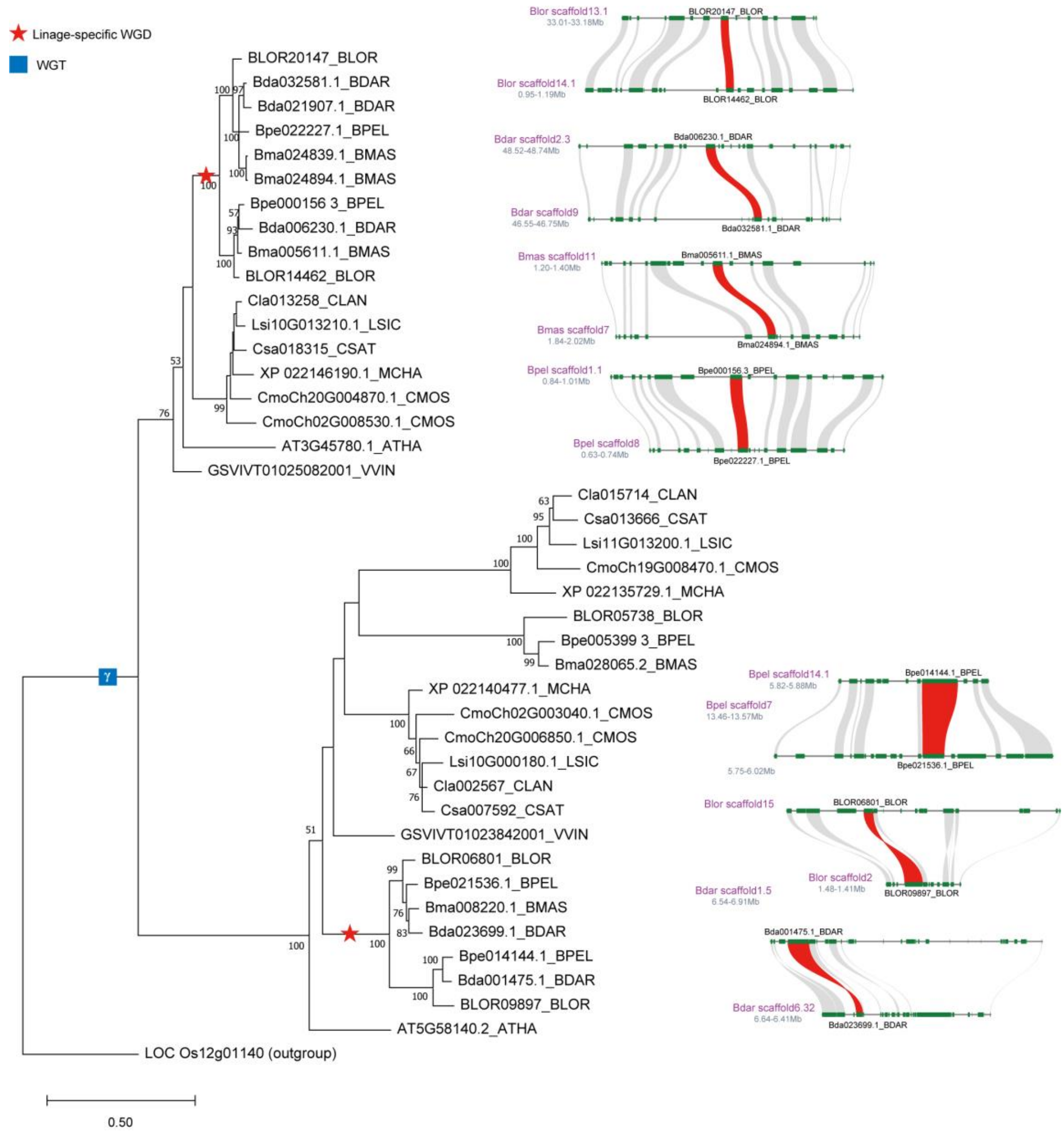

Fig. S25 Expansion of Phototropin (PHOT) genes in *Begonia* due to WGD. The stars indicate the Begoniaceae specific WGD event. Syntenic blocks were placed on the right of the tree. Numbers on branches show the bootstrap supporting values.

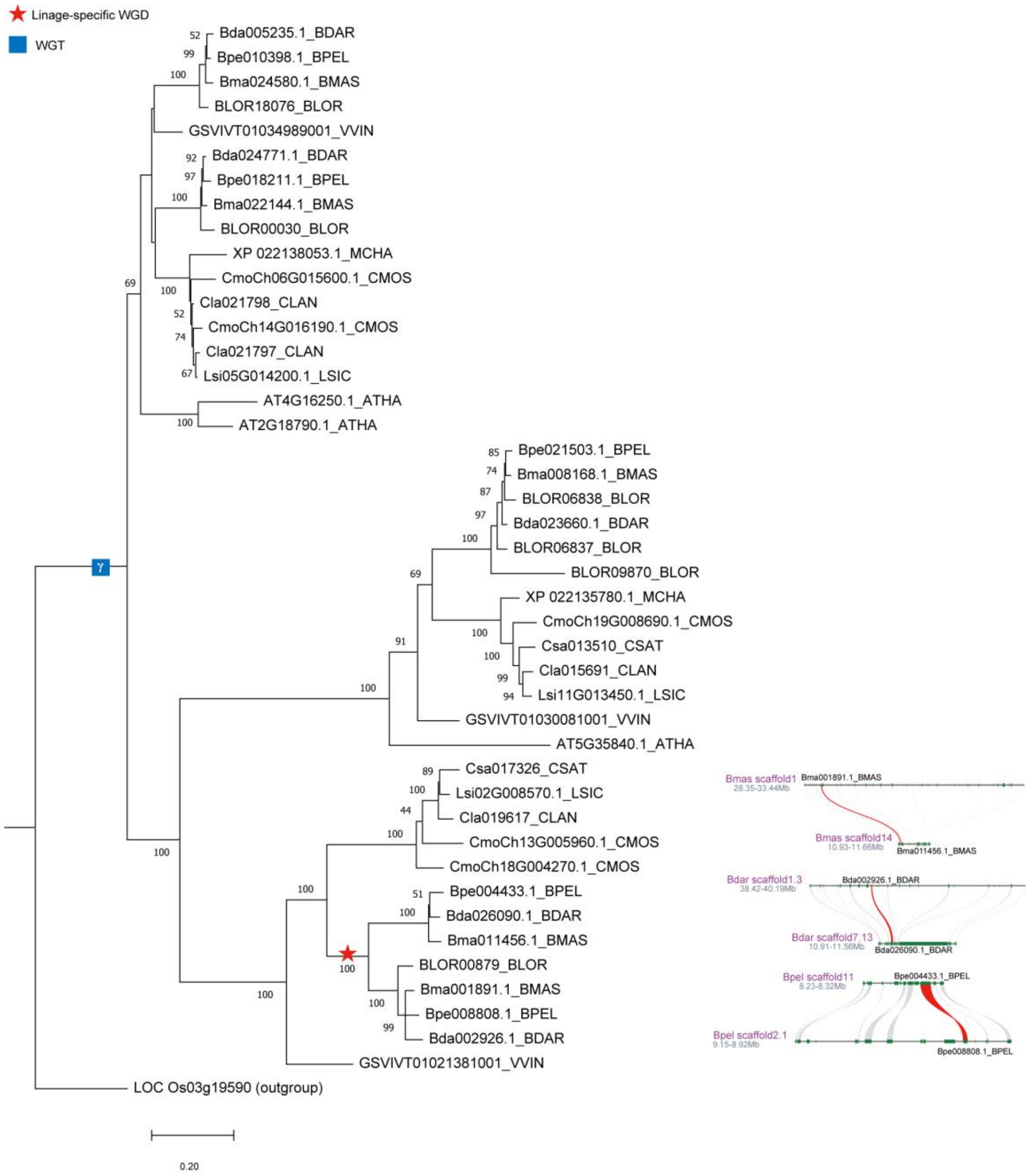

Fig. S26 Expansion of Phytochrome (PHY) genes in *Begonia* due to WGD. The stars indicate the Begoniaceae specific WGD event. Syntenic blocks were placed on the right of the tree. Numbers on branches show the bootstrap supporting values.

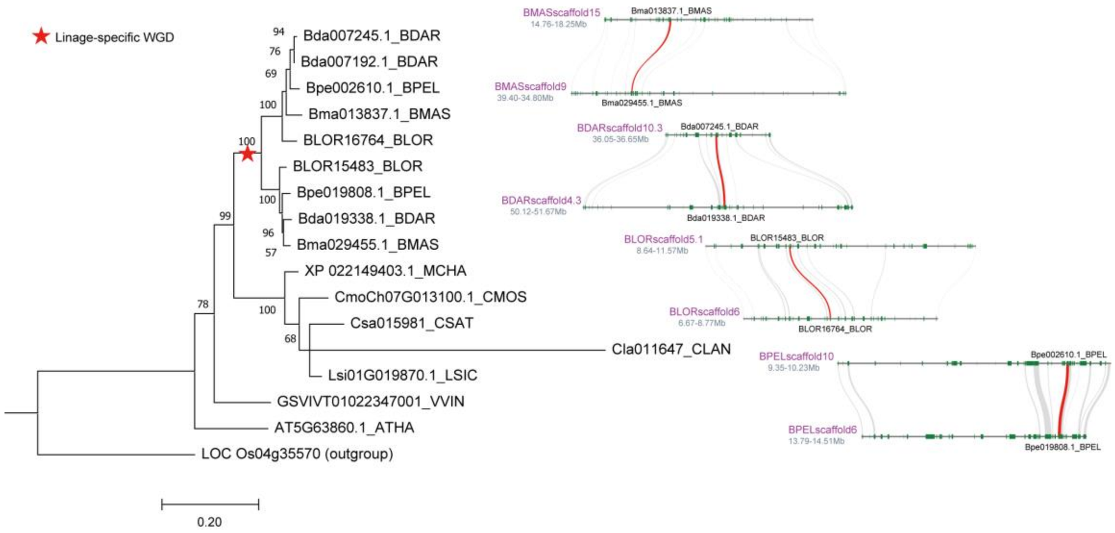

Fig. S27 Expansion of UV Resistance Locus 8 (UVR8) genes in *Begonia* due to WGD. The star indicates the Begoniaceae specific WGD event. Syntenic blocks were placed on the right of the tree. Numbers on branches show the bootstrap supporting values.

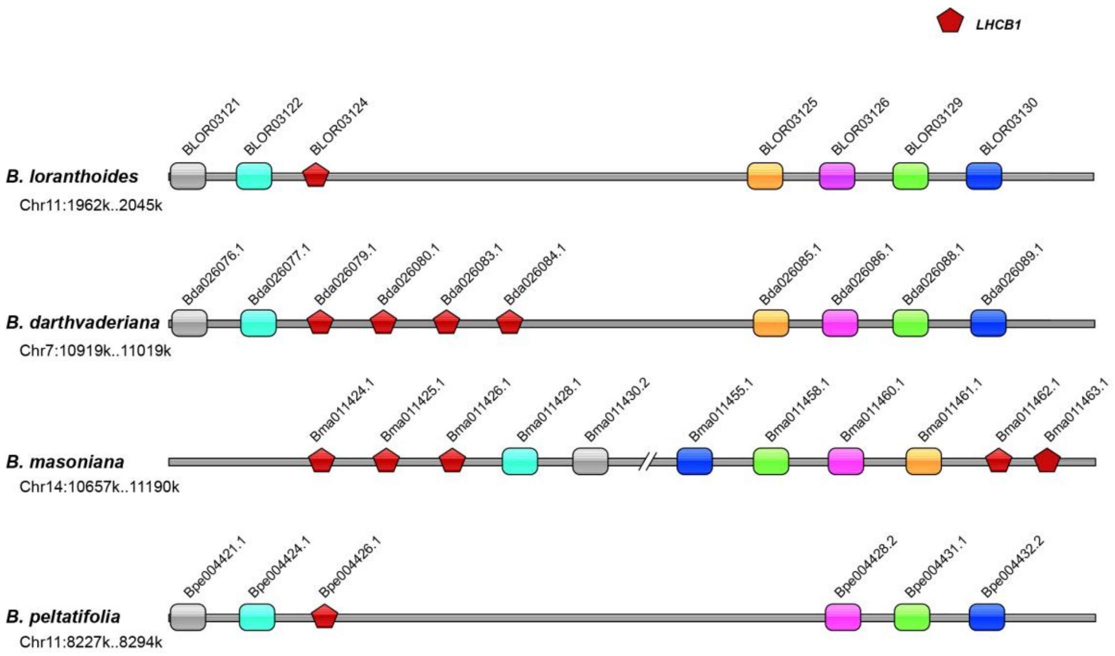

Fig. S28 Schematic diagrams show tandem duplication of LHCB1 genes in *B. masoniana* and *B. darthvaderiana*. The syntenic genes are indicated as same boxes.

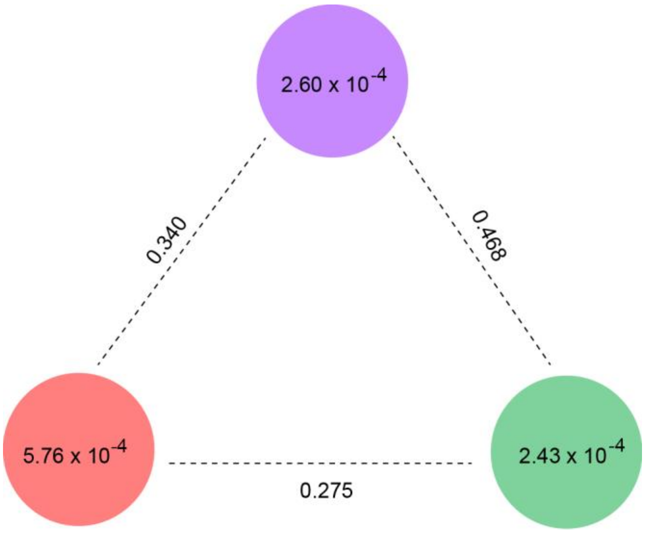

Fig. S29 Nucleotide diversity (*π*) and population divergence (*F*ST) across the three major groups of *Begonia*. Purple, African group; red, Neotropical group; green, Asian group. The value in each circle represents a measure of nucleotide diversity for this group, and the value on each line indicates the population divergence between the two groups.

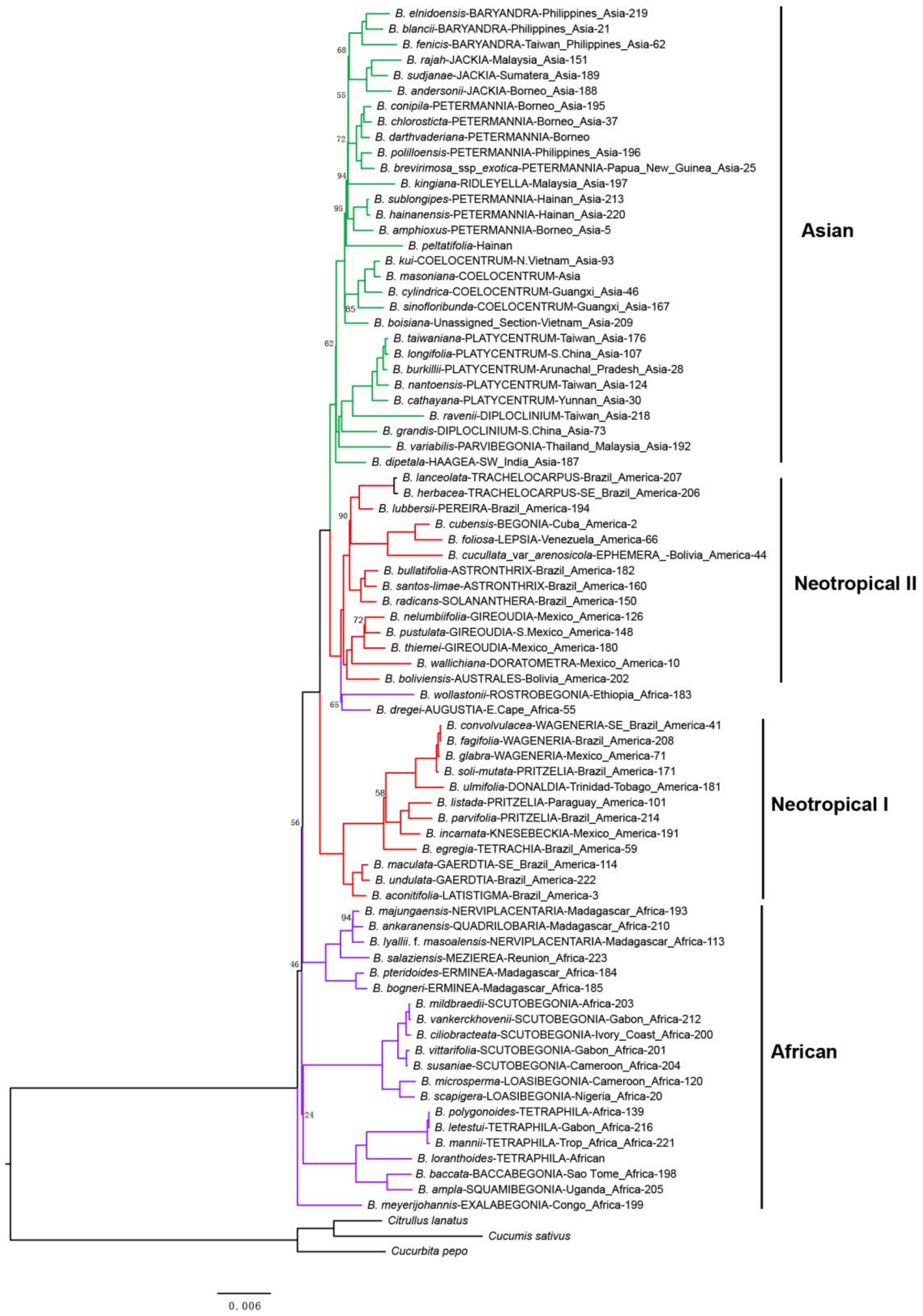

Fig. S30 Maximum likelihood tree inferred from concatenated nucleotide sequences of *Begonia* plastid protein coding genes using RAxML. Branches are maximally supported unless otherwise indicated. Branches leading to African, Neotropical, and Asia accessions are indicated in purple, red, and green, respectively. Three species (*Citrullus lanatus*, *Cucumis stativus*, *Cucurbita pepo*) of Cucurbitaceae were used as outgroups.

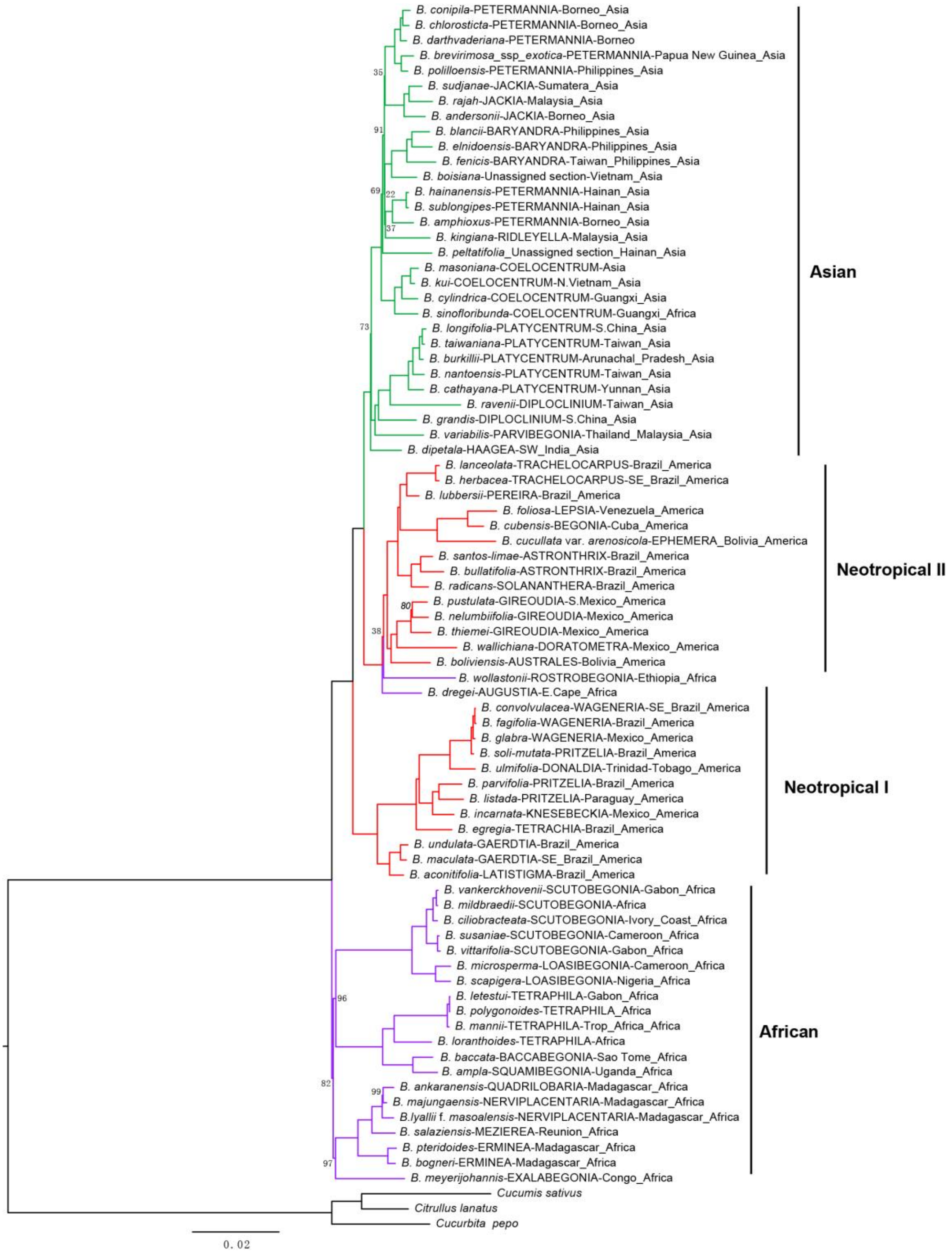

Fig. S31 Maximum likelihood tree inferred from *Begonia* plastome nucleotide alignment of 156,131 bp using RAxML. Branches are maximally supported unless otherwise indicated. Branches leading to African, Neotropical, and Asia accessions are indicated in purple, red, and green, respectively.
Three species (*Citrullus lanatus, Cucumis stativus, Cucurbita pepo*) of Cucurbitaceae were used as outgroups.

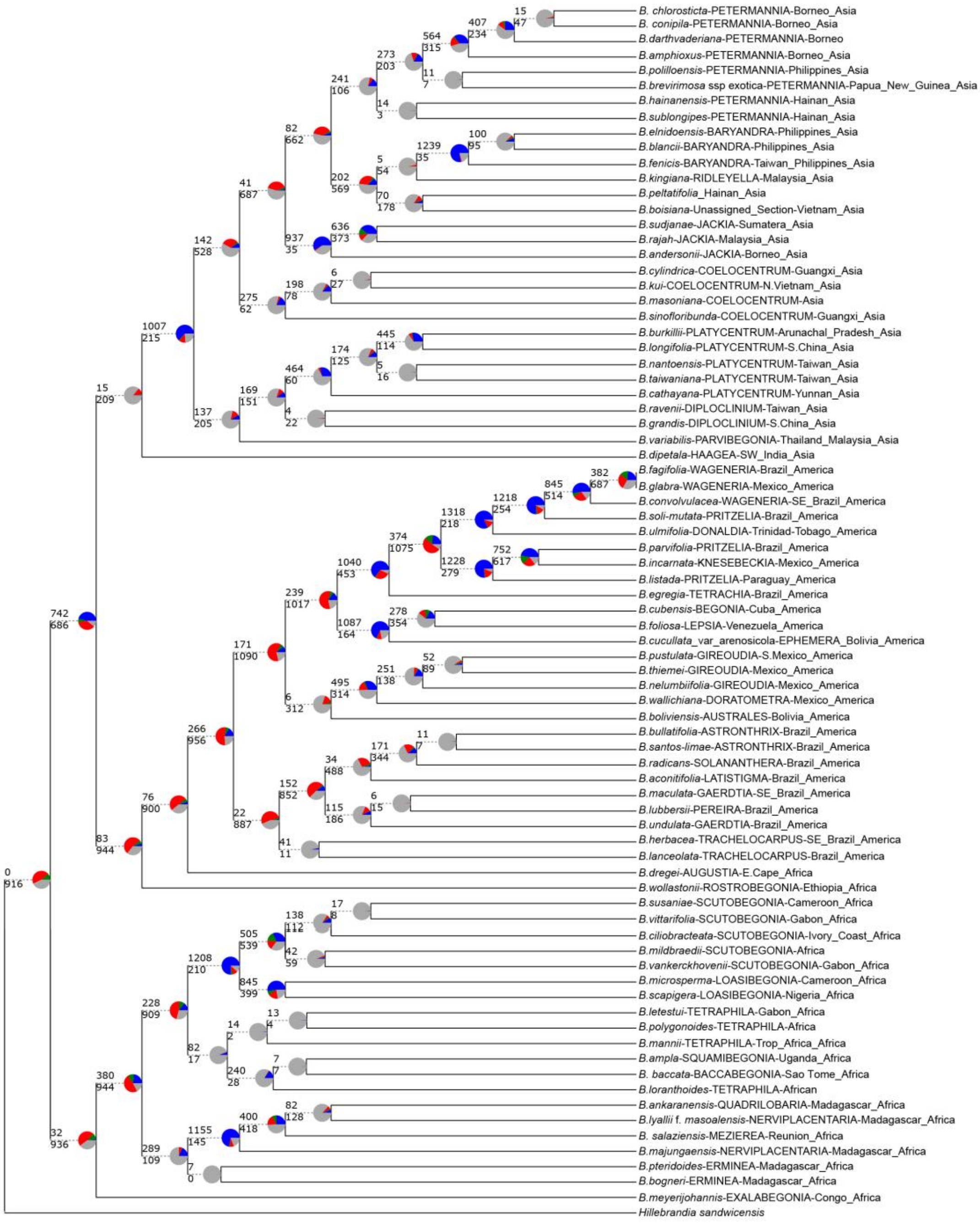

Fig. S32 Maximum likelihood tree inferred from a concatenated dataset of 1,604 nuclear genes using IQtree with individual gene trees mapped. Four colors of the pie chart: concordance (blue), top conflict that supporting a single main alternative topology (green), other conflicts that supporting various alternative topologies (red), no signal (gray). *Hillebrandia sandwicensis* was used as outgroup.

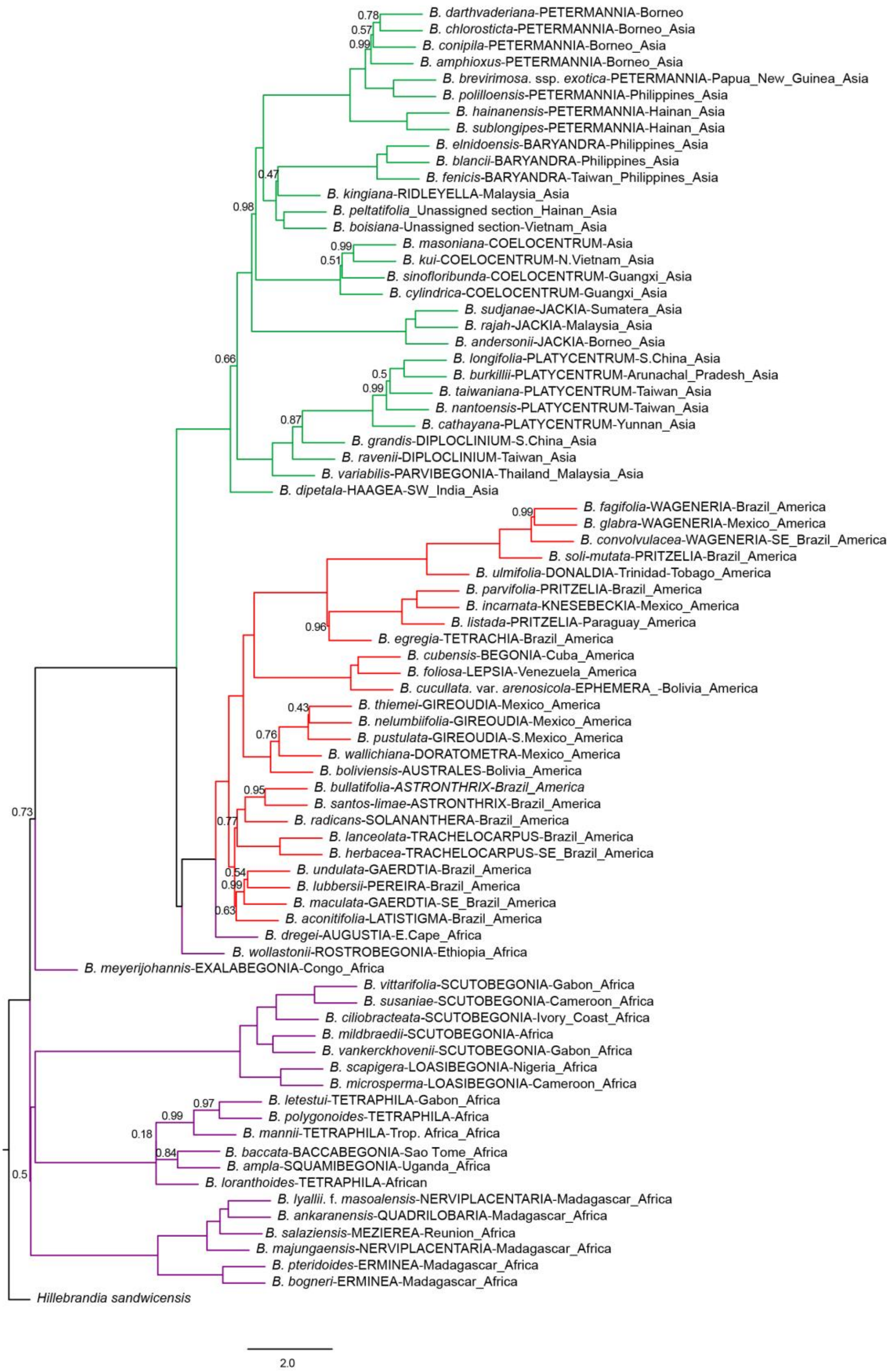

Fig. S33 Coalescent super tree inferred with ASTRAL-III using 1,604 nuclear single gene trees. Branches are maximally supported unless otherwise indicated. Branches leading to African, Neotropical, and Asian accessions are indicated in purple, red, and green, respectively. *Hillebrandia sandwicensis* was used as outgroup.

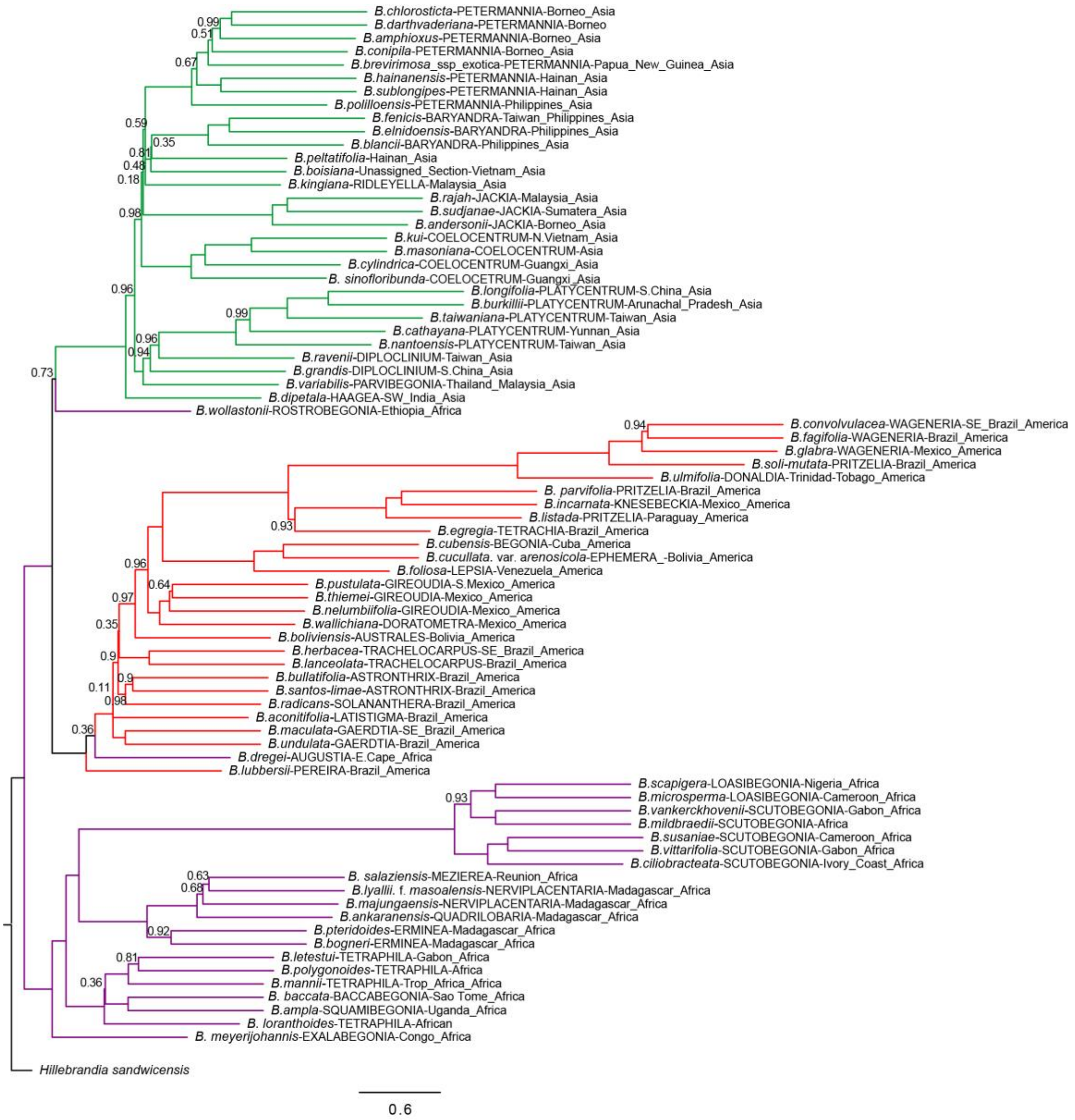

Fig. S34 Coalescent super tree inferred with ASTRAL-III using SNPs in 1,343 nuclear single gene trees. Branches are maximally supported unless otherwise indicated. Branches leading to African, Neotropical, and Asian accessions are indicated in purple, red, and green, respectively. *Hillebrandia sandwicensis* was used as outgroup.

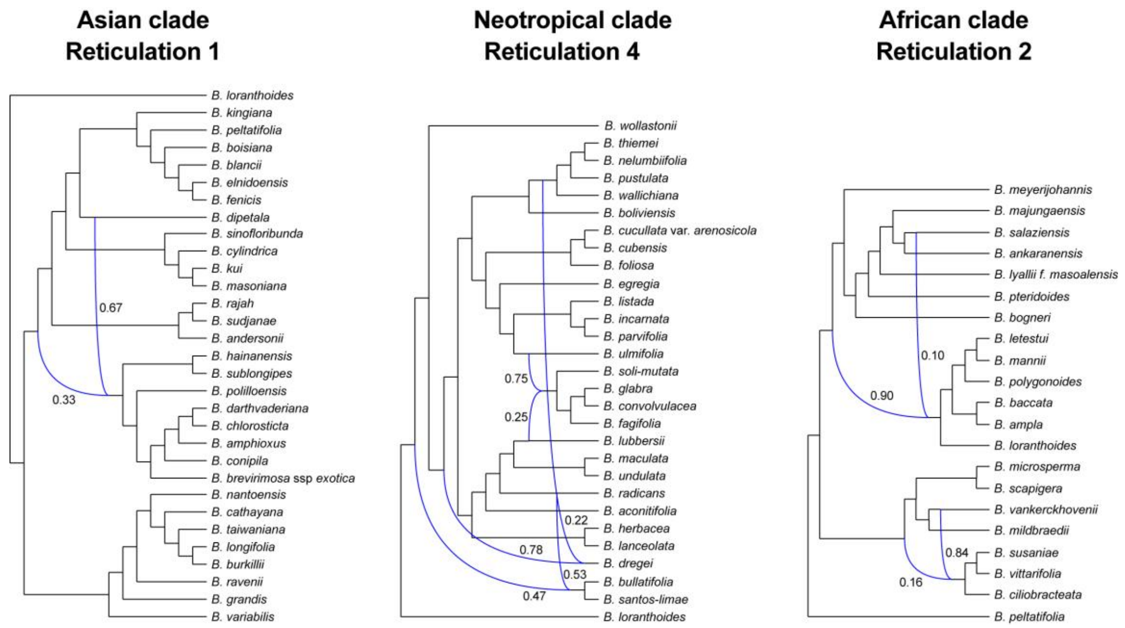

Fig. S35 Phylonet network results for three geographically delimited *Begonia* clades. Only the reticulation with best Log probability is shown. For compromisation of the computational burden, we split the *Begonia* phylogeny into three geographically delimited *Begonia* clades. For each of the three analyses, we first inferred the species trees with IQtree with the SNP datasets for the single copy gene region, with a minimum sequence length of 100 bp and minimum taxa occupancy of 75%. The individual gene trees finally used are 548, 548, 661 for Asian, Neotropical, and African datasets, respectively. The option of InferNetwork_MPL option as implemented in Phylonet is used to infer the species network for each *Begonia* clade with reticulations from 1 to 10. Blue curved lines indicate lineages involved in reticulated histories, and numerical values are the inheritance probabilities for each reticulation.

Table S1 Summary of 78 *Begonia* species for whole genome shot gun sequencing.
(See separate Excel file)

Table S2 Genome size estimation based on K-mer analysis.

**Species**	**K**	**Kmer Number (D>5)**	**Peak Depth**	**Genome Size**
B. loranthoides	21	18,827,386,084	26	724,130,234
B. masoniana	21	29,818,513,231	37	805,905,763
B. darthvaderiana	21	29,490,349,982	37	797,036,486
B. peltatifolia	21	8,733,478,350	25	349,339,134

Table S3 Summary of within-genome heterozygosity of the four *Begonia* species.

Species	SNP/Indel	Total	Heterozygosity rate
B. loranthoides	SNP	1,216,560	0.17%
	Indel	124,674	0.02%
B. masoniana	SNP	7,159,899	0.90%
	Indel	490,247	0.06%
B. darthvaderiana	SNP	7,229,727	0.92%
	Indel	124,674	0.06%
B. peltatifolia	SNP	791,618	0.24%
	Indel	97,889	0.03%

Table S4 Statistics of genome assemblies.

Data, Method	Statistical level: scaffold; contig (bp)	*B. loranthoides*	*B. masoniana*	*B. darthvaderiana*	*B. peltatifolia*
		>500; >500	>500; >500	>500; >500	>500; >500
10 × genomics, Supernova	Total number (>)	24,414; 35,341	110,753; 138,701	143,516; 175,652	10,658; 15,403
	Total length of (bp)	716,442,159; 688,788,849	970,512,287; 872,750,467	911,958,493; 858,665,223	334,086,415; 327,069,085
	Gap number (bp)	27,653,310; 0	97,761,820; 0	53,293,270; 0	7,017,330; 0
	N50 Length (bp)	6,733,575; 85,570	94,737; 14,943	28,284; 12,997	3,195,172; 99,958
	N90 Length (bp)	23,247; 10,987	3,619; 2,913	2,367; 1,947	19,640; 11,883
	GC content is (%)	36.36; 36.36	38.71; 38.71	39.10; 39.10	37.95; 37.95
stLFR, Supernova	Total number (>)	29,152; 60,975	85,695; 112,802	152,286; 195,010	17,321; 36,693
	Total length of (bp)	709,239,016; 688,839,198	779,204,818; 725,100,878	770,494,047; 730,575,457	317,378,019; 302,287,307
	Gap number (%)	2.88; 0	6.94; 0	5.18; 0	4.75; 0
	N50 Length (bp)	3,091,820; 81,434	98,101; 15,260	35,285; 13,869	2,492,536; 87,395
	N90 Length (bp)	23,260; 14,246	3,843; 3,039	1,501; 1,157	15,383; 12,958
	GC content is (%)	36.23; 36.23	38.81; 38.81	39.64; 39.64	37.67; 37.67
PacBio, Canu + polish x 2 + pilon x 2	Total number (>)	-	5,230; 5,230	6,499; 6,499	-
	Total length of (bp)	-	799,391,915; 799,391,915	771,667,536; 771,667,536	-
	Gap number (%)	-	0; 0	0; 0	-
	N50 Length (bp)	-	436,440; 436,440	315,740; 315,740	-
	N90 Length (bp)	-	74,251; 74,251	53,413; 53,413	-
	GC content is (%)	-	38.46; 38.46	38.22; 38.22	-

Compare & redundans & Merge & Gapcloser	Total number (>)	23,193; 33,220	5,282; 5,334	6,563; 6,628	10,764; 15,711
	Total length of (bp)	723,606,580; 702,564,625	807,385,834; 805,379,753	791,918,214; 789,758,988	337,427,279; 333,610,466
	Gap number (%)	2.91; 0	0.025; 0	0.027; 0	1.13, 0
	N50 Length (bp)	6,800,910; 87,281	440,804; 445,168	326,801; 330,037	3,227,123; 101,957
	N90 Length (bp)	23,479; 11,206	74,993; 75,736	55,085; 55,630	19,836; 12,120
	GC content is (%)	36.16; 36.16	38.56; 38.56	38.46; 38.46	37.95; 37.95
HiC, Hi-C Pro + Juicer + 3d-dna	Total number (>)	16,467; 24,158	885; 5,230	1,787; 6,499	7,165; 12,241
	Total length of (bp)	707,542,179; 685,045,514	799,826,415; 799,391,915	786,433,440; 784,077,440	331,750,784; 324,756,934
	Gap number (%)	3.18; 0	0.05; 0	0.3; 0	2.11; 0
	N50 Length (bp)	30,629,287; 131,011	52,515,067; 436,440	54,626,122; 323,566	18,565,976; 98,064
	N90 Length (bp)	37,644; 15,109	45,922,387; 74,251	33,716,155; 54,540	25,000; 12,864
	GC content is (%)	36.36; 36.36	38.46; 38.46	38.26; 38.26	37.92; 37.92
	Complete BUSCOs	97.00%	91.00%	92.20%	96.80%

Table S5 Global statistics of genome assembly and annotation of four *Begonia* species.

Species	*B. loranthoides*		*B. masoniana*		*B. darthvaderiana*		*B. peltatifolia*	
Type	Number	Size	Number	Size	Number	Size	Number	Size
**Assembly feature**								
Total scaffolds	16,502	707.55 Mb	885	799.83 Mb	1,787	786.43 Mb	7,169	331.75 Mb
Undetermined bases	-	22.50 Mb	-	0.43 Mb	-	2.36 Mb	-	6.99 Mb
Scaffold N50	9	33.31 Mb	7	52.52 Mb	6	54.63 Mb	7	20.51 Mb
Longest scaffold	-	57.64 Mb	-	84.93 Mb	-	89.91 Mb	-	42.45 Mb
GC content %	-	35.20	-	38.44	-	38.15	-	37.12
BUSCOs: complete; partial %	-	97.00; 0.80	-	91.00; 2.50	-	92.20; 1.50	-	96.80; 1.20
Pseudochromosomes	19	626.55 Mb	15	790.47 Mb	15	767.14Mb	15	289.05 Mb
% of Pseudochromosomes	-	88.55	-	98.83	-	97.55	-	87.13
**Genome annotation**								
Repetitive sequences %	-	66.52	-	68.40	-	70.33	-	51.47
Protein-coding genes	22,059	31.10 Mb	22,861	26.01 Mb	23,444	26.79 Mb	23,010	27.19 Mb
Gene density (genes per Mb)	-	31	-	28	-	29	-	69
Genes in pseudochromosomes	20,077	29.01 Mb	22,731	25.93 Mb	22,831	26.39 Mb	19,834	24.93 Mb
% of Genes in pseudochromosomes	91.02	93.29	99.43	99.68	97.39	98.51	86.2	91.69
Mean gene size (bp)	-	4307.81	-	3255.96	-	2928.33	-	2790.40
Mean CDS size (bp)	-	1409.91	-	1137.71	-	1142.63	-	1181.81
Number of exons	134,601	-	111,124	-	112,564	-	112,484	-
Mean exon size (bp)	-	231.06	-	234.05	-	237.98	-	139
Mean number of exons per gene	6.10	-	4.86	-	4.80	-	4.89	-
Number of introns	112,542	-	88,263	-	89,120	-	89,474	-
Mean intron size (bp)	-	568.01	-	548.65	-	469.75	-	413.68

Table S6 Statistics of raw data for whole genome sequencing and RNA-seq.

		Tissue	Male/Female	*B. loranthoides*	*B. masoniana*	*B. darthvaderiana*	*B. peltatifolia*
WGS	10 ×	leaf	-	184.87	181.09	199.34	184.72
	HiC	leaf	-	148.32	153.92	125.58	138.09
	stLFR	leaf	-	169.17	151.08	157.52	161.53
	PacBio	leaf	-	-	45.38	48.23	-
	Total			502.36	531.48	530.67	484.34
RNA	RNA-seq	root	-	-	-	5.12	27.92
		Stem/ rhizome	-	6.20	-	6.26	31.68
		flower	male	-	7.90	-	-
			female	-	10.06	-	34.17
		peduncle	-	-	-	-	18.25
		leaf	-	5.56	5.38	11.80	21.83
	Total			11.76	23.34	23.18	133.85

Note: For all data, the unit is Gb.

Table S7 Statistics of reads mapping to genome sequences for RNA-seq data from different tissues for four *Begonia* species.

Tissue	Total Mapped Reads	Perfect Match	Unique Match	Total Unmapped Reads
B. loranthoides				
leaf	97.05%	82.08%	88.35%	2.95%
stem	96.18%	82.54%	87.80%	3.82%
B. masoniana				
leaf	98.66%	68.29%	74.12%	1.34%
male_flower	98.76%	62.74%	78.01%	1.24%
female_flower	98.83%	56.31%	76.89%	1.17%
B.darthvaderiana				
root	90.92%	70.59%	73.22%	9.08%
stem	93.80%	74.49%	77.61%	6.20%
leaf	98.79%	69.62%	79.89%	1.21%
B. peltatifolia				
root	95.09%	70.00%	81.98%	4.91%
rhizome	97.94%	81.31%	93.65%	2.06%
leaf	98.30%	82.82%	92.64%	1.70%
peduncle	96.57%	81.35%	91.71%	1.38%
female flower	98.62%	82.56%	94.67%	3.43%

Table S8 Repetitive elements in four *Begonia* genomes. (See separate Excel file)

Table S9 Summary of genomes information across 13 representative angiosperms.
(See separate Excel file)

Table S10 Gene Ontology (GO) terms enrichment analysis of the expanded gene families of *Begonia*.

GO_ID	GO_Term	Adjusted P value
GO:0008270	zinc ion binding	9.79E-222
GO:0046914	transition metal ion binding	1.46E-123
GO:0046872	metal ion binding	3.54E-63
GO:0043169	cation binding	3.72E-60
GO:0005488	binding	2.60E-17
GO:0043167	ion binding	1.12E-12
GO:0003676	nucleic acid binding	6.11E-11
GO:0005216	ion channel activity	9.57E-10
GO:0022838	substrate-specific channel activity	9.57E-10
GO:0005515	protein binding	1.04E-09
GO:0015267	channel activity	5.83E-08
GO:0022803	passive transmembrane transporter activity	5.83E-08
GO:0003674	molecular_function	0.000568681
GO:0015075	ion transmembrane transporter activity	0.001926018
GO:0022891	substrate-specific transmembrane transporter activity	0.002634072
GO:0022892	substrate-specific transporter activity	0.004738534
GO:0006811	ion transport	0.023784422
GO:0035556	intracellular signal transduction	0.02401377

Table S11 Kyoto Encyclopedia of Genes and Genomes (KEGG) enrichment analysis of the expanded gene families of *Begonia*.

MapID	MapTitle	Adjusted P value
map00190	Oxidative phosphorylation	1.10E-286
map04144	Endocytosis	1.31E-96
map00240	Pyrimidine metabolism	2.80E-44
map00770	Pantothenate and CoA biosynthesis	8.33E-32
map00410	beta-Alanine metabolism	1.50E-26
map00020	Citrate cycle (TCA cycle)	2.14E-18
map00010	Glycolysis / Gluconeogenesis	8.20E-18
map01230	Biosynthesis of amino acids	1.06E-14
map03018	RNA degradation	1.17E-07
map00400	Phenylalanine, tyrosine and tryptophan biosynthesis	1.83E-07
map01200	Carbon metabolism	1.04E-05
map00052	Galactose metabolism	2.47E-04
map00040	Pentose and glucuronate interconversions	5.87E-04
map00520	Amino sugar and nucleotide sugar metabolism	1.24E-02

Table S12 Number of genes in families related to defense in *Begonia* and other selected genomes. Fields highlighted in orange and grey reprensented the families with expanding and contracting gene number significantly (*P*-value < 0.05).

Type	Gene	Pfam_id	Blor	Bmas	Bdar	Bpel	Csat	Atha	Gmax	Osat	Vvin	*P*-value
TFs	HSF	PF00447	20	16	21	18	21	30	52	35	19	0.049574
	AP2	PF00847	172	172	170	163	145	250	395	228	112	0.157184
	NAC	PF01849	5	4	7	5	4	9	12	10	4	0.100848
	WRKY	PF03106	95	88	89	94	67	165	215	206	70	0.087249
	CBF	PF03914	4	2	3	3	4	3	6	3	2	0.237901
LEA	LEA-5	PF00477	1	2	2	1	2	3	5	2	3	0.026106
	LEA-4	PF02987	4	1	2	0	7	52	15	5	5	0.084851
	LEA-2	PF03168	39	34	50	46	19	55	43	72	16	0.457927
	LEA-3	PF03242	7	6	7	6	5	10	11	6	4	0.323251
	LEA-1	PF03760	3	7	3	3	4	3	7	5	5	0.266129
	LEA-6	PF10714	1	1	3	2	2	4	2	2	0	0.380983
HSPs	HSP20	PF00011	23	20	22	19	36	30	82	40	41	0.027436
	HSP70	PF00012	29	25	28	23	16	37	59	32	38	0.108492
	Cpn60	PF00118	32	31	31	31	20	48	50	38	25	0.228086
	HSP90	PF00183	6	8	7	7	7	12	25	10	15	0.046089
	DnaJ	PF00226	89	78	84	80	71	196	191	131	69	0.075949
Antioxidant	GST	PF00043	10	12	16	11	24	33	55	60	39	0.005104
	Sod_Cu	PF00080	6	8	7	6	6	8	7	8	8	0.167778
	CAT/Catalase	PF00199	2	3	4	4	5	10	4	3	3	0.131495
	GPX/GSHPx	PF00255	6	9	12	8	7	11	16	5	5	0.492176
	PRX	PF10417	1	3	5	3	2	3	5	3	4	0.347171

	ATG8	PF02991	8	8	7	8	5	17	13	9	6	0.186309
	APG5	PF04106	1	1	2	1	2	1	2	1	1	0.340728
	APG17	PF04108	4	4	2	2	1	1	0	1	1	0.012839
	APG9	PF04109	1	1	1	1	2	1	4	2	1	0.070964
	APG12	PF04110	1	0	1	1	1	6	2	1	1	0.106573
	APG6	PF04111	1	0	1	1	1	4	2	4	1	0.035458
	ATG16	PF08614	2	1	1	1	1	1	2	1	1	0.44044
	ATG_C	PF09333	1	1	2	1	1	3	2	1	1	0.242155
	ATG27	PF09451	1	1	1	1	0	3	2	2	1	0.152279
	ATG13	PF10033	2	3	2	2	1	4	6	3	1	0.241179
	ATG14	PF10186	2	2	1	1	1	6	2	2	1	0.199362
	ATG11	PF10377	2	1	0	1	1	1	2	1	2	0.219359
	ATG3	PF10381	1	0	1	1	1	1	2	3	1	0.059165
Autophagy	ATG2_CAD	PF13329	1	0	2	1	0	3	2	1	0	0.393587
	P450	PF00067	237	158	186	151	195	368	464	385	414	0.006337
	Lyase_aromatic	PF00221	4	5	6	5	12	5	8	10	30	0.07179
	Bet V 1	PF00407	32	43	44	17	42	47	47	8	31	0.460287
	NB-ARC	PF00931	0	0	0	0	0	0	0	0	0	-
Biotic stress	TIR	PF01582	1	1	0	1	23	314	218	3	32	0.066891
	M1o	PF03094	28	23	26	23	22	35	46	22	21	0.225183
	Terpene_synth_C	PF03936	46	18	20	15	27	52	37	69	83	0.027708
	BBE	PF08031	14	21	14	11	6	33	49	12	16	0.181996
	Protein kinase domain	PF00069	629	532	545	539	530	1359	1418	1549	634	0.032709
drought tolerance	Dehydrin	PF00257	7	5	7	4	5	16	6	8	2	0.267837

	CBS domain containing protein (DCPS)	PF00571	54	53	65	60	42	75	97	114	38	0.184816
	Univeral stress protein family	PF00582	42	39	38	37	31	73	70	61	32	0.095746
	BTB/POZ domain	PF00651	49	37	41	47	33	116	99	155	41	0.060388
	Sodium/hydrogen exchanger family	PF00999	38	34	37	39	36	60	68	34	32	0.148646
	NAD dependent epimerase/dehydratase family	PF01370	37	34	45	36	31	60	72	80	59	0.02629
	GatB domain	PF02637	1	1	2	1	2	3	2	2	2	0.012106
	Thioesterase superfamily /4HBT	PF03061	4	6	5	5	9	15	17	12	10	0.00288
	C1 domain	PF03107	26	127	163	56	50	872	32	31	15	0.281684
	Nop14-like family	PF04147	1	1	1	1	1	2	3	3	1	0.044505
	Phosphoesterase family	PF04185	6	8	6	6	5	7	9	5	6	0.457413
	U-box domain	PF04564	66	62	61	56	51	97	126	101	48	0.099756
	hAT family dimerisation domain	PF05699	34	13	9	5	1	34	199	90	9	0.117547
	Protein tyrosine	PF07714	262	245	221	217	290	766	997	744	497	0.012553

	kinase										
	WRC	PF08879	22	20	15	18	11	23	37	29	18	0.17583
	QLQ	PF08880	16	11	12	14	8	20	28	20	9	0.193223
Others	Multicopper oxidase/Cu-oxidase	PF00394	30	31	30	29	35	63	97	52	65	0.016574
	ACBP	PF00887	4	4	3	7	4	16	11	7	6	0.058907
	MATE	PF01554	117	84	83	83	70	183	218	205	125	0.033405
	COBRA	PF04833	11	10	10	11	10	18	24	13	11	0.072316
	EDR1	PF14381	15	8	8	10	8	20	26	18	9	0.085159
	ABC_trans_N	PF14510	14	10	9	11	13	31	27	27	27	0.004101

Abbreviations: TFs = Transcription factors; LEA = Late Embryogenesis Abundant protein; HSPs = Heat shock proteins; Blor= *B. loranthoides*; Bmas = *B. masoniana*; Bdar = *B. darthvaderiana*; Csat = *Cucumis sativus*; Atha = *Arabidopsis thaliana*; *Gmax = Glycine max*; *Osat = Oryza sativa*; *Vvin = Vitis vinifera.*

Table S13 Statistics and annotations of the contracted gene families in *Begonia*.
(See separate Excel file)

Table S14 The significantly enriched GO terms of biological processes for genes with TE inserting in introns across four *Begonia* species.

GO_ID	GO_Term	*P*-value
B. loranthoides		
GO:0098656	anion transmembrane transport	0.0127
GO:0003333	amino acid transmembrane transport	0.0152
GO:1903825	organic acid transmembrane transport	0.0152
GO:1905039	carboxylic acid transmembrane transport	0.0152
GO:0015977	carbon fixation	0.0418
GO:0010112	regulation of systemic acquired resistance	0.0424
GO:0002376	immune system process	0.0424
GO:0002682	regulation of immune system process	0.0424
GO:0002831	regulation of response to biotic stimulus	0.0424
GO:0006955	immune response	0.0424
GO:0009627	systemic acquired resistance	0.0424
GO:0009814	defense response, incompatible interaction	0.0424
GO:0031347	regulation of defense response	0.0424
GO:0032101	regulation of response to external stimulus	0.0424
GO:0043900	regulation of multi-organism process	0.0424
GO:0045087	innate immune response	0.0424
GO:0045088	regulation of innate immune response	0.0424
GO:0050776	regulation of immune response	0.0424
GO:0080134	regulation of response to stress	0.0424
B. masoniana		
GO:0009607	response to biotic stimulus	0.0000
GO:0006950	response to stress	0.0001
GO:0006952	defense response	0.0006
GO:0006415	translational termination	0.0025
GO:0022411	cellular component disassembly	0.0026
GO:0032984	macromolecular complex disassembly	0.0026
GO:0043241	protein complex disassembly	0.0026
GO:0043624	cellular protein complex disassembly	0.0026
GO:0071822	protein complex subunit organization	0.0075
GO:0050896	response to stimulus	0.0142
B. darthvaderiana		
GO:0055114	oxidation-reduction process	0.0000
GO:0009772	photosynthetic electron transport in photosystem II	0.0000
GO:0009767	photosynthetic electron transport chain	0.0000
GO:0019684	photosynthesis, light reaction	0.0000
GO:0022900	electron transport chain	0.0000
GO:0060249	anatomical structure homeostasis	0.0000
GO:0044699	single-organism process	0.0014
GO:0001101	response to acid chemical	0.0108

GO:1901700	response to oxygen-containing compound	0.0108
GO:0051603	proteolysis involved in cellular protein catabolic process	0.0183
GO:0044257	cellular protein catabolic process	0.0183
GO:0031047	gene silencing by RNA	0.0199
GO:0016458	gene silencing	0.0199
GO:0030163	protein catabolic process	0.0213
GO:0010035	response to inorganic substance	0.0221
GO:0009415	response to water	0.0252
GO:0051276	chromosome organization	0.0310
GO:0009057	macromolecule catabolic process	0.0316
GO:0044265	cellular macromolecule catabolic process	0.0316
GO:0009892	negative regulation of metabolic process	0.0325
GO:0010605	negative regulation of macromolecule metabolic process	0.0325
GO:0010629	negative regulation of gene expression	0.0325
B. peltatifolia		
GO:0006298	mismatch repair	0.0020
GO:0006413	translational initiation	0.0153
GO:0009415	response to water	0.0170
GO:0006259	DNA metabolic process	0.0226
GO:1901700	response to oxygen-containing compound	0.0237
GO:0006950	response to stress	0.0281
GO:0010035	response to inorganic substance	0.0298
GO:0009628	response to abiotic stimulus	0.0366

Table S15 The significantly enriched GO terms of biological processes for genes with TEs inserting in promoter across four *Begonia* species.

GO_ID	GO_Term	P-value
B. loranthoides		
GO:0015979	photosynthesis	2.06E-07
GO:0009767	photosynthetic electron transport chain	0.0002
GO:0009772	photosynthetic electron transport in photosystem II	0.0002
GO:0019684	photosynthesis, light reaction	0.0006
GO:0017148	negative regulation of translation	0.0022
GO:0034249	negative regulation of cellular amide metabolic process	0.0022
GO:0032269	negative regulation of cellular protein metabolic process	0.0056
GO:0051248	negative regulation of protein metabolic process	0.0056
GO:0022900	electron transport chain	0.0082
GO:0009890	negative regulation of biosynthetic process	0.0331
GO:0010558	negative regulation of macromolecule biosynthetic process	0.0331
GO:0031327	negative regulation of cellular biosynthetic process	0.0331
GO:0051172	negative regulation of nitrogen compound metabolic process	0.0331
GO:2000113	negative regulation of cellular macromolecule biosynthetic process	0.0331
GO:0031324	negative regulation of cellular metabolic process	0.0494
B. masoniana		
GO:0015074	DNA integration	2.79E-05
GO:0009607	response to biotic stimulus	2.79E-05
GO:0006952	defense response	0.0013
GO:0006950	response to stress	0.0210
GO:0006415	translational termination	0.0210
GO:0022411	cellular component disassembly	0.0222
GO:0032984	macromolecular complex disassembly	0.0222
GO:0043241	protein complex disassembly	0.0222
GO:0043624	cellular protein complex disassembly	0.0222
GO:0009772	photosynthetic electron transport in photosystem II	0.0232
GO:0009767	photosynthetic electron transport chain	0.0266
GO:0006808	regulation of nitrogen utilization	0.0266
GO:0017148	negative regulation of translation	0.0266
GO:0019740	nitrogen utilization	0.0266
GO:0034249	negative regulation of cellular amide metabolic process	0.0266
B. darthvaderiana		
GO:0000723	telomere maintenance	3.69E-19
GO:0032200	telomere organization	3.69E-19
GO:0060249	anatomical structure homeostasis	3.69E-19

GO:0009767	photosynthetic electron transport chain	3.98E-16
GO:0009772	photosynthetic electron transport in photosystem II	3.81E-14
GO:0019684	photosynthesis, light reaction	1.40E-13
GO:0006259	DNA metabolic process	7.71E-13
GO:0022900	electron transport chain	2.36E-11
GO:0015979	photosynthesis	1.35E-07
GO:0051276	chromosome organization	3.46E-07
GO:0006281	DNA repair	7.80E-07
GO:0006974	cellular response to DNA damage stimulus	1.21E-06
GO:0033554	cellular response to stress	1.51E-06
GO:0006091	generation of precursor metabolites and energy	1.91E-06
GO:0051716	cellular response to stimulus	2.41E-06
GO:0006996	organelle organization	3.48E-06
GO:0015074	DNA integration	6.78E-06
GO:0042592	homeostatic process	6.96E-06
GO:0055114	oxidation-reduction process	1.60E-05
GO:0065008	regulation of biological quality	0.0002
GO:0006950	response to stress	0.0007
GO:0050896	response to stimulus	0.0222
GO:0009892	negative regulation of metabolic process	0.0284
GO:0010605	negative regulation of macromolecule metabolic process	0.0284
GO:0010629	negative regulation of gene expression	0.0284
B. peltatifolia		
GO:0015074	DNA integration	5.23E-25
GO:0006259	DNA metabolic process	9.23E-14
GO:0006412	translation	4.93E-06
GO:0043043	peptide biosynthetic process	6.01E-06
GO:0006518	peptide metabolic process	9.83E-06
GO:0043604	amide biosynthetic process	1.09E-05
GO:0043603	cellular amide metabolic process	2.03E-05
GO:0015979	photosynthesis	0.0002
GO:0034641	cellular nitrogen compound metabolic process	0.0002
GO:0006807	nitrogen compound metabolic process	0.0006
GO:1901566	organonitrogen compound biosynthetic process	0.0006
GO:0009767	photosynthetic electron transport chain	0.0010
GO:0043170	macromolecule metabolic process	0.0026
GO:0019684	photosynthesis, light reaction	0.0035
GO:0044237	cellular metabolic process	0.0074
GO:0006298	mismatch repair	0.0095
GO:1901564	organonitrogen compound metabolic process	0.0107
GO:0044260	cellular macromolecule metabolic process	0.0242
GO:0022900	electron transport chain	0.0253
GO:0035434	copper ion transmembrane transport	0.0408
GO:0006281	DNA repair	0.0452

Table S16 Chlorophyll data of the sun loving plant *Gerbera hybrida* and four *Begonia* species.

Species	Content (μg cm^2)			
	Chl a	Chl b	Total Chl	Chl a/b
Gerbera hybrida	34.48±2.60	11.08±0.90	45.57±3.50	3.11±0.02
B. loranthoides	27.98±1.90	10.30±0.90	38.28±2.78	2.72±0.08
B. peltatifolia	26.70±5.92	9.89±2.80	36.59±8.72	2.73±0.16
B. masoniana	17.60±1.89	8.34±0.89	25.95±2.77	2.11±0.03
B. darthvaderiana	19.23±1.06	9.02±0.95	28.24±1.19	1.98±0.03

Note: Values are means for three biological replicates (±SD).

Table S17 Comparisons of the gene numbers for the light signaling genes in 10 angiosperm genomes.

Gene	Vvin	Mcha	Csat	Lsic	Clan	Atha	Blor	Bmas	Bdar	Bpel
**>Blue light and UV-A signaling pathway**										
Cryptochromes (CRY1/2)	2	2	1	1	2	2	4	3	4	4
Cullin 4 (CUL4)	1	1	1	1	1	1	4	2	2	2
CONSTITUTIVELY PHOTOMORPHOGENIC1 (COP1)	2	2	3	2	2	1	3	2	1	2
COP9 signalosome (CSN)	1	1	1	2	1	1	2	2	3	2
CONSTITUTIVE PHOTOMORPHOGENIC 10 (COP10)	0	1	0	0	1	1	0	1	1	1
DE-ETIOLATED 1 (DET1)	1	1	0	1	1	1	1	0	2	0
DNA-damage-binding protein 1 (DDB1)	1	0	1	1	1	1	1	1	1	1
**>UV-B signaling pathway**										
UV Resistance Locus 8 (UVR8)	1	1	1	1	1	1	2	2	3	2
SUPPRESSOR OF PHYAs (SPAs)	7	2	3	2	3	4	3	4	5	4
ELONGATED HYPOCOTYL 5 (HY5)	2	2	1	2	2	2	3	2	2	2
**>Red/Far-red light signaling pathway**										
Phytochromes (PHAs)	1	1	2	2	2	1	1	1	1	1
Phytochromes (PHYB/C/D/E)	3	2	2	3	4	4	6	5	5	5
PHYTOCHROME INTERACTING FACTORs (PIFs)	5	8	8	7	5	7	9	7	9	6

ETHYLENE INSENSITIVE3/EIN3-LIKE1 (EIN3/EIL1)	2	4	4	4	4	4	3	4	4	4
**>Seeding emergence signaling pathway**										
EIN3-BINDING F-BOX1 and 2 (EBF1/2)	3	5	4	6	4	2	6	4	4	3
FAR-RED ELONAGTED HYPOCOTYL 1/ FHY1-LIKE (FHY1/FHL)	1	2	2	1	2	2	1	1	1	1
**>PHYA nuclear transfer feedback loop**										
FAR-RED ELONGATED HYPOCOTYLS3 & FAR-RED-IMPAIRED RESPONSE1 (FHY3&FAR1)	5	3	2	3	2	2	2	1	2	1
**>others**										
LONG AFTER FAR-RED RADIATION1 (LAF1)	3	1	3	2	2	1	1	0	0	2
Phototropins (PHOT1/2)	2	3	3	3	3	2	5	5	5	5
GA 20-oxidase (GA20ox)	8	10	8	9	8	5	7	5	5	7
Homo-box 2/4 (HB2/4)	2	1	2	1	1	2	2	2	0	2
YUCs	11	10	10	11	11	11	13	14	11	15
DELLA	7	3	5	6	8	5	10	11	11	10

Abbreviation: Vvin, *Vitis vinifera*; Mcha, *Momordica charantia*; Cast, *Cucumis sativus*; Lsic, *Lagenaria siceraria*; Clan, *Citrullus lanatus*; Atha, *Arabidopsis thaliana*; Blor, *B. loranthoides*; Bmas, *B. masoniana*; Bdar, *B. darthvaderiana*; Bpel, *B. peltatifolia*.

Table S18 Comparisons of the gene numbers of the light-harvesting chlorophyll a/b-binding proteins (LHCs) family genes in the seven genomes of *Begonia* and other angiosperms.

LHC family	Bmas	Bdar	Blor	Bpel	Csat	Atha	Osat
LHCA total	**9**	**9**	**11**	**8**	**6**	**7**	**6**
LHCA1	2	2	2	2	1	1	1
LHCA2	3	3	2	2	0	2	1
LHCA3	1	2	2	2	1	1	1
LHCA4	3	2	3	2	2	1	1
LHCA5	0	0	1	0	1	1	1
LHCA6	0	0	1	0	1	1	1
LHCB total	**24**	**23**	**16**	**13**	**10**	**15**	**9**
LHCB1	11	10	6	4	4	5	3
LHCB2	2	3	2	2	1	3	1
LHCB3	2	2	2	1	1	1	1
LHCB4	3	3	2	2	1	3	1
LHCB5	2	1	1	1	1	1	1
LHCB6	3	4	2	2	1	1	1
Chlab	1	0	1	1	1	1	1
Total	**33**	**32**	**27**	**21**	**16**	**22**	**15**

Abbreviation: Bmas, *B. masoniana*; Bdar, *B. darthvaderiana*; Bpel, *B. peltatifolia*; Blor, *B. loranthoides*; Cast, *C. sativus*; Atha, *A. thaliana*; Osat, *O. sativa*.
